# Glutamine addiction promotes glucose oxidation in triple-negative breast cancer

**DOI:** 10.1038/s41388-022-02408-5

**Published:** 2022-07-18

**Authors:** Lake-Ee Quek, Michelle van Geldermalsen, Yi Fang Guan, Kanu Wahi, Chelsea Mayoh, Seher Balaban, Angel Pang, Qian Wang, Mark J. Cowley, Kristin K. Brown, Nigel Turner, Andrew J. Hoy, Jeff Holst

**Affiliations:** 1 School of Mathematics and Statistics, The University of Sydney, Camperdown, NSW Australia; 2 Origins of Cancer Program, Centenary Institute, The University of Sydney, Camperdown, NSW Australia; 3 Sydney Medical School, The University of Sydney, Camperdown, NSW Australia; 4 School of Medical Sciences and School of Clinical Medicine, UNSW Sydney, Kensington, NSW Australia; 5 Children’s Cancer Institute, Lowy Cancer Centre, UNSW Sydney, Kensington, NSW Australia; 6 School of Women’s and Children’s Health, UNSW Sydney, Kensington, NSW Australia; 7 School of Medical Sciences, Charles Perkins Centre, The University of Sydney, Camperdown, NSW Australia; 8 Peter MacCallum Cancer Centre, Melbourne, VIC Australia; 9 The Sir Peter MacCallum Department of Oncology, The University of Melbourne, Melbourne, VIC Australia; 10 Department of Biochemistry and Pharmacology, The University of Melbourne, Melbourne, VIC Australia; 11 Department of Pharmacology, School of Medical Sciences, UNSW Sydney, Kensington, NSW Australia

**Keywords:** Cancer metabolism, Breast cancer

## Abstract

Glutamine is a conditionally essential nutrient for many cancer cells, but it remains unclear how consuming glutamine in excess of growth requirements confers greater fitness to glutamine-addicted cancers. By contrasting two breast cancer subtypes with distinct glutamine dependencies, we show that glutamine-indispensable triple-negative breast cancer (TNBC) cells rely on a non-canonical glutamine-to-glutamate overflow, with glutamine carbon routed once through the TCA cycle. Importantly, this single-pass glutaminolysis increases TCA cycle fluxes and replenishes TCA cycle intermediates in TNBC cells, a process that achieves net oxidation of glucose but not glutamine. The coupling of glucose and glutamine catabolism appears hard-wired via a distinct TNBC gene expression profile biased to strip and then sequester glutamine nitrogen, but hampers the ability of TNBC cells to oxidise glucose when glutamine is limiting. Our results provide a new understanding of how metabolically rigid TNBC cells are sensitive to glutamine deprivation and a way to select vulnerable TNBC subtypes that may be responsive to metabolic-targeted therapies.

## Introduction

Rapid cellular proliferation is metabolically demanding, necessitating large supplies of macro-nutrients. However, the full complement of amino acids are rarely accessible to cancer cells, forcing cells to leverage available substrates to re-balance the amino acid supply-and-demand network [1], with the tricarboxylic acid (TCA) cycle being the central interconversion hub [2]. The most abundant circulating amino acid, glutamine, is not only a versatile substrate for cellular energy and proliferation, but also facilitates uptake of other amino acids, nucleotide synthesis, pH balance, redox balance, signalling and detoxification [3–5]. Glutamine addiction is used to describe how many cancer cells show increased glutamine uptake and glutamine dependence [6, 7]. Somewhat surprisingly, this glutamine consumption is excessive: roughly 50-fold greater than the amount required for protein synthesis and 7-fold greater than the next most consumed amino acids (i.e. serine, leucine) among the NCI-60 cancer cell lines [8, 9], while only ~6% of cell mass is made from glutamine [5]. Glutamine consumption is especially pronounced in the triple-negative breast cancer (TNBC) subtype, distinguished by their upregulated expression of key genes related to glutamine utilisation and sensitivity to drugs inhibiting glutamine-related pathways [10–15]. However, high glutamine uptake does not imply dependency [16]; our previous work showed Luminal A breast cancer cell lines such as MCF-7 consumed nearly as much glutamine as TNBC cells despite being insensitive to glutamine uptake inhibition [14]. Furthermore, inhibiting glutamine-related pathways does not consistently reduce viability across all cancer cell lines, not even among glutamine-dependent TNBC cell lines [15, 17, 18]. It is unclear what metabolic processes cause glutamine to be consumed at amounts greatly exceeding biosynthetic demands, and how this relates to glutamine dependency.

In this study, we used isotopically labelled glutamine to resolve glutamine utilisation in two different breast cancer subtypes (TNBC vs. Luminal A), with the TNBC subtypes sensitive to inhibition of *SLC1A5*/ASCT2-mediated glutamine uptake. By contrasting the two subtypes, we show that high glutamine-to-glutamate overflow—via the TCA cycle—supports glucose metabolism in TNBC subtypes. Central to this process is the use of glutamine to drive acetyl-CoA oxidation through a series of amine exchanges, potentially a way to boost TCA cycle fluxes by harnessing nutrient gradients [19]. This elevates glucose oxidation in TNBC cells, but only when glutamine is available. Our work introduces a specific TCA cycle configuration, which we term “single-pass glutaminolysis”, to describe a single round of glutamine TCA cycle traversal that facilitates glucose catabolism. Phenotyping tumours for a rigid glucose-glutamine metabolic coupling as a vulnerability marker holds the potential for accurate identification of tumours sensitive to the inhibition of glutamine uptake and/or metabolism.

## Results

### Uptake of glutamine is in excess of biosynthetic demands

**Fig. 1:**
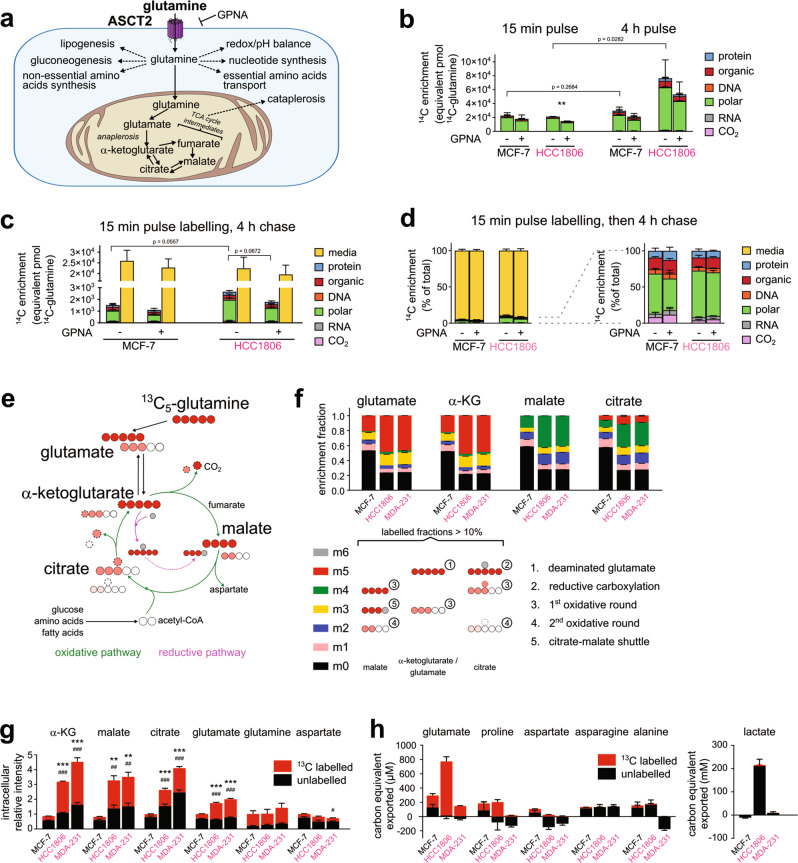
Metabolic signature of single-pass glutaminolysis in TNBC cells resolved by radiolabelled and stable-isotope labelled glutamine. **a** Schematic of glutamine utilisation. **b** ^14^C-enrichment in the dissolved CO_2_, RNA, DNA, polar, organic and protein phases extracted from MCF-7 and HCC1806 cells after 15 min and 4 h labelling with U-^14^C_5_-glutamine (all *n* = 3, except HCC1806 15 min *n* = 4). Data expressed as equivalent pmol of U-^14^C_5_-glutamine. *P* values calculated by one-tailed Student’s *t* test: **P < 0.01; by two-tailed Student’s *t* test: displayed. **c** ^14^C enrichment after 4 h unlabelled chase following 15 min pulse with U-^14^C_5_-glutamine (*n* = 3). Chase media included. **d** ^14^C-enrichment data from **c** expressed as a percentage of total ^14^C signal detected. **e** Schematic of glutamine carbon transfer. Circles with decreasing red saturation indicates carbon traversing the TCA cycle 1^st^, 2^nd^ and 3^rd^ round; grey indicates unlabelled CO_2_. **f** Relative ^13^C enrichment fraction of TCA cycle metabolites and glutamate measured by GC-MS after 14 h incubation in U-^13^C_5_-glutamine (*n* = 3). m_2_, m_3_, m_4_ and m_5_ fractions shown with brief explanation of how these isotopologues were derived in **e**. ^13^C data corrected for natural isotopic enrichment. **g** Data in **f** shown as total abundance of ^13^C labelled and unlabelled carbon expressed relative to MCF-7. *P* values calculated by two-tailed Student’s *t* test with respect to MCF-7: for total: #*P* < 0.05, ##*P* < 0.01, ###*P* < 0.001; for ^13^C-labelled: **P < 0.01; ***P < 0.001. **h** Amount of ^13^C labelled and unlabelled metabolites exported into the culture media after 14 h incubation in U-^13^C_5_-glutamine, expressed in carbon-equivalent units to allow comparison; negative indicates uptake (*n* = 3). See also Supplementary Fig. S1.

Glutamine is oxidised for energy, but also supports a large spectrum of metabolic functions (Fig. 1a). Using radiolabelled U-^14^C_5_-glutamine, we analysed the partitioning of glutamine carbon into six cellular fractions: intracellular acid-soluble metabolites (polar), chloroform-soluble metabolites (organic), RNA, DNA, protein and extracellular dissolved ^14^CO_2_ (Supplementary Fig. S1a) [5]. Both HCC1806 (TNBC) and MCF-7 (Luminal A) cell lines showed comparable glutamine uptake rates based on ^14^C enrichment at the 15-minute mark (Fig. 1b). Consistent with our previous data [14], ^14^C-glutamine was predominantly partitioned into the polar fraction in both cell lines (>88%), followed by a much smaller organic fraction (<10%) (Supplementary Fig. S1b, c). ^14^C-labelling of RNA, DNA, protein and CO_2_ was negligible (<1%). The ASCT2 (glutamine transporter) inhibitor GPNA reduced ^14^C enrichment at the 15-minute mark by 20–30%, particularly in HCC1806 cells that we have previously shown to experience a greater growth suppression [14] (Fig. 1b). Intracellular partitioning of glutamine was not altered (Supplementary Fig. S1c).

HCC1806 cells accumulated up to 3.6 times more glutamine carbons after 4 h than at 15-minute timepoint, whereas MCF-7 cells showed no significant increase (Fig. 1b). This shows HCC1806 cells have a greater reservoir of polar metabolites derived from glutamine. The soluble polar fraction remained the largest (>75% total) in both cell lines (Supplementary Fig. S1c). A 15-minute ^14^C-pulse followed by 4-hour chase showed the vast majority (>89%) of glutamine carbon was released back into the extracellular media (Fig. 1c, d). HCC1806 cells appeared to retain more polar metabolites compared to MCF-7 cells (Fig. 1c), which may suggest a greater conversion of glutamine into soluble forms retained intracellularly despite, comparable glutamine uptake rates [13, 20, 21]. Inhibition of the cystine/glutamate transporter *SLC7A11*/xCT (by sulfasalazine) significantly reduced ^14^C label efflux during the chase, with no effect for L-type amino acid transporter 1 (*SLC7A5*/LAT1) inhibitors BCH or benzylserine (Supplementary Fig. S1d). These ^14^C-enrichment data reinforce the notion that glutamine consumption exceeds biosynthetic demand and is accumulated as exportable polar metabolites such as glutamate, in particular in TNBC cells.

### Single-pass glutaminolysis paves a TCA cycle thoroughfare in TNBC cells

The glutamine-derived soluble fraction is comprised of TCA cycle intermediates, lactate and non-essential amino acids such as glutamate, aspartate, proline, alanine and even glutamine itself, with each demarcating the conversion route(s) of glutamine (Fig. 1a). To determine which pathway(s) contribute to excessive glutamine consumption, we performed steady-state ^13^C-labelled stable isotope tracing and resolved where glutamine carbons reside (Fig. 1e). GC-MS was used to quantify metabolism of U-^13^C_5_-glutamine into TCA cycle metabolites and amino acids following a 14-hour exposure (to ensure steady state [22]).

There was a greater contribution of glutamine to the production of glutamate, α-ketoglutarate (αKG), malate and citrate in TNBC cell lines (HCC1806 and MDA-MB-231) than MCF-7 cells inferred from the higher relative ^13^C-enrichments (Fig. 1f). Unlabelled fractions (*black*: ^12^C) represent metabolites produced from non-glutamine sources (or pre-existing intracellular glutamine) and were more abundant in MCF-7 cells. The m_4_ isotopologues of malate and citrate were the major fractions in TNBC (*green*: ^13^C_4_-malate, ^13^C_4_-citrate). This suggests that malate and citrate were largely produced from glutamine carbons that had traversed the TCA cycle only one round. As these ^13^C measurements were made at isotopic steady-state, they imply the majority of glutamine carbon exited the TCA cycle at some intermediate without being catalysed by αKG dehydrogenase a second time. We named this metabolic configuration single-pass glutaminolysis. By contrast, malate and citrate in MCF-7 cells had greater proportions of m_1_ and m_2_ fractions (*blue*, *pink*), with less m_4_ fraction compared to TNBC cells, indicating relatively more glutamine carbon in MCF-7 cells underwent complete oxidation by traversing multiple rounds in the TCA cycle (Fig. 1f). Importantly, the TNBC-specific single-pass glutaminolysis configuration was confirmed when another Luminal A cell line, T47D, was analysed (Supplementary Fig. S1e).

Next, we quantified key metabolites that exchange with TCA cycle metabolites to determine where glutamine carbons exited prior to being a substrate for αKG dehydrogenase a second time. Alanine, aspartate and lactate showed minimal glutamine carbon labelling compared to the TCA cycle metabolites (Fig. 1g, h, Supplementary Fig. S1f, g and Supplementary Table S1). The lack of alanine and lactate enrichment indicated negligible flux out of TCA through malic enzyme, which is needed to fully breakdown glutamine carbon into CO_2_. Without other significant outlets, αKG is both the main entry and exit point in single-pass glutaminolysis, which is an unorthodox TCA cycle configuration because it achieves no net conversion of glutamine/glutamate carbon into another product.

In TNBC cell lines, glutamate and αKG labelling consisted of approximately equal parts of fully labelled m_5_ (~50%) and of partially labelled fractions (m_0_ to m_4_) (Fig. 1f). This indicated the two converging streams producing αKG, i.e. the stripping of glutamine nitrogen into αKG and the decarboxylation of citrate into αKG, were similar in magnitude. As αKG level is balanced, the effluxes at αKG must also be equally split between transaminating αKG back into glutamate and decarboxylating αKG into succinyl-CoA. In the case where metabolic channelling prevails, then this subnetwork could be strung into a linear pathway, with the driving force of glutamine anaplerosis pushing αKG through the TCA cycle one complete round before being effluxed as glutamate. Achieving these higher TCA cycle fluxes compensates for TNBC’s impaired ability to fully oxidise glutamine.

Another function served by single-pass glutaminolysis is building up TCA cycle metabolites using extracellularly abundant glutamine. TCA cycle metabolites were not only more glutamine-derived in TNBC cells, but they were also consistently greater in abundance, with levels up to 4-fold higher than MCF-7 cells (Fig. 1g, Supplementary Fig. S1f). Thus, glutamine flow-through buffers the supply of TCA cycle metabolites, potentially by leveraging the concentration gradient of exogenous glutamine. Potentially, this is correlated to glutamate enrichment, i.e. higher abundance of intracellular glutamate relative to glutamine, a feature observed in TNBC cells for both our data and the broader CCLE dataset (Supplementary Fig. S1h) [23]. This bias has been observed elsewhere, and is associated with glutaminase (GLS) expression in the cell lines [15, 24, 25]. The downside is wasteful accumulation of glutamate, since exported glutamate was by far the most abundant glutamine-derived by-product in TNBC cells (Fig. 1h), which is also present in NCI-60 TNBC cell lines (Supplementary Fig. S1i) [9].

Together with our ^14^C data, we show how single-pass glutaminolysis engages the TCA cycle and that glutamine-to-glutamate overflow is not merely due to an “overactive” glutaminase. Importantly, the use of glutamine to build up TCA cycle metabolites is a distinguishing phenomenon among TNBC cell lines.

### Glutamine utilisation in 2D cultures is reproduced in tumour explants

**Fig. 2:**
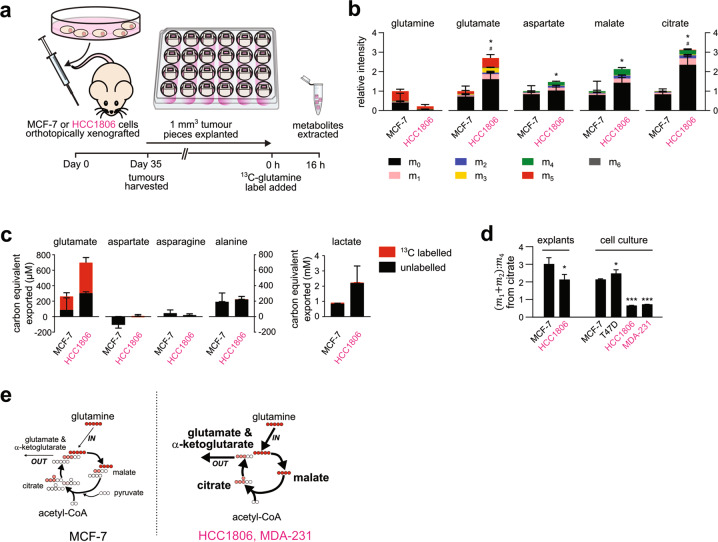
Tumour explants also exhibit ^13^C metabolite signatures of single-pass glutaminolysis. **a** Workflow to generate metabolite data for HCC1806 and MCF-7 tumours explants cultured for 16 h in U-^13^C_5_-glutamine. **b** Intracellular abundance of metabolite isotopologues measured in tumour explants expressed relative to MCF-7 (*n* = 3). *P* values calculated by two-tailed Student’s *t* test with respect to MCF-7: for total: #*P* < 0.05; for ^13^C-labelled: *P < 0.05. **c** Amount of ^13^C labelled and unlabelled metabolites exported into the explant culture media after 16 h incubation in U-^13^C_5_-glutamine, expressed in carbon-equivalent units to allow comparison; negative indicates uptake (*n* = 3). **d** Isotopologue ratios of citrate from **b**, Fig. 1f and Supplementary Fig. S1e represent the proportion of glutamine carbon undergoing further oxidation, making the 2^nd^ and 3^rd^ round in the TCA cycle (*n* = 3; T47D *n* = 6). *P* values calculated by two-tailed Student’s *t* test with respect to MCF-7: *P < 0.05, **P < 0.01. **e** Qualitative representation of how subtypes differed in TCA cycle flux configuration. Thicker arrows and font indicate higher flux and greater abundance, respectively.

Single-pass glutaminolysis may be a cell culture artefact induced by the supraphysiological levels of glutamine in culture media (2 mM) compared to in vivo (~0.5 mM) [26]. We examined ^13^C-tracer data from 3D ex vivo tumour explant culture in combination with a more physiologically restrictive human plasma-like medium (Fig. 2a) [27, 28]. The previously observed patterns of glutamine utilisation were preserved under these more physiological conditions, including HCC1806 explants exhibiting a greater abundance of glutamate and TCA cycle metabolites, a build-up of glutamine-derived carbon, and a greater m_4_ enrichment at citrate and malate (Fig. 2b). Similarly, these data recapitulated the glutamine-to-glutamate overflow and the lack of glutamine label in lactate and alanine (Fig. 2c). However, explants overall showed lower ^13^C-label incorporation than cell cultures, possibly due to both lower glutamine media concentrations and the 3D culture format contributing to increased glucose utilisation [29].

In this setting, we used the (m_1_ + m_2_):m_4_ ratio from citrate to compare glutamine oxidation between HCC1806 and MCF-7 explants, as well as data from cell cultures (Fig. 2d). Effectively the ratio indicates how much of the recirculated glutamine carbon (m_4_) is oxidised further by the TCA cycle. The lower ratio in HCC1806 than MCF-7 explants indicated less of the recirculated glutamine carbon was further oxidised and thus a greater presence of single-pass glutaminolysis. The difference in ratios were even greater for 2D cell cultures, with a significant reduction in MCF-7 and HCC1806 ratios compared to MCF-7 and T47D (Fig. 2d).

Consistent results between our ex vivo (3D) and in vitro (2D) experiments verified the tendency for TNBC cells to conduct single-pass glutaminolysis and boost TCA cycle fluxes and metabolite abundances (Fig. 2e).

### Nitrogen balancing in TNBC pushes glutamate into TCA, pulls out αKG

**Fig. 3:**
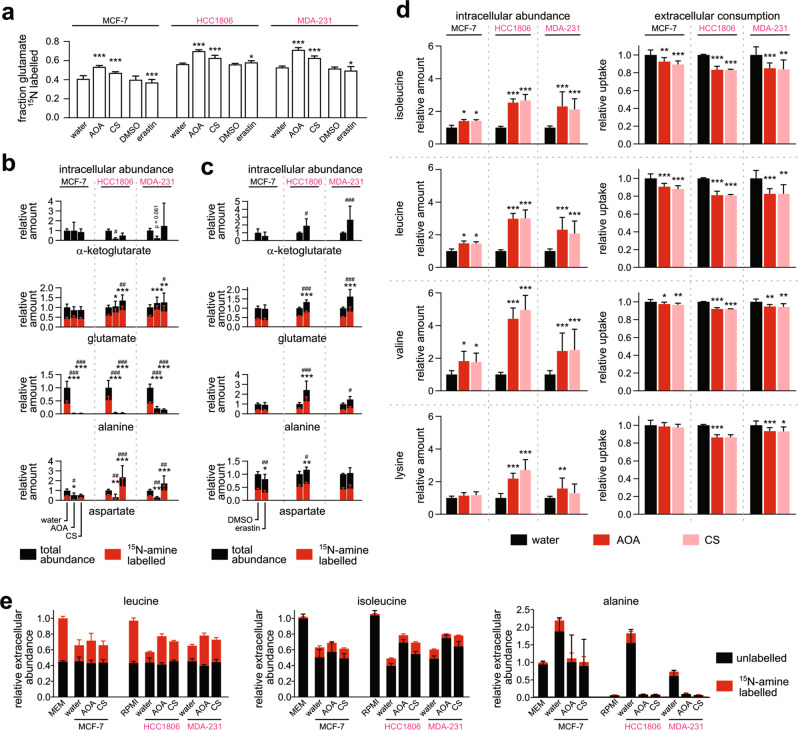
EAA and NEAA aminotransferases are key enzymes facilitating single-pass glutaminolysis. **a** The fraction of ^15^N-labelled intracellular glutamate when cells were cultured in ^15^N-amine-glutamine for 16 h together with inhibitors AOA, CS or erastin. Water and DMSO (for erastin) were used as controls. Intracellular abundance of ^15^N labelled and unlabelled metabolites when cells were treated with AOA or CS (**b**), or erastin (**c**). Intracellular abundances were normalised to either water or DMSO. *P* values calculated by two-tailed Student’s *t* test with respect to controls: for total: #*P* < 0.05, ##*P* < 0.01, ###*P* < 0.001; for ^13^C-labelled: *P < 0.05, **P < 0.01, ***P < 0.001. **d** The effects of 1 mM aminotransferase inhibitors aminooxyacetate (AOA) and cycloserine (CS) on the extracellular consumption and intracellular abundance of EAAs. **e** Abundance of ^15^N-labelled and unlabelled amino acids in extracellular media after 16 h incubation in ^15^N-leucine and with either AOA, CS or no inhibitor. Reported abundances of leucine and isoleucine normalised to starting media MEM and RPMI, and alanine to MEM. All data *n* = 9, except for ^15^N-leucine experiment *n* = 4. *P* values for **a** and **d** were calculated by two-tailed Student’s *t* test with respect to controls: *P < 0.05, **P < 0.01, ***P < 0.001. See also Supplementary Figs. S2 and S3.

Aminotransferases can act as gatekeepers to route glutamine through the TCA cycle. To test their contributions, we treated cells with the aminotransferase inhibitors aminooxyacetate (AOA) and cycloserine (CS) and performed tracing with ^15^N-amine labelled glutamine. Increased retention of ^15^N-labelled glutamate confirmed AOA and CS had partially inhibited transamination of αKG to glutamate that causes label dilution (Fig. 3a). After glutamate, alanine was the greatest ^15^N-amine sink among the non-essential amino acids (NEAA), and both AOA and CS treatment profoundly inhibited alanine production (Fig. 3b, Supplementary Fig. S2a).

TNBC cells suffered a greater suppression of EAA catabolism following AOA and CS exposure, as demonstrated by intracellular build-up and reduced media consumption (Fig. 3d, Supplementary Fig. S2b). This outcome could be linked to the depletion of αKG by AOA (and to a limited extent by CS) that was observed only in TNBC cells (Fig. 3b). These data suggest EAA breakdown in TNBC cells was constrained by αKG availability and coupled to alanine synthesis to replenish αKG. This process pushes glutamate into the TCA cycle.

Blocking glutamate export using the *SLC7A11*/xCT inhibitor erastin in TNBC cells created an intracellular backlog along 5-carbon metabolites glutamate, glutamine, αKG and proline, in addition to alanine and increased alanine export (Fig. 3c and Supplementary Fig. S2a). These effects were seen only in TNBC cells, most likely because TNBC cells export glutamate more than MCF-7 cells (Figs. 1h and 2c). Similarly, the shift from alanine to aspartate synthesis by CS (a D-alanine analogue) was only observed in TNBC cells (Fig. 3b). These distinct metabolic changes in response to aminotransferase inhibition suggest it is mainly TNBC cells that seek to sequester excess amine as amino acids.

The collective breakdown of EAAs generates excess amine. The accumulation of unlabelled glutamate (Fig. 3a, Supplementary Fig. S2a), despite incoming glutamine being almost fully ^15^N-amine labelled, is evidence of a non-trivial amount of EAA breakdown converting αKG to glutamate. Many of these pathways have an initial transamination step, such as BCAT1/2, TAT, AADAT and GOT1/2 (c.f., amino-lyase). There is a consensus among NCI-60 breast cancer cell lines from the CORE (metabolic consumption and release) dataset that EAAs are consumed in excess of biosynthesis [9] (Supplementary Fig. S3a). Almost all ratios calculated for CORE’s cell-specific EAA uptake relative to the composition of a generic human cell (Recon 3D) were above one [8]. A median ratio of 2.76 indicates half of the measured EAA uptake rates were at least 176% in excess.

In our hands, all three cell lines in culture had comparable baseline consumptions for the majority of EAAs, while glutamine consumption far exceeded all other amino acids (Supplementary Fig. S3b). Notably, ^13^C_6_-leucine tracing confirmed leucine oxidation in MCF-7 was not less than in TNBC cells (Supplementary Fig. S3c). If all cell lines deplete αKG at similar rates via EAA breakdown, then TNBC cells’ sensitivity to AOA and CS could be explained by their reliance on NEAA aminotransferases to regenerate αKG. However, TNBC cells’ reliance on NEAA aminotransferases is not because these enzymes have greater fluxes as the transfer of ^15^N label from leucine to extracellular isoleucine and alanine occurred at similar rates for all three cell lines (Fig. 3e). Isotopic steady-state was achieved within 6 h, indicating transamination is a rapid and reversible process (Supplementary Fig. S2c). As such, TNBC’s sensitivity to AOA and CS may be due to a lack of alternative pathways to regenerate αKG.

Put together, these data show NEAA and EAA aminotransferases are key components of single-pass glutaminolysis that push and pull glutamine through the TCA cycle.

### Rigid glucose-glutamine coupling drives TCA cycle fluxes in TNBC

The pairing of NEAA and EAA aminotransferases creates a stoichiometrically balanced reaction, thus does not require participation of the TCA cycle. This raised the question as to what is drawing αKG through the TCA cycle. To answer this, we shifted our attention to how glucose intersects with glutamine metabolism at the TCA cycle.

**Fig. 4:**
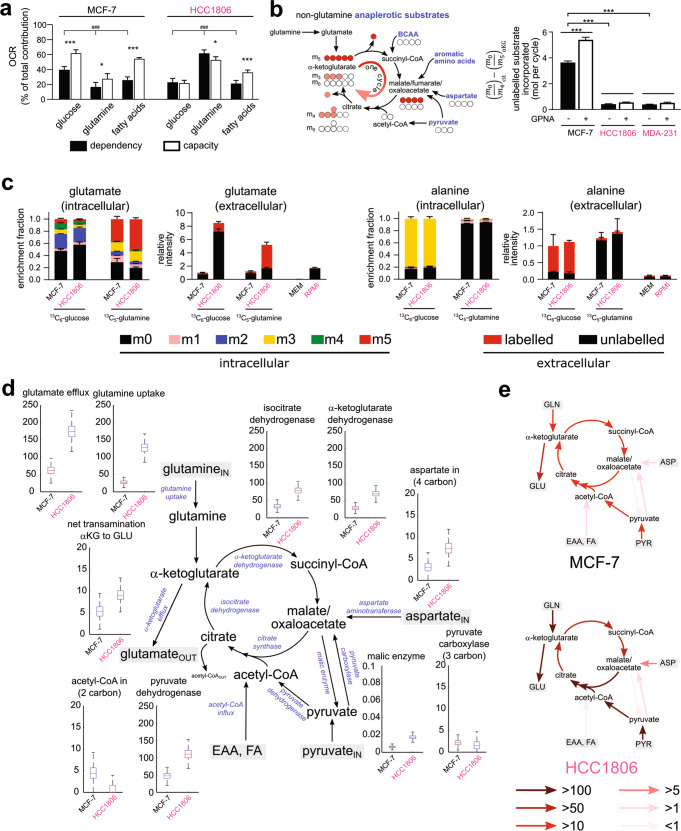
TNBC cells possess a rigid metabolism due to tight coupling of glucose and glutamine catabolic pathways. **a** Oxygen consumption rates (OCR) by Seahorse Mito Fuel Flex performed on MCF-7 and HCC1806 cells showing varying dependency and capacity for glucose, glutamine and fatty acids as oxidative fuels (*n* = 5). *P* values calculated by one-way ANOVA: ###*P* < 0.001; by two-tailed Student’s *t* test: *P < 0.05, ***P < 0.001. **b** The difference in unlabelled:labelled ratios between αKG and citrate show the dilution of ^13^C label from glutamine by anaplerotic influx of non-glutamine carbon sources (*n* = 3). Data from Fig. 1f. *P* values calculated by two-tailed Student’s *t* test: ***P < 0.001. **c** Enrichment fractions of glutamate and alanine from parallel ^13^C-glucose and ^13^C-glutamine tracing experiments (*n* = 4). **d** Steady-state ^13^C flux analysis performed using data from the parallel tracing experiments, showing elevated TCA cycle fluxes in TNBC cells flux distributions generated using a Monte-Carlo resampling technique (800 iterations) are shown as box plots. **e** Pathway heat map summarising flux results. See also Supplementary Fig. S4.

Broadly looking at oxidative fuel preference, it was clear HCC1806 cells prioritised glutamine over glucose and fatty acids, with glutamine contributing to approximately 60% of oxygen consumption rate (OCR) (Fig. 4a). By comparison, MCF-7 cells showed a more distributed preference, biased slightly towards glucose (~40%) compared to fatty acids (~25%) and glutamine (<20%). Importantly, HCC1806 cell fuel usage was inflexible, as they were unable to increase OCR respiration of glutamine or glucose when the other two counterparts were inhibited, with fatty acid being the exception (Fig. 4a). By contrast, MCF-7 cells had a much greater fuel flexibility for all three sources (Fig. 4a). In other words, TNBC cells’ inability to manoeuvre to alternative fuel source(s) suggests glucose and glutamine respiration are tightly coupled—both substrates are metabolised concurrently. The OCR:ECAR ratios, which quantify the contribution of oxidative phosphorylation (OXPHOS) relative to extracellular acidification rate (ECAR) (aerobic glycolysis) [30], were lower for all substrate combinations tested on HCC1806 cells when compared to MCF-7 cells (Supplementary Fig. S4a). We speculate TCA cycle fluxes in HCC1806 cells serve less to oxidise fuel sources, but more to interconvert metabolites.

Next, we tested whether pyruvate anaplerosis can compensate for glutamine anaplerosis. We traced ^13^C-glutamine into the TCA cycle when GPNA reduced glutamine uptake (Supplementary Fig. S4b). GPNA reduced ^13^C-enrichments but drug effects appeared subdued (Supplementary Fig. S4c). Namely by unsupervised clustering of fractional enrichments clear differences between subtypes still prevailed; GPNA did not change glutamine utilisation in TNBC cells to become more like Luminal A. However, determining the extent m_5_ αKG isotopologue was diluted by unlabelled (m_0_) substrates for each cycle αKG traverses around the TCA cycle (i.e. converted into m_4_ citrate), it was clear that GPNA increased anaplerotic influx of non-glutamine substrates in MCF-7 cells (Fig. 4b, Supplementary Fig. S4b). At ~3.6 mol per cycle, MCF-7 cells clearly assimilated more non-glutamine substrates between these two nodes compared to TNBC cells (<0.44 mol per cycle; Fig. 4b). This ratio increased to 5.4 mol per cycle with GPNA treatment in MCF-7 cells, whereas for TNBC cells the ratios remained low with or without treatment (<0.6 mol per cycle). GPNA also increased slightly the extent glutamine carbon is oxidised to CO_2_ in MCF-7 cells (Supplementary Fig. S4d), without altering uptake profiles (Supplementary Fig. S4e). Taken together, we established that MCF-7 cells possess a higher baseline and a greater flexibility and capacity to assimilate non-glutamine substrates (i.e. glucose) when glutamine is limiting, whereas TNBC cells are lacking alternative anaplerotic pathways and rigidly draw glutamine into the TCA cycle even when glutamine is limiting.

We carried out parallel tracing experiments using ^13^C_6_-glucose and ^13^C_5_-glutamine to triangulate TCA cycle fluxes in MCF-7 and HCC1806 cells. From ^13^C-enrichment patterns alone, it was immediately clear that the large amounts of glutamate exported by HCC1806 cells were derived from glucose carbon, not just glutamine (Fig. 4c). Importantly, the dominant m_2_ fraction in glutamate showed single-pass glutaminolysis sponged up glucose-derived acetyl-CoA before overflowing back out. Furthermore, both cell lines generated excess alanine that was predominantly glucose-derived, thus not only confirming the role of NEAA synthesis in producing surplus αKG, but also suggesting single-pass glutaminolysis relies on excess pyruvate from aerobic glycolysis.

To accentuate the dependencies of glucose metabolism on glutamine, we cultured MCF-7 and HCC1806 cells in decreasing starting media glutamine from nominal 2 mM to 0 mM, and traced the fates of ^13^C_6_-glucose. We observed a dose-dependent increase of glucose-derived TCA cycle metabolites with increasing glutamine in HCC1806, with a significant increase in succinate, fumarate and malate, compared to no significant change in MCF-7 glycolytic metabolites (Supplementary Fig. S4g). These data suggest that HCC1806 cells rely on glutamine flux to increase incorporation of glucose carbon into the TCA cycle. Strikingly different αKG and glutamate ^13^C-enrichment patterns were observed for MCF-7 and HCC1806 cells, further suggesting glucose cannot compensate for glutamine deprivation in HCC1806 cells (Supplementary Fig. S4h). There was a concomitant loss of both unlabelled (m0) and labelled (m1-m5) αKG and glutamate in HCC1806 cells as glutamine availability decreased, with glutamate levels only decreasing in MCF-7 at sub-physiological glutamine levels (0.2 or 0 mM). The loss of transamination conjugates glutamate and oxoglutarate explains the deficit in alanine export at 6 h, reiterating the notion that NEAA synthesis is a key component of single-pass glutaminolysis. These data complement previous findings where TNBC subtypes that are sensitive to glucose uptake inhibition (e.g. HCC1806 and MDA-MB-231) are unable to compensate by increasing glutamine oxidation [31]. Thus it appears most TNBC cells have a rigid stoichiometry that governs how glucose and glutamine carbons converge at the TCA cycle, which manifests as a distinctly tight glucose-glutamine metabolic coupling and the lack of compensation between fuel sources.

We next undertook ^13^C flux modelling to consolidate our parallel tracing data by estimating the best in silico fluxes that reproduce both sets of ^13^C data (Supplementary Fig. S4f, Supplementary Table S2) [32]. Fluxes were scaled by the amount of glutamate exported with the units reported as µM per well (Fig. 4d), since each well has the same number of cells and were cultured for the same duration. Flux analyses revealed that (i) HCC1806 TCA cycle fluxes were double that of MCF-7, (ii) for every two mol of glutamine converted to glutamate, one went through the TCA cycle, (iii) pyruvate carboxylase (PC) flux was small but comparable between cell lines, and (iv) net synthesis of glutamate was predominantly from four-carbon source. We also confirmed malic enzyme flux was negligible. Importantly, we quantitatively showed TNBC cells conducted more single-pass glutaminolysis that concomitantly draws glucose into the TCA cycle as acetyl-CoA (Fig. 4e). Thus, we propose that excessive glutamine uptake is exploited by TNBC cells to increase glucose oxidation.

### TNBC gene expression profile drives single-pass glutaminolysis

**Fig. 5:**
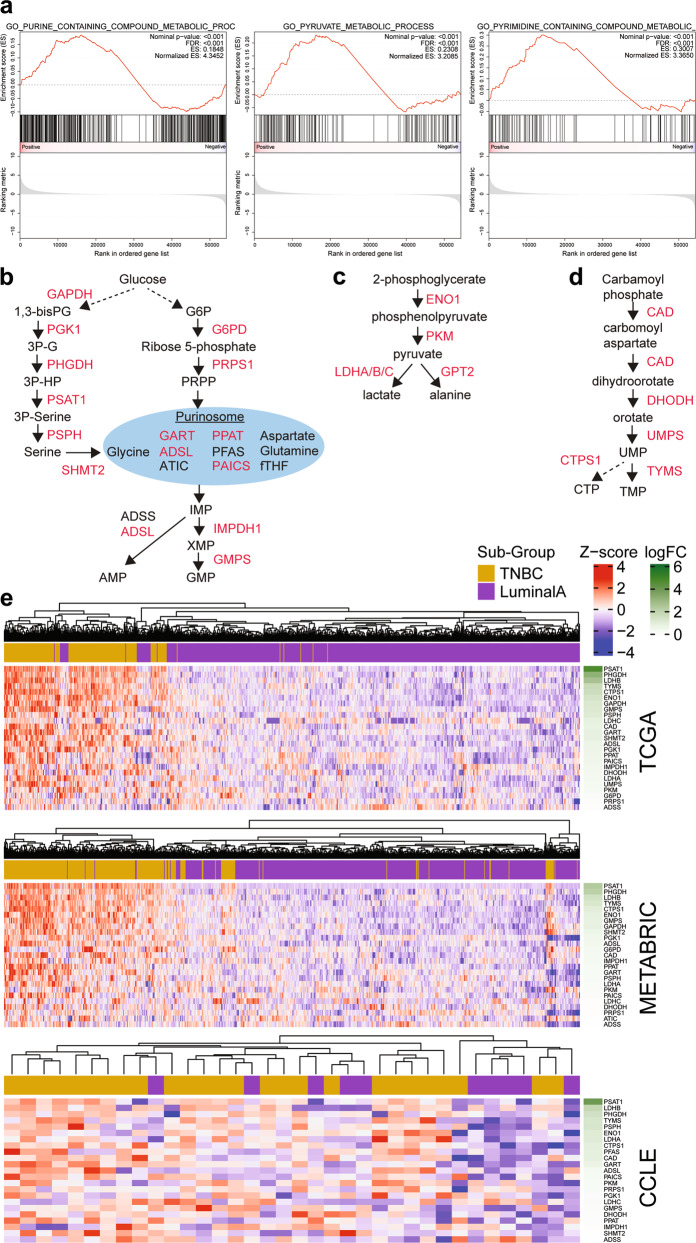
Purine, pyruvate and pyrimidine metabolism enzyme genesets are enriched in TNBC samples. **a** Gene-set Enrichment Analysis (GSEA) was performed comparing mRNA expression data from the TCGA dataset between TNBC/basal-like and Luminal A breast cancer subsets. GSEA plots are shown for GO purine, pyruvate and pyrimidine metabolism genesets that were significantly enriched (FWER < 0.001) in the TCGA dataset. Diagrams illustrating the key genes/enzymes (red = significantly upregulated in TCGA TNBC vs Luminal A) in the purine (**b**), pyruvate (**c**) and pyrimidine (**d**) metabolic genesets and associated metabolites. **e** Heatmaps showing the significantly upregulated genes (TNBC vs Luminal A; *P* < 0.05) from the purine, pyruvate and pyrimidine genesets across TCGA, METABRIC and CCLE datasets. FC: fold-change is indicated in green. See also Supplementary Fig. S5.

Recent data have shown that there are a host of auxiliary enzymes that may have an integral contribution to glutamine metabolism [33], which could “push” and “pull” glutamine into and out of the TCA cycle. To examine this and draw clinical relevance for our findings, we utilised three large mRNA expression datasets from patient tumours (TCGA and METABRIC) and cell lines (CCLE) to examine whether there were metabolic pathways altered in TNBC compared to Luminal A subsets. Gene Set Enrichment Analysis (GSEA) across all 3 datasets revealed 5 significantly enriched Gene Ontology (GO) gene sets (FWER or FDR < 0.001) in the TNBC subset that were relevant to glutamine metabolic pathways (Supplementary Table S3). Hierarchical clustering analysis of the gene expression of these 5 metabolic GO gene sets (total *n* = 930 genes) separated the TNBC and Luminal A subsets across all three datasets (Supplementary Fig. S5 and Supplementary Table S4). These 5 GO gene sets included purine, pyruvate and pyrimidine metabolism (Fig. 5a and Supplementary Fig. S6a, b). Further analysis of the individual genes within these three gene sets revealed significantly increased expression in TNBC samples of enzymes involved in serine/glycine metabolism, the purinosome, purine biosynthesis and pyrimidine biosynthesis pathways across all 3 datasets (Fig. 5b–e). This upregulation would be predicted to divert glucose carbons through into purines, pyrimidines, lactate and alanine; and, more importantly, to utilise glutamine amide for purine and pyrimidine biosynthesis, and to produce glutamate.

**Fig. 6:**
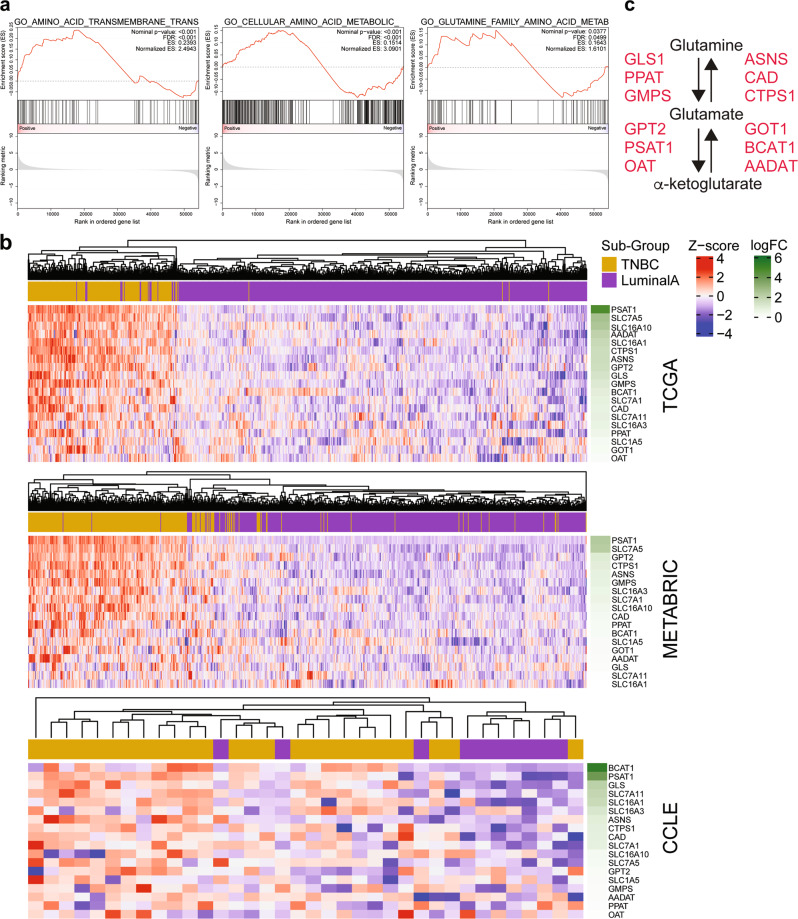
Amino acid transport and metabolism enzyme genesets are enriched in TNBC samples. **a** Gene-set Enrichment Analysis (GSEA) was performed comparing mRNA expression data from the TCGA dataset between TNBC/basal-like and Luminal A breast cancer subsets. GSEA plots are shown for GO amino acid transport and amino acid metabolism genesets (FWER < 0.001) and glutamine metabolism geneset (FDR < 0.05) that were significantly enriched in the TCGA dataset. **b** Heatmaps showing the significantly upregulated genes (TNBC vs Luminal A; P < 0.05) from the amino acid transport, metabolism and glutamine metabolism genesets across TCGA, METABRIC and CCLE datasets. FC: fold-change is indicated in green. **c** Diagram illustrating the key genes/enzymes (red = significantly upregulated in TCGA TNBC vs Luminal A) in these datasets that are involved in glutamine/glutamate/αKG metabolism. See also Supplementary Figs. S6 and S7.

Additional gene sets that were significantly enriched included amino acid transport, amino acid metabolic process, as well as glutamine family amino acid metabolic process (Fig. 6a and Supplementary Fig. S6c, d). Within the amino acid transport gene set, *SLC1A5*/ASCT2 was significantly upregulated as expected (Fig. 6b). Significant upregulation was seen in TNBC for the SLC7 family including the arginine transporter *SLC7A1*/CAT1 and the glutamate/cystine transporter *SLC7A11*/xCT, further supporting our glutamate export data (Supplementary Fig. S1d) and previous publications [13]. In addition, the branched-chain amino acids (BCAA) transporter *SLC7A5*/LAT1 was significantly upregulated in TNBC samples (Fig. 6b), which is important for the supply of BCAAs for protein synthesis and amine for non-essential amino acids (NEAA) synthesis.

**Fig. 7:**
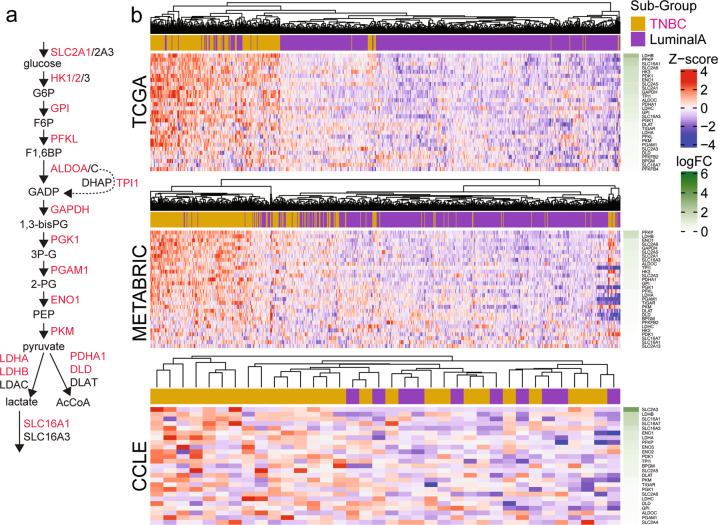
Glycolytic enzymes are enriched in TNBC samples. **a** Diagram illustrating the key glycolytic genes (red = top 20% gene expression in TCGA TNBC). **b** Heatmaps showing the significantly upregulated genes/transporters/enzymes that are involved in glycolysis (TNBC vs Luminal A; *P* < 0.05) across TCGA, METABRIC and CCLE datasets. FC: fold-change is indicated in green.

The amino acid metabolic process and glutamine family gene sets had significantly upregulated genes involved directly with metabolism of glutamine, glutamate and αKG (Fig. 6b, c) [34–37]. These enzymes are critical in converting glutamine to glutamate (with donation of amide groups for purine, pyrimidine and asparagine biosynthesis), and glutamate to and from αKG (Fig. 6c). Among pathways that consume amide nitrogen, we found asparagine synthesis to be distinctly faster in TNBCs inferred from the speed (half saturation time) to reach maximum enrichment (Supplementary Fig. S7). For amine nitrogen, it is known that NEAA aminotransferases *GPT2*, *GOT1* and *PSAT1* are co-regulated with *GLS* [35, 38]. In addition, the coupling of these glutamine pathways with glucose oxidation is also supported by the gene expression profile of TNBC cells. While there are a number of glucose metabolic genes (Fig. 7a) that are significantly upregulated in TNBC compared to Luminal A in both TCGA and METABRIC cohorts (Fig. 7b), these are supported by multiple highly expressed (top 20% gene rank) glucose metabolic genes that are common to both TNBC cells and the more oxidative Luminal A subset (Supplementary Table S5).

**Fig. 8:**
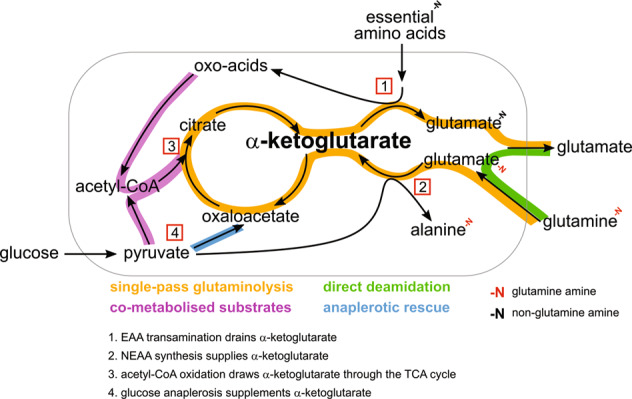
A push and pull pathway model to explain high glutaminolysis flux in TNBC. Single-pass glutaminolysis (orange) in TNBC cells is caused by (1) EAA aminotransferases withdrawing αKG and (2) alanine aminotransferase pushing glutamate into the TCA cycle. (3) αKG is drawn through the TCA cycle by citrate synthase assimilating glucose-derived acetyl-CoA (purple). The single-pass glutaminolysis flux was as large as the net deamidated flux of glutamine (green). (4) MCF-7 cells possessed a higher capacity for pyruvate anaplerosis that supplements αKG (blue), unlike TNBC cells that rely on glutamine to produce αKG.

These data clearly show that TNBC cells have a distinct gene expression profile that is set up for a greater glutamine utilisation compared to Luminal A cells. Metabolic signatures uncovered spanned glutaminolysis, purine/pyrimidine biosynthesis, pyruvate carboxylation and BCAA catabolism, highlighting that glutamine addiction extends beyond glutaminase alone [33, 39, 40]. This concerted upregulation of enzymes and pathways that consume glutamine nitrogen for purines and pyrimidines would “push” glutamine carbon into the TCA cycle, while the aminotransferases that consume αKG would then “pull” those glutamine carbons back out of the TCA cycle (Fig. 8). At a minimum, both patient and cell line expression data substantiate single-pass glutaminolysis as a novel pathway of potential clinical relevance in TNBC.

## Discussion

Despite glutamine being TNBC cells preferred fuel source, it is largely wasted as unoxidised glutamate. We resolve this conundrum by metabolic tracing, showing glutamine carbon indeed flows through the TCA cycle to boost fluxes, but itself achieves no net oxidation since it is effluxed as glutamate. Instead, this TCA cycle configuration promotes oxidation of glucose-derived acetyl-CoA for energy. We speculate single-pass glutaminolysis exploits nutrient gradients to sustain high TCA cycle fluxes and to raise metabolite levels, which fuel the growth of TNBC cells. This non-canonical glutamine-to-glutamate overflow resembles lactate overproduction, which serves to decouple high glycolytic fluxes from the TCA cycle [41].

Our data revealed the coupling of four seemingly autonomous processes: (i) glutaminase, (ii) alanine and aspartate (NEAA) aminotransferases, (iii) acetyl-CoA oxidation, and (iv) EAA aminotransferases, to cause a higher glutamine carbon thoroughfare in the TCA cycle of TNBC cells. NEAA aminotransferases “push” glutamine carbon into the TCA cycle by expending glucose-derived pyruvate. At the same time, EAA breakdown “pulls” glutamine carbon one round through the TCA cycle by EAA aminotransferases withdrawing αKG and by citrate synthase assimilating acetyl-CoA derived from glucose (Fig. 8). Hence the glutamine-to-glutamate overflow is not simply an overactive glutaminase but rather a coordination of key mitochondrial metabolic pathways. An example of this is the activity of GPT2, through which glycolysis can promote glutamine anaplerosis by supplying copious pyruvate [38].

Our findings shed new light on glutamine addiction and dependency. TNBC cells rely on generally abundant glutamine to maintain high TCA cycle fluxes and metabolite levels, which glucose alone cannot achieve. Specifically, we showed glutamine availability elevated glucose oxidation and countered depletion of αKG by EAA breakdown. MCF-7 cells operate TCA cycle at lower fluxes, and perhaps this allows glucose and glutamine to compensate for one another. It is known that an active PC flux confers glutamine independence by drawing glucose into the TCA cycle, thus supplementing αKG when glutamine is limiting [42, 43]. Our work extends this, showing that a responsive PC enzyme in MCF-7 cells can alter the stoichiometric ratio of glucose and glutamine entering the TCA cycle, at a capacity high enough to compensate for loss of glutamine anaplerosis. MCF-7 cells potentially have two pathways to make up loss of αKG “catalyst”: (i) a latent PC enzyme or (ii) by liberating amine as ammonia to freely regenerate αKG via high expression of glutamate dehydrogenase [36]. The fact that cell permeable αKG has been shown to restore TCA cycle metabolites and rescue proliferation highlights the importance for cells to exist in a αKG replete state [15, 18, 35, 44, 45]. The role of αKG in salvaging amine may also be important, as nitrogen re-assimilation can accelerate the growth of breast cancer cells [46]. Producing excess glutamate is hardwired at the gene expression level, and is likely a by-product of prioritising synthesis of NEAA, nucleotides and exchange for critical substrates as required (e.g. leucine and cystine) [13, 20, 21, 47, 48]. Importantly, the prioritised nucleotide synthesis, which relies heavily on glutamine-donated amide groups, is also a hardwired therapeutic vulnerability in TNBC cells [49], which can be targeted (brequinar) in combination with CB839 glutaminase inhibition [50]. Our data support these recent observations, and provide additional therapeutic targets in these metabolic pathways.

As breast cancer metabolism is highly heterogenous, a key limitation of our study is we have generalised the results from four cell lines to explain glutamine addiction using single-pass glutaminolysis. More tracing experiments across multiple cell lines are needed to verify this process, and potentially extend this to other glutamine-addicted cancer subtypes. Nevertheless, the three large mRNA expression datasets confirmed the TNBC subtype has a metabolically programmed landscape to support single-pass glutaminolysis, which may provide new therapeutic opportunities. Indeed, the therapeutic utility of a number of these metabolic targets has been assessed using in vivo TNBC models, including ASCT2 glutamine uptake [10, 14], xCT glutamate/cystine transport [13], glutaminase [15, 50] and pyrimidine metabolism [49, 50]. Single-pass glutaminolysis adds to the mechanism explaining the constraints that facilitate targeting TNBC cancer cells’ reliance on glutamine metabolic processes.

It is a departure from convention to claim that glutamine promotes glucose oxidation and is not a genuine oxidative fuel. However, here we have provided a clearer understanding of excessive glutaminolysis in breast cancer cells. If single-pass glutaminolysis is prevalent among glutamine-addicted tumours, this work will provide new ways to predict patients’ responsiveness to drugs inhibiting glutamine pathways.

## Materials & methods

### Cell culture

Human breast cancer cell lines MCF-7, T47D, HCC1806, and MDA-MB-231 cell lines were purchased from American Type Culture Collection. Cell lines are stored as authenticated low-passage stocks, and have been confirmed by short tandem repeat (STR) fingerprinting (CellBank Australia). MCF-7 cells were grown in MEM (Life Technologies), while T47D, HCC1806 and MDA-MB-231 cells were grown in RPMI-1640 (Life Technologies). Media contained 10% (v/v) foetal bovine serum (FBS; HyClone), 2 mM L-glutamine (Life Technologies), 1 mM Na pyruvate (Life Technologies) and penicillin-streptomycin solution (Sigma-Aldrich, Australia). Cells were maintained at 37 °C in a fully humidified atmosphere containing 5% CO_2_, with monthly testing to ensure cells were mycoplasma-free.

### In vitro ^15^N and ^13^C labelling experiments

Cells were plated in triplicate at a density of 7 × 10^5^ cells/well in 6-well plates in normal growth media and allowed to adhere for 6–8 h. Growth media was then removed and the cell monolayer was washed once with PBS. Media was then replaced with 1 mL of glutamine-free MEM (MCF-7) or RPMI 1640 (HCC1806, MDA-MB-231) containing 10% dialysed FBS, 1 mM sodium pyruvate, and the required labelled substrate and inhibitor. The glutamine substrates used were L-[^15^N-amide]-glutamine, L-[^15^N-amine]-glutamine and L-[^13^C_5_(U)]-glutamine at 2 mM. ^13^C_6_-glucose was at 11 mM in DMEM glucose-free media, or at 50% labelled in glutamine-free RPMI. Both ^13^C_6_-leucine and ^15^N-leucine were at 0.38 mM to match unlabelled leucine already present in media. The duration of exposure to tracer substrate depends on the experiment and is separately indicated. Cell cultures were replicated on different days, with the number of replicates ranging from 3 to 9 to determine group variance rather than to achieve statistical significance.

### Statistical analyses

All analyses were performed using GraphPad Prism 8 software. Tests performed for unpaired groups include one-tailed or two-tailed Student’s *t* test, one-way or two-way ANOVA, Dunnett’s or Mann–Whitney, as well as number of replicates, are indicated in figure legends. Error bars in figures represent standard deviations. For metabolite data from cell cultures sampled at pseudo-steady state, we expect similar variance between groups.

## Supplementary information

Supplementary Figures

Table S1

Table S2

Table S3

Table S4

Table S5

Supplementary Methods

## Data Availability

Source data are provided with this paper. All codes and scripts used for association studies are available on request.

## References

[1] Vettore L, Westbrook RL, Tennant DA (2020). New aspects of amino acid metabolism in cancer. Br J Cancer.

[2] Martinez-Reyes I, Chandel NS (2020). Mitochondrial TCA cycle metabolites control physiology and disease. Nat Commun.

[3] DeBerardinis RJ, Mancuso A, Daikhin E, Nissim I, Yudkoff M, Wehrli S (2007). Beyond aerobic glycolysis: transformed cells can engage in glutamine metabolism that exceeds the requirement for protein and nucleotide synthesis. Proc Natl Acad Sci USA.

[4] Fan J, Kamphorst JJ, Mathew R, Chung MK, White E, Shlomi T (2013). Glutamine-driven oxidative phosphorylation is a major ATP source in transformed mammalian cells in both normoxia and hypoxia. Mol Syst Biol.

[5] Hosios AM, Hecht VC, Danai LV, Johnson MO, Rathmell JC, Steinhauser ML (2016). Amino acids rather than glucose account for the majority of cell mass in proliferating mammalian cells. Dev Cell.

[6] Le A, Lane AN, Hamaker M, Bose S, Gouw A, Barbi J (2012). Glucose-independent glutamine metabolism via TCA cycling for proliferation and survival in B cells. Cell Metab.

[7] Wise DR, DeBerardinis RJ, Mancuso A, Sayed N, Zhang XY, Pfeiffer HK (2008). Myc regulates a transcriptional program that stimulates mitochondrial glutaminolysis and leads to glutamine addiction. Proc Natl Acad Sci USA.

[8] Brunk E, Sahoo S, Zielinski DC, Altunkaya A, Drager A, Mih N (2018). Recon3D enables a three-dimensional view of gene variation in human metabolism. Nat Biotechnol.

[9] Jain M, Nilsson R, Sharma S, Madhusudhan N, Kitami T, Souza AL (2012). Metabolite profiling identifies a key role for glycine in rapid cancer cell proliferation. Science.

[10] Jeon YJ, Khelifa S, Ratnikov B, Scott DA, Feng Y, Parisi F (2015). Regulation of glutamine carrier proteins by RNF5 determines breast cancer response to ER stress-inducing chemotherapies. Cancer cell.

[11] Kim S, Jung WH, Koo JS (2013). The expression of glutamine-metabolism-related proteins in breast phyllodes tumors. Tumour Biol J Int Soc Oncodev Biol Med.

[12] Kung HN, Marks JR, Chi JT (2011). Glutamine synthetase is a genetic determinant of cell type-specific glutamine independence in breast epithelia. PLoS Genet.

[13] Timmerman LA, Holton T, Yuneva M, Louie RJ, Padro M, Daemen A (2013). Glutamine sensitivity analysis identifies the xCT antiporter as a common triple-negative breast tumor therapeutic target. Cancer Cell.

[14] van Geldermalsen M, Wang Q, Nagarajah R, Marshall AD, Thoeng A, Gao D (2016). ASCT2/SLC1A5 controls glutamine uptake and tumour growth in triple-negative basal-like breast cancer. Oncogene.

[15] Gross MI, Demo SD, Dennison JB, Chen L, Chernov-Rogan T, Goyal B (2014). Antitumor activity of the glutaminase inhibitor CB-839 in triple-negative breast cancer. Mol Cancer Therapeutics.

[16] Tardito S, Oudin A, Ahmed SU, Fack F, Keunen O, Zheng L (2015). Glutamine synthetase activity fuels nucleotide biosynthesis and supports growth of glutamine-restricted glioblastoma. Nat Cell Biol.

[17] Reis LMD, Adamoski D, Ornitz Oliveira Souza R, Rodrigues Ascencao CF, Sousa de Oliveira KR, Correa-da-Silva F (2019). Dual inhibition of glutaminase and carnitine palmitoyltransferase decreases growth and migration of glutaminase inhibition-resistant triple-negative breast cancer cells. J Biol Chem.

[18] Lampa M, Arlt H, He T, Ospina B, Reeves J, Zhang B (2017). Glutaminase is essential for the growth of triple-negative breast cancer cells with a deregulated glutamine metabolism pathway and its suppression synergizes with mTOR inhibition. PLoS One.

[19] Li X, Hui S, Mirek ET, Jonsson WO, Anthony TG, Lee WD (2022). Circulating metabolite homeostasis achieved through mass action. Nat Metab.

[20] Nicklin P, Bergman P, Zhang B, Triantafellow E, Wang H, Nyfeler B (2009). Bidirectional transport of amino acids regulates mTOR and autophagy. Cell.

[21] Shin CS, Mishra P, Watrous JD, Carelli V, D’Aurelio M, Jain M (2017). The glutamate/cystine xCT antiporter antagonizes glutamine metabolism and reduces nutrient flexibility. Nat Commun.

[22] Ahn WS, Antoniewicz MR (2013). Parallel labeling experiments with [1,2-(13)C]glucose and [U-(13)C] glutamine provide new insights into CHO cell metabolism. Metab Eng.

[23] Li H, Ning S, Ghandi M, Kryukov GV, Gopal S, Deik A (2019). The landscape of cancer cell line metabolism. Nat Med.

[24] Budczies J, Pfitzner BM, Gyorffy B, Winzer KJ, Radke C, Dietel M (2015). Glutamate enrichment as new diagnostic opportunity in breast cancer. Int J Cancer.

[25] Cao MD, Lamichhane S, Lundgren S, Bofin A, Fjosne H, Giskeodegard GF (2014). Metabolic characterization of triple negative breast cancer. BMC Cancer.

[26] Vande Voorde J, Ackermann T, Pfetzer N, Sumpton D, Mackay G, Kalna G (2019). Improving the metabolic fidelity of cancer models with a physiological cell culture medium. Sci Adv.

[27] Centenera MM, Raj GV, Knudsen KE, Tilley WD, Butler LM (2013). Ex vivo culture of human prostate tissue and drug development. Nat Rev Urol.

[28] Cantor JR, Abu-Remaileh M, Kanarek N, Freinkman E, Gao X, Louissaint A (2017). Physiologic medium rewires cellular metabolism and reveals uric acid as an endogenous inhibitor of UMP synthase. Cell.

[29] Davidson SM, Papagiannakopoulos T, Olenchock BA, Heyman JE, Keibler MA, Luengo A (2016). Environment impacts the metabolic dependencies of ras-driven non-small cell lung cancer. Cell Metab.

[30] Zhang J, Nuebel E, Wisidagama DR, Setoguchi K, Hong JS, Van Horn CM (2012). Measuring energy metabolism in cultured cells, including human pluripotent stem cells and differentiated cells. Nat Protoc.

[31] Wu Q, Ba-Alawi W, Deblois G, Cruickshank J, Duan S, Lima-Fernandes E (2020). GLUT1 inhibition blocks growth of RB1-positive triple negative breast cancer. Nat Commun.

[32] Quek LE, Nielsen LK (2014). Steady-state (1)(3)C fluxomics using OpenFLUX. Methods Mol Biol.

[33] Méndez-Lucas A, Lin W, Driscoll PC, Legrave N, Novellasdemunt L, Xie C (2020). Identifying strategies to target the metabolic flexibility of tumours. Nat Metab.

[34] Possemato R, Marks KM, Shaul YD, Pacold ME, Kim D, Birsoy K (2011). Functional genomics reveal that the serine synthesis pathway is essential in breast cancer. Nature.

[35] Yang CS, Stampouloglou E, Kingston NM, Zhang L, Monti S, Varelas X (2018). Glutamine-utilizing transaminases are a metabolic vulnerability of TAZ/YAP-activated cancer cells. EMBO Rep.

[36] Craze ML, El-Ansari R, Aleskandarany MA, Cheng KW, Alfarsi L, Masisi B (2019). Glutamate dehydrogenase (GLUD1) expression in breast cancer. Breast Cancer Res Treat.

[37] Knott SRV, Wagenblast E, Khan S, Kim SY, Soto M, Wagner M (2018). Asparagine bioavailability governs metastasis in a model of breast cancer. Nature.

[38] Smith B, Schafer XL, Ambeskovic A, Spencer CM, Land H, Munger J (2016). Addiction to coupling of the warburg effect with glutamine catabolism in cancer cells. Cell Rep.

[39] Lanning NJ, Castle JP, Singh SJ, Leon AN, Tovar EA, Sanghera A (2017). Metabolic profiling of triple-negative breast cancer cells reveals metabolic vulnerabilities. Cancer Metab.

[40] Martin SD, McGee SL (2019). A systematic flux analysis approach to identify metabolic vulnerabilities in human breast cancer cell lines. Cancer Metab.

[41] Hui S, Ghergurovich JM, Morscher RJ, Jang C, Teng X, Lu W (2017). Glucose feeds the TCA cycle via circulating lactate. Nature.

[42] Simoes RV, Serganova IS, Kruchevsky N, Leftin A, Shestov AA, Thaler HT (2015). Metabolic plasticity of metastatic breast cancer cells: adaptation to changes in the microenvironment. Neoplasia.

[43] Cheng T, Sudderth J, Yang C, Mullen AR, Jin ES, Mates JM (2011). Pyruvate carboxylase is required for glutamine-independent growth of tumor cells. Proc Natl Acad Sci USA.

[44] Lee P, Malik D, Perkons N, Huangyang P, Khare S, Rhoades S (2020). Targeting glutamine metabolism slows soft tissue sarcoma growth. Nat Commun.

[45] Vatrinet R, Leone G, De Luise M, Girolimetti G, Vidone M, Gasparre G (2017). The alpha-ketoglutarate dehydrogenase complex in cancer metabolic plasticity. Cancer Metab.

[46] Spinelli JB, Yoon H, Ringel AE, Jeanfavre S, Clish CB, Haigis MC (2017). Metabolic recycling of ammonia via glutamate dehydrogenase supports breast cancer biomass. Science.

[47] van Geldermalsen M, Quek LE, Turner N, Freidman N, Pang A, Guan YF (2018). Benzylserine inhibits breast cancer cell growth by disrupting intracellular amino acid homeostasis and triggering amino acid response pathways. BMC Cancer.

[48] Osanai-Sasakawa A, Hosomi K, Sumitomo Y, Takizawa T, Tomura-Suruki S, Imaizumi M (2018). An anti-ASCT2 monoclonal antibody suppresses gastric cancer growth by inducing oxidative stress and antibody dependent cellular toxicity in preclinical models. Am J Cancer Res.

[49] Brown KK, Spinelli JB, Asara JM, Toker A (2017). Adaptive reprogramming of de novo pyrimidine synthesis is a metabolic vulnerability in triple-negative breast cancer. Cancer Discov.

[50] Liao C, Glodowski CR, Fan C, Liu J, Mott KR, Kaushik A (2022). Integrated metabolic profiling and transcriptional analysis reveals therapeutic modalities for targeting rapidly proliferating breast cancers. Cancer Res.

