# Supplementary material for: Glutamine addiction promotes glucose oxidation in triple-negative breast cancer
Source: Oncogene. 2022 Jul 18;41(34):4066–78. doi: 10.1038/s41388-022-02408-5 (PMC9391225; doi:10.1038/s41388-022-02408-5)
Supplement: Supplementary file 1 — Supplementary Figures [file 41388_2022_2408_MOESM1_ESM.pdf]

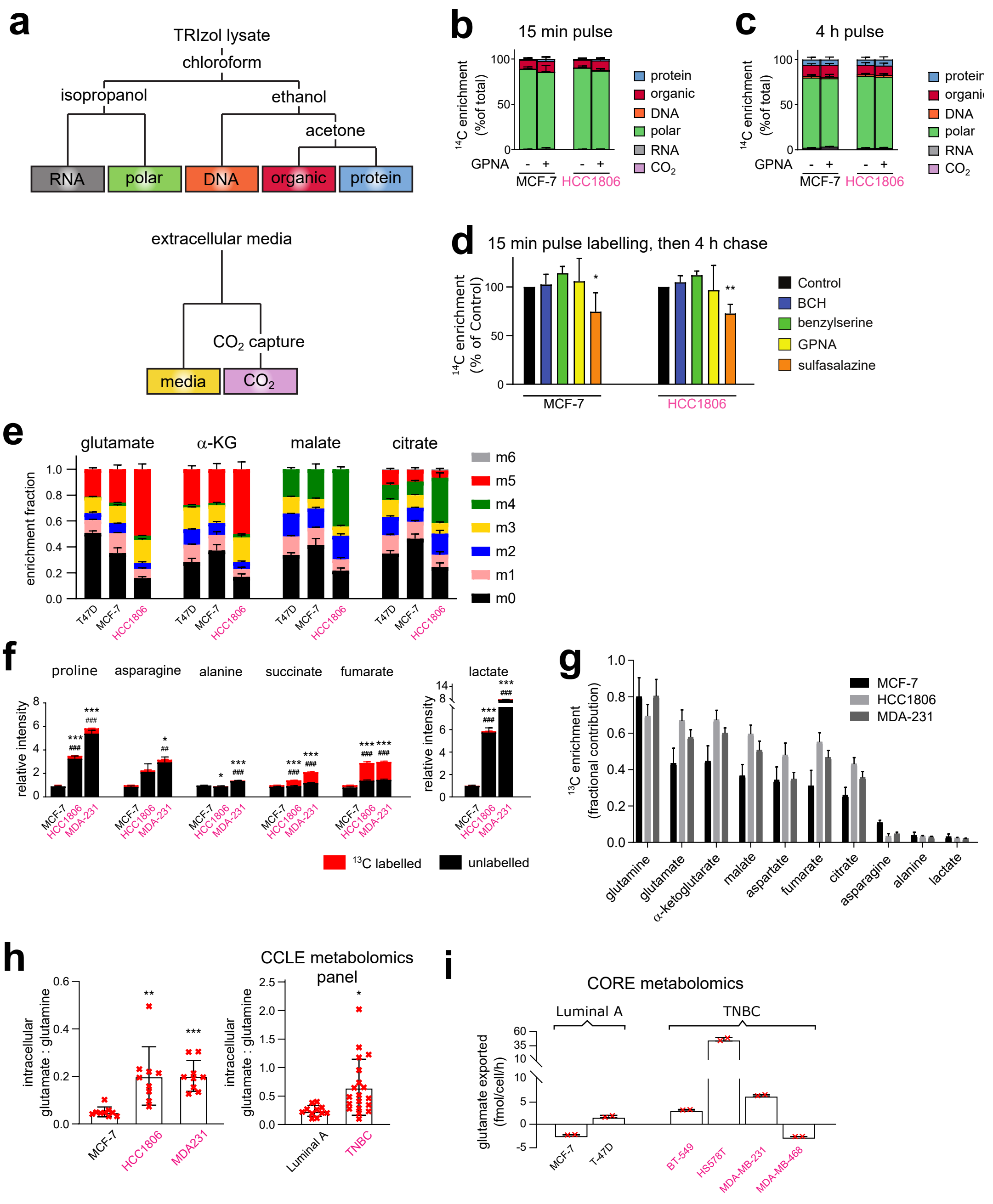

Fig. S1: Glutamine ^{14}C radiolabelling and ^{13}C enrichment showing differences in glutamine utilisation between Luminal A and TNBC cells.

a, Schematic of the fractional separation strategy modified from Hosios *et al*. (2016) used to isolate each of the phases analysed in ^{14}C-glutamine tracing experiments. **b-c**, ^{14}C enrichment data from Fig. 1b expressed as a percentage of total ^{14}C signal detected in each sample (MCF-7 n=3, HCC1806 15 min n=4 and 4 h n=5). **d**, ^{14}C enrichment of the extracellular media from MCF-7 and HCC1806 cells after 15 min pulse labelling with U-$^{14}C_5$-glutamine, followed by 4 h chase with unlabelled media containing 10 mM BCH, 10 mM benzylserine, 1 mM GPNA, 0.5 mM sulfasalazine or vehicle control (n=4). P-values calculated by Dunnett test: *P<0.05, **P<0.01. **e**, $^{13}C_5$-glutamine enrichment data of T47D cells (n=6) compared to MCF-7 and HCC1806 cells (n=4). **f**, Intracellular abundance of ^{13}C-labelled and unlabelled metabolites expressed relative to MCF-7 (n=3). P-values calculated by two-tailed Student's t-test with respect to MCF-7: for total: #P<0.05, ##P<0.01, ###P<0.001, for ^{13}C-labelled: **P<0.01; ***P<0.001. **g**, Cascade plot showing decreasing ^{13}C enrichment fractions across TCA cycle metabolites and amino acids (n=3). Bars toward the left are proximal to exogenous glutamine. **h**, Ratios of glutamate to glutamine (denominator) (n=9) and from Li et al. (2019) (TNBC n=19; Luminal A n =11). P-values calculated by two-tailed Student's t-test with respect to Luminal A/MCF-7: *P<0.05, **P<0.01, ***P<0.001. **i**, Glutamate export rate from CORE data (n=2). Data are represented as mean ± s.d.

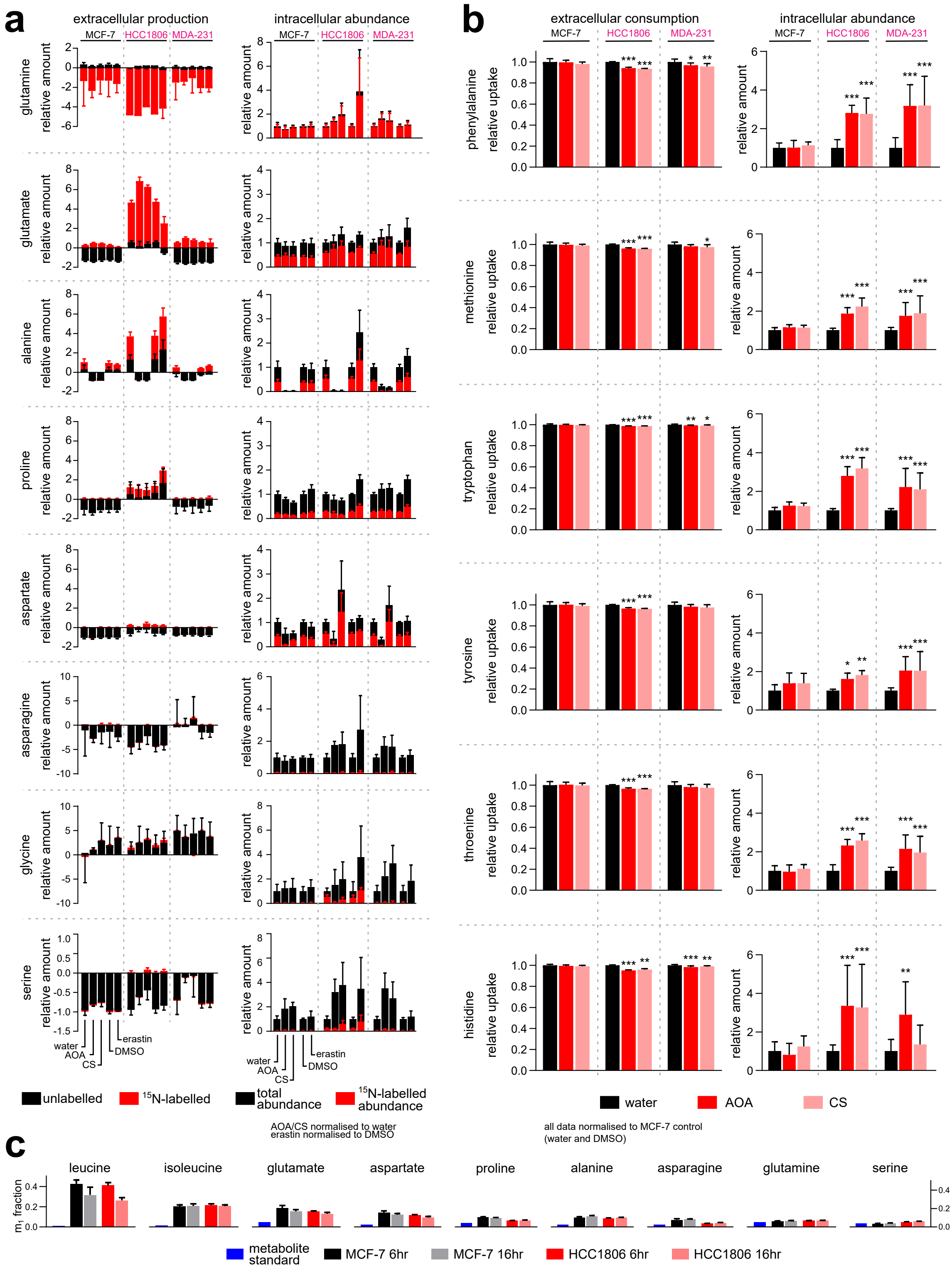

Fig. S2: Inhibitors AOA, CS and erastin reduced amino acid breakdown and altered assimilation of amine nitrogen from ^{15}N-amine glutamine.

a, Extracellular production and intracellular abundance of ^{15}N-labelled and unlabelled metabolites when cells incubated 16 h in ^{15}N-amine-labelled glutamine and were treated with AOA, CS or erastin (n=9). Signal intensities were normalised to water or DMSO as indicated. **b**, Changes to the extracellular consumption and intracellular abundance of EAAs when cells were treated with AOA and CS (n=9). Treatments are shown relative to control (water). P-values were calculated by two-tailed Student's t-test with respect to controls: *P<0.05, **P<0.01, ***P<0.001. **c**, m_1 fraction of intracellular amino acids from cells incubated 6 or 16 h in ^{15}N-leucine (n=4, except HCC1806 16 h n=3). Background (natural enrichment) signals indicated by unlabelled metabolite standards.

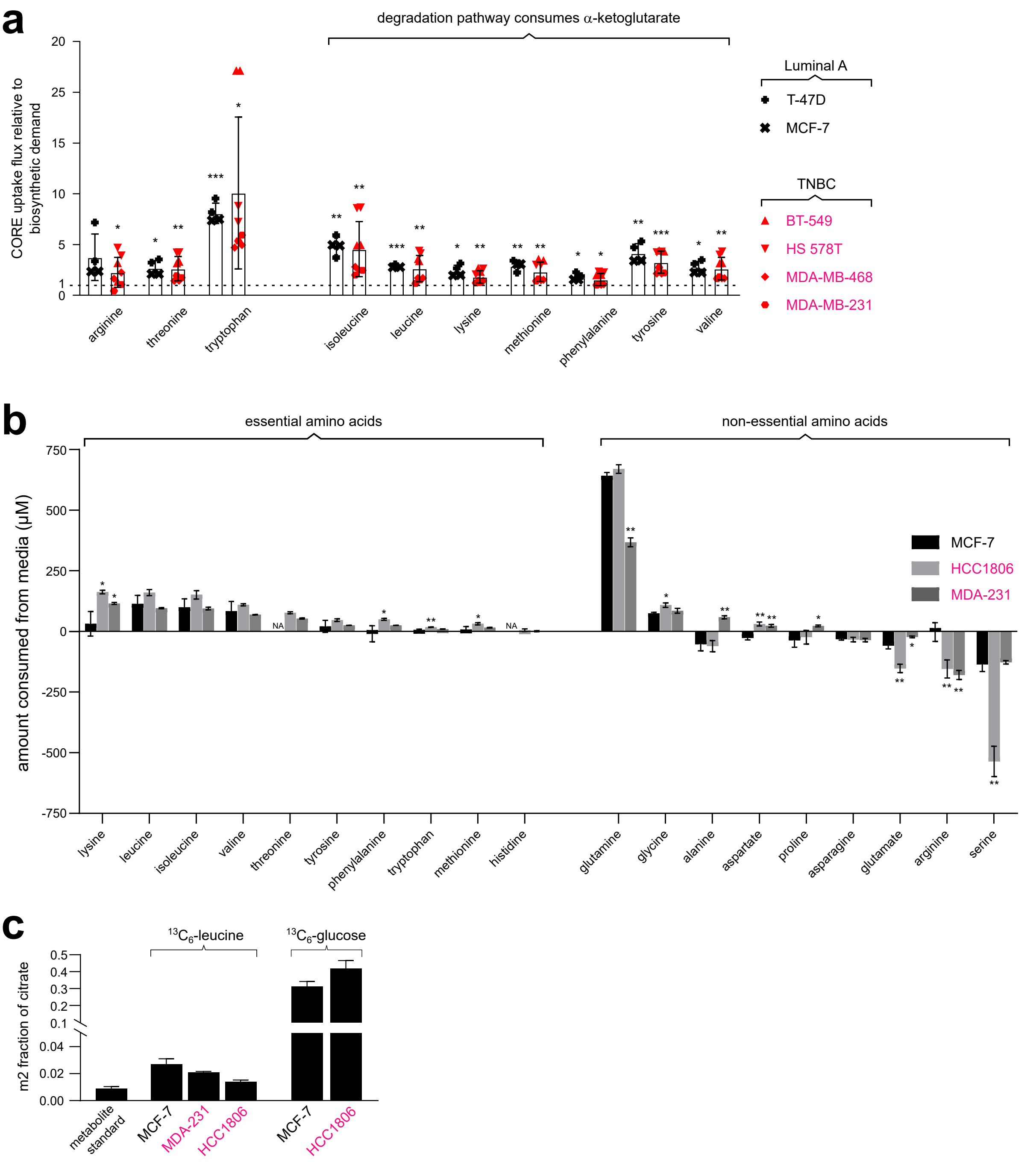

Fig. S3: Breast cancer cells consume more EAAs than required for biosynthesis

a, EAA uptake rates for breast cancer cell lines taken from CORE were compared to biosynthesis rates calculated from the biomass composition of a generic human cell from Recon 3D (n=2). P-values calculated by one-sample t-test for ratio > 1: *P<0.05, **P<0.01. **b**, The amount of essential and non-essential amino acids consumed (positive) and produced (negative) by cells calculated from abundances before and after 16 h incubation (n=3). P-values calculated by two-tailed Student's t-test with respect to MCF-7: *P<0.05, **P<0.01. **c**, m_2 fraction of citrate from cells incubated 16 h in $^{13}C_6$-leucine (n=4). Fractions are compared to citrate label obtained from metabolite standard and from $^{13}C_6$-glucose experiment.

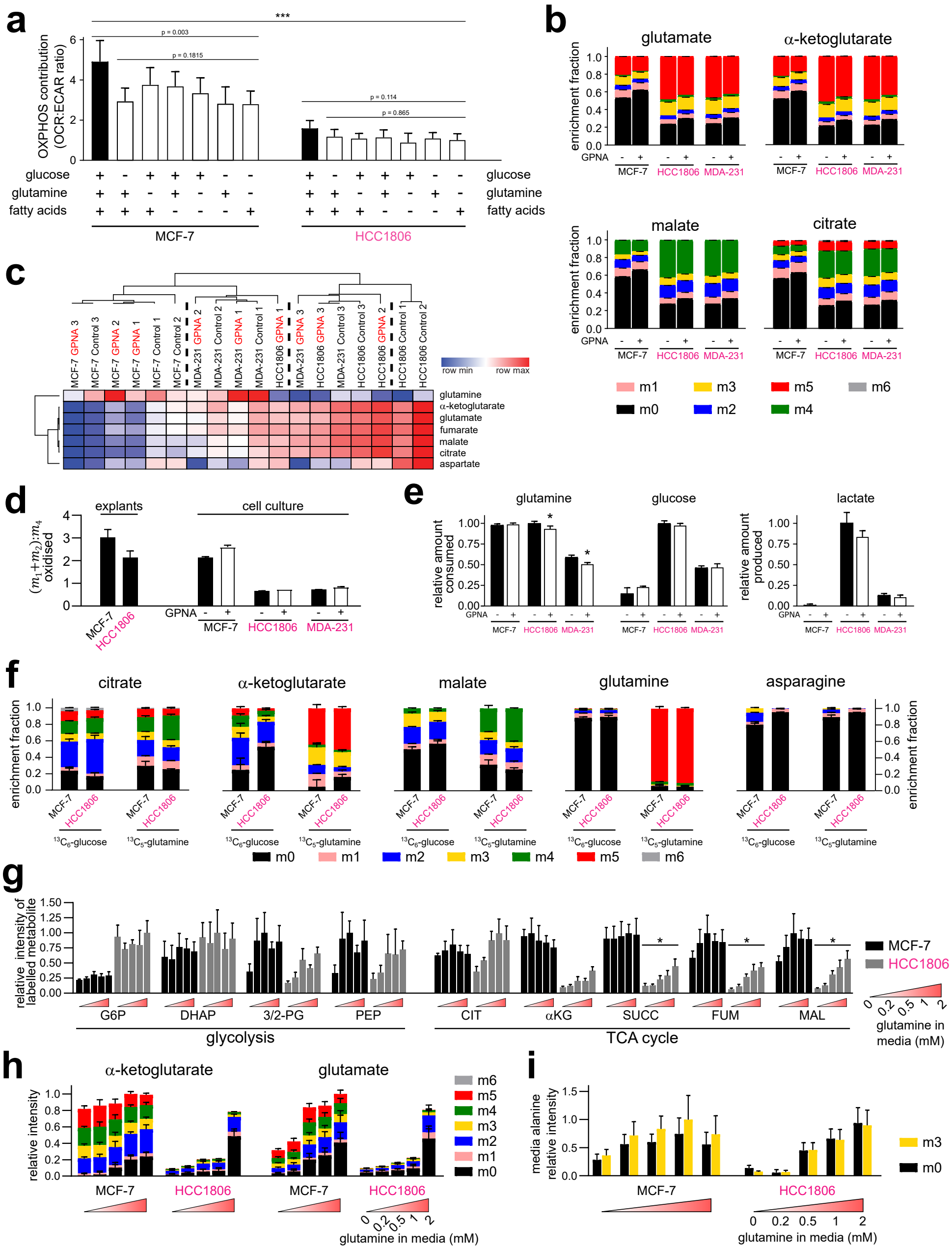

Fig. S4: Additional data from Seahorse Mito Fuel Flex and ^{13}C tracing to support coupling of glucose and glutamine pathways in single-pass glutaminolysis

a, Relative contribution of oxidative phosphorylation (OXPHOS) in MCF-7 and HCC1806 cells, expressed as a ratio of (OCR) to extracellular acidification rate (ECAR), at baseline (black). Minus sign indicates catabolic inhibition of glucose, glutamine, fatty acid or combination thereof (n=5). P-values calculated by two-way ANOVA (cell line factor): ###P<0.001; by one-way ANOVA: displayed. **b**, ^{13}C enrichment patterns of TCA cycle metabolites and glutamate after 14 h incubation in U-$^{13}C_5$-glutamine with or without 1 mM GPNA (n=3). **c**, Unsupervised clustering of ^{13}C-enrichment data in **b**, showing clustering of cell lines and treatments. **d**, Comparison of isotopologue ratios from explants glutamate (Fig. **2d**) and cell culture αKG (**b**), to show proportion of glutamine carbon undergoing further oxidation in response to GPNA (n=3). **e**, Relative amounts of glutamine and glucose consumed and of lactate produced, normalised to HCC1806 without GPNA (n=3). P-values for the effects of GPNA were calculated by two-tailed Student's t-test: *P<0.05. **f**, ^{13}C enrichment patterns of additional metabolites from parallel glucose and glutamine tracing experiments (n=4). **g**, Relative abundance of labelled ($^{13}C_6$-glucose-derived) intracellular metabolites at 24 h, with starting glutamine in media ranged 0 to 2 mM (n=6). Intensities normalised to the maximum within each metabolite group. Note that MCF-7 and HCC1806 cells were cultured in 100% and 56.4% $^{13}C_6$-glucose, respectively, and abundance calculations have accounted for this difference. P-values calculated by two-tailed Spearman's correlation between intensities and glutamine concentrations: *P<0.05. G6P: glucose 6-phosphate, DHAP: dihydroxyacetone phosphate, 3/2-PG: 3-phosphoglycerate and 2-phosphoglycerate, PEP: phosphoenolpyruvate, CIT: citrate, αKG: α-ketoglutarate, SUCC: succinate, FUM: fumarate, MAL: malate. **h**, ^{13}C enrichment patterns of α-ketoglutarate and glutamate at 24 h from $^{13}C_6$-glucose tracing, with starting glutamine in media ranged 0 to 2 mM (n=6). **i**, Media abundance of m_0 and m_3 alanine produced from $^{13}C_6$-glucose at 6 h, with starting glutamine in media ranged 0 to 2 mM (n=6). Data normalised to the maximum time-point average.

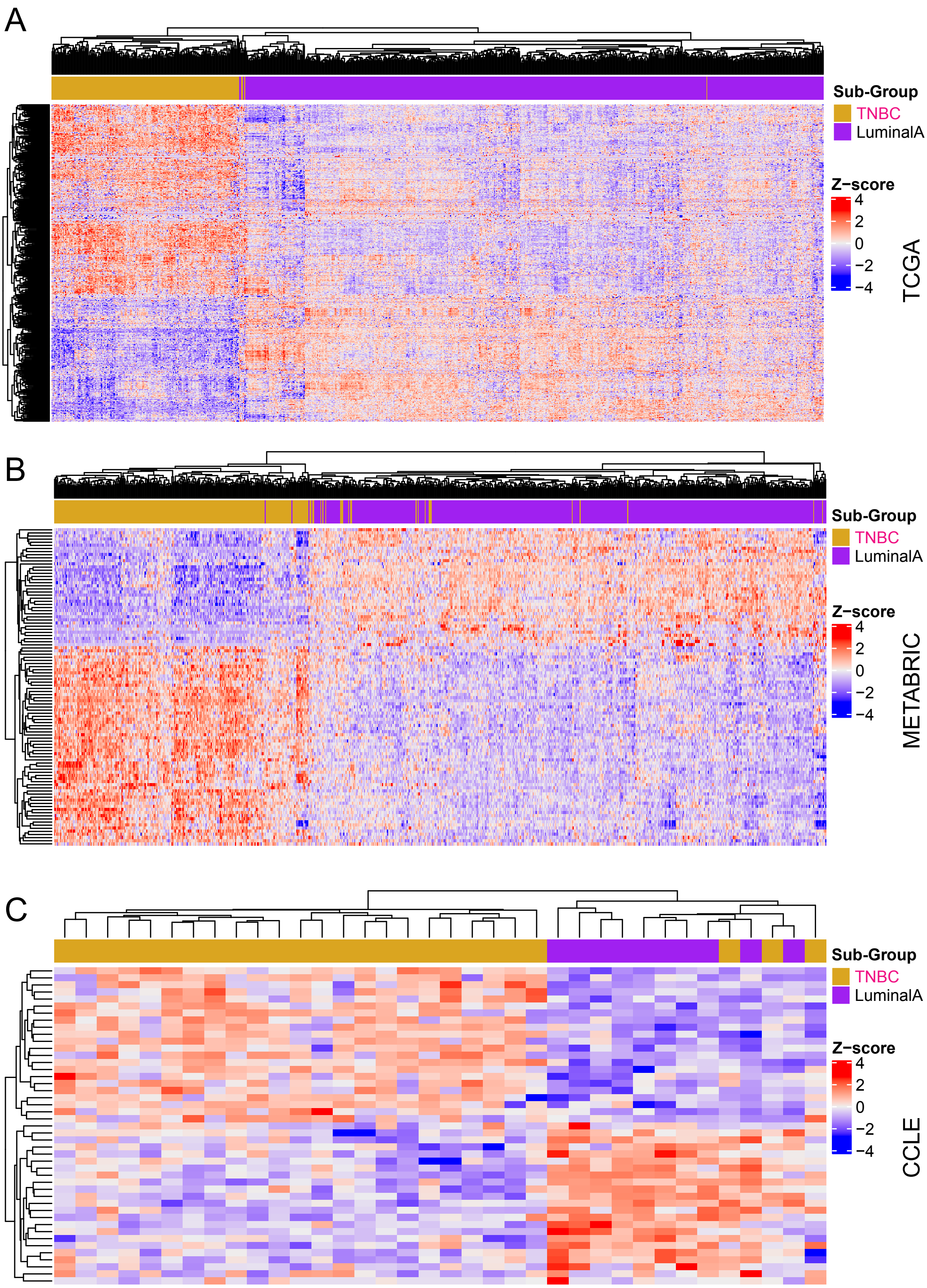

Fig. S5: Heatmaps of significantly altered metabolic genes from GSEA analysis showing clustering of TNBC and Luminal A subtypes.
a,**b**,**c**, Heatmaps showing the significantly altered genes (TNBC vs Luminal A) from the 5 metabolic gene sets from Table S3 using the TCGA (**a**), METABRIC (**b**) and CCLE (**c**) datasets.

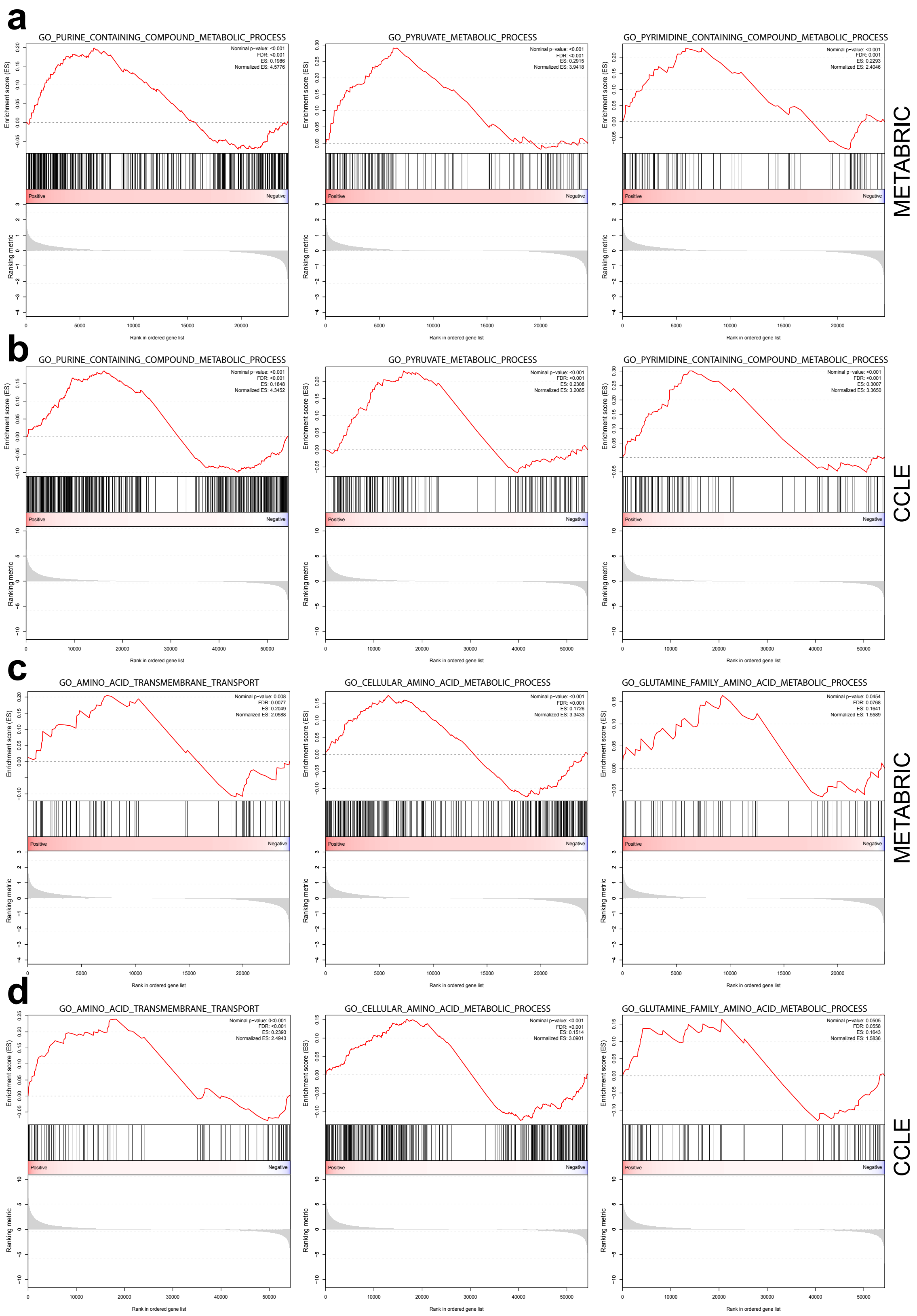

Fig. S6: Gene-set enrichment analysis for METABRIC and CCLE TNBC datasets
a,b, Gene-set Enrichment Analysis (GSEA) was performed comparing mRNA expression data from the TCGA dataset between TNBC/basal-like and Luminal A breast cancer subsets. GSEA plots are shown for GO purine, pyruvate (FWER<0.001) and pyrimidine (FDR<0.001) metabolism gene sets that were significantly enriched in the METABRIC (**a**) and CCLE (**b**) datasets. c,d, GSEA plots are shown for GO amino acid transport and amino acid metabolism gene sets (FWER<0.001) and glutamine metabolism gene set (FDR<0.05) that were significantly enriched in the METABRIC (**c**) and CCLE (**d**) datasets.

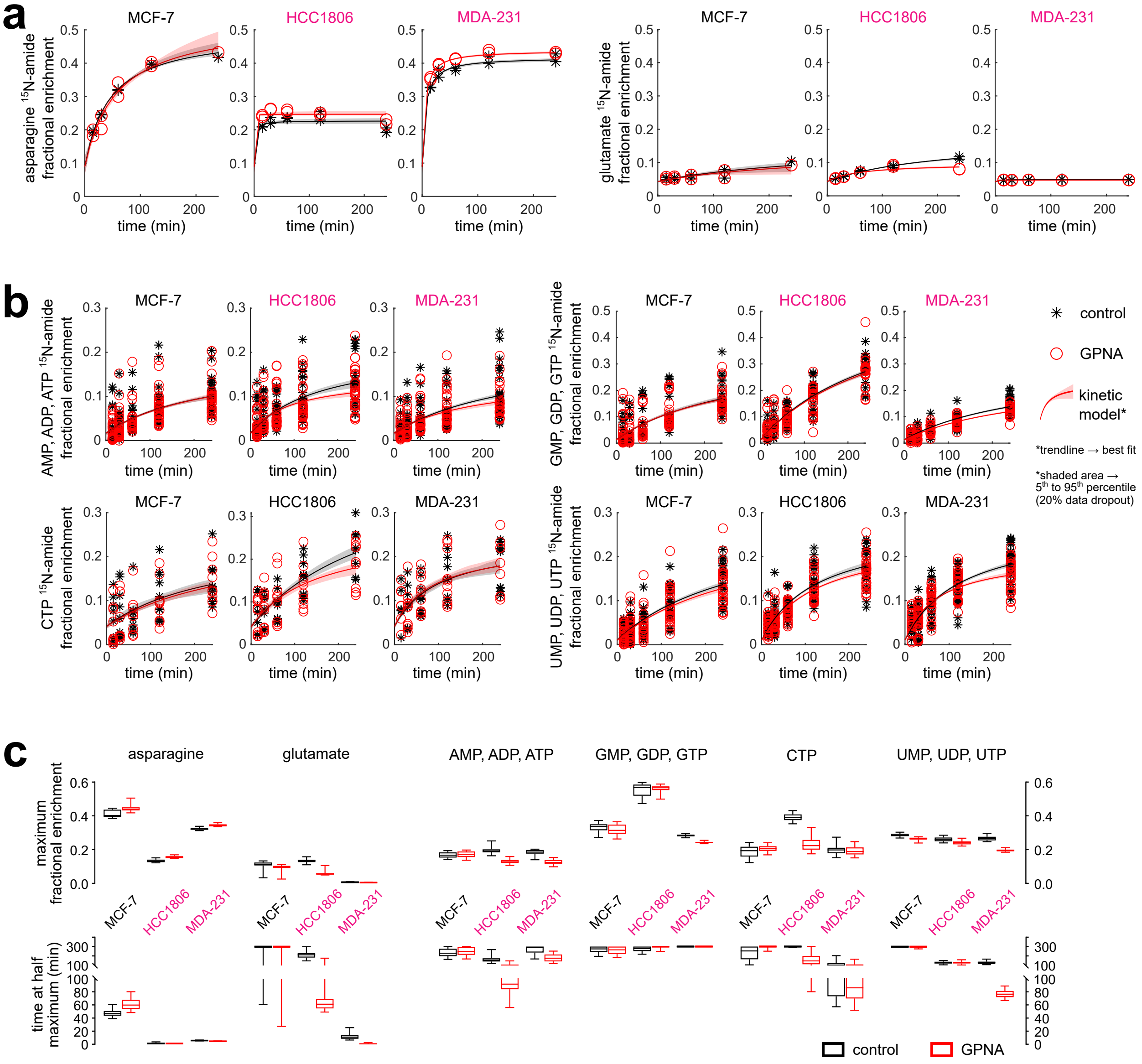

Fig. S7: Assimilation rates of amide nitrogen from ^{15}N-amide-glutamine.

a,**b**, The trajectory of ^{15}N-amide label incorporation derived from glutamine over 4 hours into asparagine and glutamate (n=2) (**a**), and nucleotides (n=4, some with outliers removed) (**b**) in the presence of 1 mM GPNA or vehicle control. An empirical kinetic model based on Michaelis-Menten equation was fitted to the data to derive kinetic parameters shown in **c**. **c**, Kinetic parameters representing rate of enrichment (time to half maximum) and maximum enrichment calculated from data in **a** and **b**. Parameter sensitivity analysis was performed by resampling 80% of the datapoints without replacement, with the data shown as boxplots.
