# Supplementary material for: Glutamine addiction promotes glucose oxidation in triple-negative breast cancer
Source: Oncogene. 2022 Jul 18;41(34):4066–78. doi: 10.1038/s41388-022-02408-5 (PMC9391225; doi:10.1038/s41388-022-02408-5)
Supplement: Supplementary file 2 — Table S1 [file 41388_2022_2408_MOESM2_ESM.pdf]

Table S1: Fractional contributions of 13C to metabolites extracted at steady state from breast cancer cell lines or explanted xenograft tissue. Key: * significantly diff to MCF-7; # significantly different to HCC1806; FC, fractional contribution; SD, standard deviation.

In vitro	**MCF7**		**HCC1806**			**MDA-231**		
	FC (%)	SD		FC (%)	SD		FC (%)	SD
Glutamine	79.9	10.6	*	69.4	6.4	#	80.3	9.3
Glutamate	43.4	8.4	*	66.7	6	*#	57.8	4.1
αKG	44.7	8.4	*	67.3	5.3	*#	60	2.9
Malate	36.4	6.4	*	59.5	5	*#	50.6	5.1
Aspartate	34.2	7.3	*	47.9	6.6	#	34.7	3.8
Fumarate	31	8.5	*	55.1	5.2	*#	46.6	3.9
Citrate	25.8	4.4	*	43	3.6	*#	35.7	3.2
Asparagine	10.7	1.5	*	3.4	1.4	*#	4.7	0.9
Alanine	3.8	1.8	*	3.4	0.4	*#	3	0.3
Pyruvate	4.6	2.3	*	3.3	0.6	*#	2.7	0.4
Lactate	3.1	1.5	*	2.3	0.4	*#	2.1	0.3
Serine	0.9	0.1	*	2.3	0.4	*#	0.9	0.1
Glycine	1	0.1	*	1	0.1	*#	1.1	0.2
Glycerol	1.4	0.2	*	1.1	0.1	*#	1.4	0.2

**GPNA**	**MCF7**		**HCC1806**		**MDA-231**	
	FC (%)	SD	FC (%)	SD	FC (%)	SD
Glutamine	80.96	7.008	67.56	8.83	76.44	13.69
Glutamate	34.6	7.005	59.95	5.162	52.12	2.924
αKG	36.06	7.347	61.16	5.876	54.09	2.907
Malate	29.3	5.507	54.03	5.012	45.11	5.145
Aspartate	26.72	6.753	37.55	4.455	23.12	5.084
Fumarate	23.83	8.558	49.66	4.847	41.38	2.839
Citrate	20.67	3.31	39.77	3.694	32.79	2.701
Asparagine	8.702	0.9993	2.991	1.242	3.83	0.8309
Alanine	2.883	1.001	3.185	0.2655	2.88	0.2402
Pyruvate	3.315	1.228	2.976	0.44	2.379	0.3
Lactate	2.405	0.7643	2.299	0.2904	2.077	0.3226
Serine	0.8919	0.1387	0.9887	0.0636	0.9655	0.07024
Glycine	1.01	0.05938	1.097	0.08415	1.067	0.1479
Glycerol	1.382	0.1975	1.35	0.1478	1.333	0.1427

Explant	**MCF7**		**HCC1806**		
	FC (%)	SD		FC (%)	SD
Glutamine	63.5	24		66.9	8
Glutamate	23.8	10.3		27	1.4
αKG					
Malate	15	4.1		21.2	1.6
Aspartate	10.8	3		19.2	0.9
Fumarate	18.4	4.8		21.5	3.9
Citrate	6.1	0.4	*	10.2	0.7
