# Supplementary material for: Glutamine addiction promotes glucose oxidation in triple-negative breast cancer
Source: Oncogene. 2022 Jul 18;41(34):4066–78. doi: 10.1038/s41388-022-02408-5 (PMC9391225; doi:10.1038/s41388-022-02408-5)
Supplement: Supplementary file 3 — Table S2 [file 41388_2022_2408_MOESM3_ESM.pdf]

Table S2: TCA cycle model and data

MODEL

RxnID	rxnEq	rxnCTrans	rates	rxnType	LB	UB
R01	PYR_lab = PYR	abc = abc		F		
R02	ACCOA_unlab = ACCOA	ab = ab		F		
R03	PYR = ACCOA + CO2	abc = bc + a		F		
R04	MALOAA + ACCOA = CIT	cdef + ab = fedbac		F		
R05	CIT = ACCOA_out + MALOAA	fedbac = ab + cdef		F		
R06	CIT = AKG + CO2	abcdef = abcde + f		F		
R07	AKG + CO2 = CIT	abcde + f = abcdef		F		
R08	AKG = 0.5 SUCC + 0.5 SUCC + CO2	abcde = 0.5 bcde + 0.5 edcb + a		F		
R09	SUCC = MALOAA	abcd = abcd		F		
R10	MALOAA = 0.5 SUCC + 0.5 SUCC	abcd = 0.5 abcd + 0.5 dcba		F		
R11	MALOAA = PYR + CO2	abcd = abc + d		F		
R12	PYR + CO2 = MALOAA	abc + d = abcd		F		
R13	AKG = GLU	abcde = abcde		F		
R14	GLU = AKG	abcde = abcde		F		
R15	GLN = GLU	abcde = abcde		F		
R16	GLN_lab = GLN	abcde = abcde		F		
R17	GLU_unlab = GLU	abcde = abcde		F		
R18	GLU = GLU_out	abcde = abcde		F		
R19	GLU_unlab = GLU_out	abcde = abcde		F		
R20	CO2 = CO2_out	a = a		F		
R21	CO2_unlab = CO2	a = a		F		
R22	MALOAA = ASP	abcd = abcd		F		
R23	ASP = MALOAA	abcd = abcd		F		
R24	ASP = ASP_out	abcd = abcd		F		
R25	ASP_unlab = ASP	abcd = abcd		F		
##	excludedMetabolites					
#	PYR_lab					
#	ACCOA_unlab					
#	ACCOA_out					
#	GLN_lab					
#	GLN_unlab					
#	CO2_out					
#	CO2_unlab					
#	ASP_out					
#	ASP_unlab					
#	GLU_out					
#	GLU_unlab					
##	simulatedMDVs					
#	AKG#11111					
#	MALOAA#1111					
#	ASP_out#1111					
#	GLU#11111					
#	CIT#111111					
#	GLU_out#11111					
##	inputSubstrates					
#	PYR_lab					
#	ACCOA_unlab					
#	GLN_lab					
#	GLN_unlab					
#	GLU_unlab					
#	CO2_unlab					
#	ASP_unlab					

DATA

HCC				MCF7			
glucose experiment		glutamine experiment		glucose experiment		glutamine experiment	
AKG#1111 C5H5O5		AKG#1111 C5H5O5		AKG#1111 C5H5O5		AKG#1111 C5H5O5	
0.53451	0.06524	0.16970	0.02990	0.256	0.140469	0.049	0.085757
0.04765	0.00388	0.06493	0.01717	0.056	0.005515	0.156	0.007878
0.25473	0.03006	0.05398	0.00876	0.332	0.058256	0.108	0.005605
0.06315	0.01505	0.18085	0.02377	0.134	0.040846	0.216	0.051689
0.07263	0.01469	0.02842	0.00094	0.136	0.021718	0.032	0.007584
0.02733	0.00610	0.50212	0.02277	0.086	0.021071	0.439	0.047846
MALOAA#1 C4H5O5		MALOAA#1 C4H5O5		MALOAA#1 C4H5O5		MALOAA#1 C4H5O5	
0.57237	0.03436	0.25900	0.02074	0.503764	0.030971	0.317968	0.059001
0.04999	0.00332	0.08779	0.01448	0.066883	0.003569	0.123515	0.005804
0.21633	0.01229	0.17020	0.01986	0.20417	0.007025	0.174533	0.020683
0.12023	0.01957	0.07367	0.01323	0.162958	0.019596	0.094869	0.007775
0.04108	0.00846	0.40934	0.00752	0.062225	0.007295	0.289115	0.03729
ASP_out#1 C4H8N1O4		ASP_out#1 C4H8N1O4		ASP_out#1 C4H8N1O4		ASP_out#1 C4H8N1O4	
0.89691	0.04947	0.83388	0.04244	0.574381	0.052717	0.764965	0.061268
0.05327	0.01749	0.05737	0.01431	0.055142	0.020081	0.070008	0.009461
0.03255	0.01391	0.02317	0.00853	0.211209	0.045645	0.051392	0.015996
0.01173	0.01231	0.02562	0.01285	0.108741	0.044112	0.056717	0.028448
0.00553	0.00900	0.05996	0.02946	0.050528	0.045093	0.056919	0.029826
GLU#1111 C5H10N1O4		GLU#1111 C5H10N1O4		GLU#1111 C5H10N1O4		GLU#1111 C5H10N1O4	
0.57817	0.04462	0.19580	0.01834	0.476105	0.03151	0.291183	0.062427
0.04164	0.00290	0.06015	0.01245	0.037196	0.002414	0.106738	0.005222
0.23440	0.01582	0.04365	0.00792	0.242447	0.005663	0.068629	0.004993
0.05471	0.01018	0.17124	0.01951	0.077852	0.003896	0.153204	0.011741
0.06471	0.01414	0.01855	0.00176	0.097274	0.009613	0.014525	0.003604
0.02636	0.00563	0.51061	0.02488	0.069126	0.015597	0.365721	0.048244
CIT#11111 C6H7O7		CIT#11111 C6H7O7		CIT#11111 C6H7O7		CIT#11111 C6H7O7	
0.16879	0.03911	0.25351	0.00610	0.236079	0.031602	0.295872	0.054786
0.03297	0.01187	0.09895	0.00872	0.0426	0.002703	0.113117	0.011905
0.41922	0.04641	0.16824	0.01823	0.311324	0.030373	0.199336	0.043083
0.06802	0.00469	0.08570	0.00700	0.094705	0.003775	0.100375	0.006307
0.18719	0.01252	0.30086	0.01658	0.155446	0.003864	0.177466	0.019179
0.09399	0.01261	0.08063	0.01978	0.118082	0.006901	0.099936	0.010554
0.02982	0.00729	0.01210	0.00116	0.041764	0.003609	0.013898	0.004084
LAC#111 C3H5O3		LAC#111 C3H5O3		LAC#111 C3H5O3		LAC#111 C3H5O3	
0.06571	0.03881	0.94465	0.00647	0.101568	0.019322	0.851039	0.038283
0.00445	0.00174	0.03640	0.00112	0.010346	0.000977	0.043172	0.007699
0.01535	0.00024	0.00619	0.00138	0.022622	0.001962	0.018946	0.005688
0.91449	0.04073	0.01276	0.00408	0.865464	0.021101	0.086844	0.044086
GLU_out#1 C5H10N1O4		GLU_out#1 C5H10N1O4		GLU_out#1 C5H10N1O4		GLU_out#1 C5H10N1O4	
0.67994	0.03458	0.21177	0.03277	0.606	0.004991	0.665	0.055573
0.04493	0.00174	0.04287	0.00803	0.025	0.00765	0.067	0.006208
0.18363	0.01794	0.03503	0.00710	0.218	0.013044	0.026	0.004219
0.03438	0.00683	0.13522	0.02257	0.033	0.002717	0.058	0.015565
0.03844	0.00937	0.02020	0.00119	0.057	0.006093	0.022	0.009086
0.01867	0.00377	0.55490	0.00728	0.061	0.007714	0.163	0.041947
uM glutamate in media							
297.6855		274.3697		84.1405		55.27873	
305.0386		230.2176		59.6283		51.43023	
264.6086		288.1565		44.59167		64.71502	
282.9673		286.3838		56.98962		79.61804	
uM glutamate in initial media							
99.4439				1.841676			
106.1725				1.816122			
97.54374				1.723224			
116.1479				0.981384			
uM glutamate produced per well							
173.8515	23.26465428			60.45841	13.62049		
