# Supplementary material for: Glutamine addiction promotes glucose oxidation in triple-negative breast cancer
Source: Oncogene. 2022 Jul 18;41(34):4066–78. doi: 10.1038/s41388-022-02408-5 (PMC9391225; doi:10.1038/s41388-022-02408-5)
Supplement: Supplementary file 4 — Table S3 [file 41388_2022_2408_MOESM4_ESM.pdf]

Table S3: Relevant metabolic GO genesets significantly upregulated in TNBC GSEA analysis.

TCGA

NAME	SIZE_TCGA	ES_TCGA	NES_TCGA	NOM.p.val_TCGA	FDR.q.val_TCGA	FWER.p.val_TCGA	RANK.AT.MAX_TCGA	LEADING.EDGE_TCGA
GO_PURINE_CONTAINING_COMPOUND_METABOLIC_PROCESS	420	0.18484218	4.314165	0	0	0	16172	tags=48%, list=30%, signal=68%
GO_PYRUVATE_METABOLIC_PROCESS	144	0.2308509	3.2479827	0	2.83E-06	0.002	16172	tags=53%, list=30%, signal=75%
GO_PYRIMIDINE_CONTAINING_COMPOUND_METABOLIC_PROCESS	92	0.3006774	3.3890095	0	1.56E-06	0.001	14406	tags=57%, list=27%, signal=77%
GO_AMINO_ACID_TRANSMEMBRANE_TRANSPORT	80	0.23932679	2.484277	0	0.000389	0.395	18264	tags=58%, list=34%, signal=86%
GO_CELLULAR_AMINO_ACID_METABOLIC_PROCESS	304	0.15115756	2.945736	0	1.09E-05	0.01	16861	tags=46%, list=31%, signal=66%

METABRIC

NAME	SIZE_Metab	ES_Metabric	NES_Metabric	NOM.p.val_Metabric	FDR.q.val_Metabric	FWER.p.val_Metabric	RANK.AT.MAX_Metabric	LEADING.EDGE_Metabric
GO_PURINE_CONTAINING_COMPOUND_METABOLIC_PROCESS	387	0.19861926	4.5775747	0	0	0	6318	tags=45%, list=26%, signal=60%
GO_PYRUVATE_METABOLIC_PROCESS	137	0.29152253	3.941782	0	0	0	6631	tags=56%, list=27%, signal=77%
GO_PYRIMIDINE_CONTAINING_COMPOUND_METABOLIC_PROCESS	81	0.22930369	2.4046297	0	0.000998	0.58	7366	tags=53%, list=30%, signal=76%
GO_AMINO_ACID_TRANSMEMBRANE_TRANSPORT	73	0.20485301	2.0588377	0.008032128	0.007739006	1	7373	tags=51%, list=30%, signal=72%
GO_CELLULAR_AMINO_ACID_METABOLIC_PROCESS	278	0.17262903	3.343315	0	0	0	5833	tags=41%, list=24%, signal=53%

CCLE

NAME	SIZE_CCLE	ES_CCLE	NES_CCLE	NOM.p.val_CCLE	FDR.q.val_CCLE	FWER.p.val_CCLE	RANK.AT.MAX_CCLE	LEADING.EDGE_CCLE
GO_PURINE_CONTAINING_COMPOUND_METABOLIC_PROCESS	420	0.18483123	4.3452187	0	0	0	16172	tags=48%, list=30%, signal=68%
GO_PYRUVATE_METABOLIC_PROCESS	144	0.23083995	3.208506	0	0	0	16172	tags=53%, list=30%, signal=75%
GO_PYRIMIDINE_CONTAINING_COMPOUND_METABOLIC_PROCESS	92	0.3006676	3.3649569	0	0	0	14406	tags=57%, list=27%, signal=77%
GO_AMINO_ACID_TRANSMEMBRANE_TRANSPORT	80	0.23931439	2.494322	0	0.000353	0.37	18264	tags=58%, list=34%, signal=86%
GO_CELLULAR_AMINO_ACID_METABOLIC_PROCESS	306	0.15141116	3.0901	0	1.19E-06	0.001	16861	tags=46%, list=31%, signal=66%
