# Supplementary material for: Glutamine addiction promotes glucose oxidation in triple-negative breast cancer
Source: Oncogene. 2022 Jul 18;41(34):4066–78. doi: 10.1038/s41388-022-02408-5 (PMC9391225; doi:10.1038/s41388-022-02408-5)
Supplement: Supplementary file 5 — Table S4 [file 41388_2022_2408_MOESM5_ESM.pdf]

Table S4: Significant genes from Figure S5 heatmaps.

TCGA

Genes	logFC	logCPM	LR	PValue	FDR	FC
SLC1A6	5.9567	0.72468	424.731	2.28E-94	3.70E-93	62.1079
SLC6A15	5.67148	1.6358	657.49	5.25E-145	2.02E-143	50.9665
PSAT1	4.58861	4.27473	1550.75	0	0	24.0608
OQTLX	4.56905	3.78164	639.926	3.47E-141	1.26E-139	23.7367
PRKAG3	3.87192	-1.7973	221.903	3.48E-50	2.32E-49	14.6408
SULT1E1	3.77958	-0.3643	223.394	1.64E-50	1.11E-49	13.7331
THEM5	3.74359	-0.6708	783.353	2.25E-172	1.40E-170	13.3947
SLC17A6	3.57014	-3.0321	158.671	2.21E-36	1.04E-35	11.8773
ATCAY	3.53168	-2.0721	181.317	2.50E-41	1.34E-40	11.5649
IDO	3.47512	4.37593	583.744	5.75E-129	1.68E-127	11.1203
HPDL	3.29035	1.62742	695.834	2.41E-153	1.08E-151	9.78353
LOC115871	3.13352	6.69986	1038.43	7.96E-228	1.26E-225	8.77574
PPARGC1A	3.08537	1.86202	565.997	4.17E-125	1.16E-123	8.48765
PSMA8	3.0198	-2.3163	272.704	2.92E-61	2.48E-60	8.11054
SLC38A3	2.95635	2.48656	435.601	9.81E-97	1.65E-95	7.76156
XDH	2.75891	4.17008	360.472	2.22E-80	2.74E-79	6.76883
ACSL6	2.74395	0.70272	459.295	6.85E-102	1.26E-100	6.69902
SLC7A5	2.68603	7.15942	818.39	5.42E-180	3.78E-178	6.43541
APOBEC3B	2.63328	4.0896	692.238	1.46E-152	6.43E-151	6.20433
LDHB	2.59611	7.74777	1046.72	1.26E-229	2.08E-227	6.04656
SLC16A10	2.50302	0.52079	563.462	1.48E-124	4.06E-123	5.66869
FTCD	2.49556	-1.132	236.34	2.47E-53	1.77E-52	5.63948
IFNG	2.45747	-0.8733	223.091	1.92E-50	1.29E-49	5.49253
NOS1	2.4041	-1.7376	145.442	1.72E-33	7.45E-33	5.29306
CBS	2.39642	4.62266	402.734	1.40E-89	2.06E-88	5.26495
C14orf76	2.39349	-1.8602	196.73	1.08E-44	6.29E-44	5.25426
LOC93212	2.38369	2.73939	581.279	1.98E-128	5.75E-127	5.2187
PRTFDC1	2.31923	2.83098	886.298	9.34E-195	8.59E-193	4.99066
LOC1001323	2.31442	3.18567	718.215	3.27E-158	1.64E-156	4.97406
HACD1	2.30645	1.46259	491.688	6.12E-109	1.27E-107	4.94664
NTSR1	2.29776	-0.8324	165.415	7.42E-38	3.61E-37	4.91693
STGD2	2.27889	1.80914	508.146	1.61E-112	3.54E-111	4.85306
FLJ21145	2.1783	4.39737	1117.26	5.86E-245	1.29E-242	4.5262
LOC647046	2.12031	0.09642	389.69	9.67E-87	1.35E-85	4.34789
FOLH1B	2.09555	-2.0272	166.307	4.74E-38	2.32E-37	4.27388
HKDC1	2.07838	-1.7241	162.575	3.10E-37	1.49E-36	4.22334
AADAT	2.0585	2.27099	410.929	2.30E-91	3.50E-90	4.16552
PFKP	2.0541	6.072	604.591	1.68E-133	5.40E-132	4.15285
UROC1	2.05142	-2.7454	168.205	1.82E-38	9.03E-38	4.14513
ADORA2B	2.00207	1.6666	523.114	8.90E-116	2.08E-114	4.00573
NPPB	1.98794	-3.0685	69.0969	9.37E-17	2.38E-16	3.9667
LOC648732	1.97016	-2.6893	66.4182	3.65E-16	9.09E-16	3.91811
GUCA1A	1.95275	-2.2335	242.526	1.11E-54	8.07E-54	3.87111
GDA	1.91736	-1.948	78.5299	7.88E-19	2.16E-18	3.77732
APOBEC3A	1.8826	1.16951	215.256	9.80E-49	6.32E-48	3.68738
TH	1.8674	-0.445	67.5732	2.03E-16	5.10E-16	3.64874
NUDT11	1.8652	1.43737	354.029	5.62E-79	6.70E-78	3.64318
SLC16A1	1.82036	5.51418	437.472	3.84E-97	6.47E-96	3.53168
SLC17A1	1.81185	-3.1722	45.9658	1.20E-11	2.52E-11	3.51093
LOC115567	1.80591	5.33976	717.735	4.16E-158	2.08E-156	3.49649
ODC1	1.80586	5.90638	772.564	4.98E-170	2.96E-168	3.49637
TDO2	1.72639	2.39299	201.846	8.26E-46	4.94E-45	3.30899
TKTL1	1.72285	-1.6754	62.3911	2.82E-15	6.81E-15	3.30088
SLC36A2	1.71708	-2.6869	79.8017	4.14E-19	1.14E-18	3.28771
NOS2	1.69758	-0.1068	270.242	1.00E-60	8.45E-60	3.24356
CTPS1	1.69215	5.36599	1146.67	2.37E-251	6.08E-249	3.23138
ASNS	1.68887	5.26124	710.063	1.94E-156	9.40E-155	3.22404
RGS2	1.68335	5.13833	284.595	7.49E-64	6.66E-63	3.21174
KCNJ10	1.68282	0.48624	190.263	2.79E-43	1.57E-42	3.21054
GPT2	1.67366	5.66638	449.794	8.00E-100	1.42E-98	3.19023
SLC16A8	1.6726	-0.6546	226.986	2.71E-51	1.86E-50	3.18788
OGDHL	1.63256	0.48188	58.621	1.91E-14	4.47E-14	3.10063
SLC2A6	1.62886	2.82104	357.234	1.13E-79	1.37E-78	3.09267
LOC729351	1.62667	2.13141	525.922	2.18E-116	5.11E-115	3.08798
DGAT2	1.61982	5.44813	234.39	6.58E-53	4.66E-52	3.07336
SULT4A1	1.61936	0.70127	92.2751	7.54E-22	2.29E-21	3.07239
SLC7A5P1	1.60183	-2.813	101.929	5.75E-24	1.88E-23	3.03529
PDE7A	1.58666	5.74639	881.896	8.46E-194	7.71E-192	3.00354
WARS	1.56457	7.44787	692.061	1.59E-152	7.01E-151	2.95788
HK3	1.55165	2.22855	185.721	2.73E-42	1.50E-41	2.93152
LOC1001300	1.54818	7.72617	1027.59	1.81E-225	2.76E-223	2.92447
MGC21435	1.54643	-0.0838	135.57	2.48E-31	1.01E-30	2.92093
PDK1	1.49496	4.54998	634.169	6.20E-140	2.21E-138	2.81856
ASS1	1.47539	6.84993	185.306	3.36E-42	1.84E-41	2.7806
UCK2	1.47356	4.91726	961.642	3.91E-211	4.74E-209	2.77707
IL4I1	1.47111	3.67518	229.849	6.43E-52	4.46E-51	2.77235
LOC221268	1.46893	6.54367	703.639	4.83E-155	2.26E-153	2.76816
ENO1	1.46328	9.98236	849.79	8.08E-187	6.42E-185	2.75735
GLS	1.44678	6.16903	693.852	6.50E-153	2.90E-151	2.72599
DPYS	1.43926	-2.2378	115.624	5.75E-27	2.06E-26	2.71182
UPP1	1.43316	3.37581	385.031	9.99E-86	1.38E-84	2.70037
IDO2	1.41355	-1.768	71.1553	3.30E-17	8.52E-17	2.66391
SLC36A4	1.41198	3.02434	485.528	1.34E-107	2.72E-106	2.66101
LOC439932	1.37613	4.1152	463.463	8.48E-103	1.59E-101	2.59571
AICDA	1.3407	-0.9039	42.1505	8.45E-11	1.71E-10	2.53275
PAPSS1	1.3398	6.91676	991.235	1.44E-217	1.97E-215	2.53117
GAPDH	1.30642	11.5504	532.029	1.02E-117	2.46E-116	2.47326
SLC6A7	1.28308	-2.155	103.348	2.81E-24	9.25E-24	2.43357
TK1	1.26934	6.19577	267.005	5.10E-60	4.22E-59	2.41052
MCCC1	1.26833	5.1046	608.795	2.05E-134	6.68E-133	2.40883
ENPP3	1.26386	2.15763	59.5935	1.17E-14	2.75E-14	2.40137
DDIT4	1.24618	7.32939	199.015	3.43E-45	2.02E-44	2.37212
PSME4	1.24166	7.26895	761.516	1.26E-167	7.22E-166	2.3647
LRRC8D	1.23018	5.57947	659.821	1.63E-145	6.41E-144	2.34597
GMPS	1.22512	6.46428	872.019	1.19E-191	1.02E-189	2.33775
AK4	1.21768	4.14244	170.986	4.51E-39	2.27E-38	2.32572
GCSH	1.216	0.39136	186.501	1.85E-42	1.02E-41	2.32302
PLOD3	1.21411	6.36904	609.506	1.43E-134	4.68E-133	2.31998
GLDC	1.19929	3.02714	32.7489	1.05E-08	1.95E-08	2.29627
ACOT11	1.19871	1.82043	140.355	2.23E-32	9.37E-32	2.29535
GUCA2A	1.19536	-3.1789	35.602	2.42E-09	4.63E-09	2.29002
ME2	1.1866	5.35318	538.901	3.27E-119	8.12E-118	2.27616
HTR2A	1.17641	-1.3547	73.7854	8.71E-18	2.30E-17	2.26013
PSPH	1.16484	4.24086	314.375	2.43E-70	2.49E-69	2.24208
PDK3	1.16424	4.3902	327.797	2.90E-73	3.16E-72	2.24116
LOC88214	1.1593	6.18277	900.812	6.54E-198	6.35E-196	2.23349
AGPAT9	1.14676	2.25851	135.405	2.69E-31	1.09E-30	2.21416
ME1	1.14214	4.43742	111.982	3.61E-26	1.26E-25	2.20709
KYNU	1.1394	4.38339	70.0851	5.68E-17	1.45E-16	2.20289
LOC92051	1.13739	5.1563	154.843	1.51E-35	6.95E-35	2.19983
LOC56960	1.13488	0.33968	82.0551	1.32E-19	3.73E-19	2.196
NUP93	1.13232	5.65849	789.553	1.01E-173	6.46E-172	2.19212
AGPAT4	1.1271	3.37692	257.327	6.57E-58	5.15E-57	2.18419
EPHA2	1.12694	4.11747	317.082	6.26E-71	6.49E-70	2.18395
SLC25A19	1.12444	3.93116	441.104	6.23E-98	1.06E-96	2.18016
PGM1	1.12207	6.19055	480.743	1.47E-106	2.93E-105	2.17659
CMPK2	1.10806	4.39579	118.972	1.06E-27	3.89E-27	2.15555
LOC121572	1.10481	8.72358	528.285	6.67E-117	1.58E-115	2.1507
PRDX4	1.10373	6.63153	447.449	2.59E-99	4.54E-98	2.1491
ARG1	1.0995	-2.8484	39.6307	3.07E-10	6.09E-10	2.1428
SLC26A2	1.08998	5.61709	177.534	1.67E-40	8.75E-40	2.1287
ACSBG1	1.08301	-0.5847	63.9262	1.29E-15	3.16E-15	2.11844
BCAT1	1.08279	4.74665	134.768	3.71E-31	1.50E-30	2.11812
LGMBF	1.04204	5.23106	382.186	4.16E-85	5.67E-84	2.05914
HND	1.04166	-3.0193	21.3756	3.78E-06	6.22E-06	2.0586
PNP	1.02633	5.78649	335.119	7.38E-75	8.23E-74	2.03683
NT5C2	1.02156	6.12546	596.131	1.16E-131	3.60E-130	2.03011
PDE4B	1.01782	5.82034	89.5614	2.97E-21	8.84E-21	2.02485
ALDOC	1.01223	4.35924	74.9079	4.93E-18	1.31E-17	2.01703
MAT1A	1.00697	1.72271	30.9778	2.61E-08	4.77E-08	2.00968
KIAA0401	1.00673	5.5802	420.578	1.83E-93	2.91E-92	2.00935
PDHA1	1.0056	6.21633	701.026	1.79E-154	8.20E-153	2.00777
ACOT9	1.00361	4.58639	353.662	6.76E-79	8.04E-78	2.00501
ADA	1.0001	3.16611	227.229	2.40E-51	1.64E-50	2.00014
PSMB2	0.99392	6.77327	740.542	4.57E-163	2.48E-161	1.99158
DHTKD1	0.9931	6.4537	360.558	2.13E-80	2.62E-79	1.99046
MVD	0.98164	4.71328	151.827	6.91E-35	3.11E-34	1.97471
SLC32A1	0.97928	-3.0599	27.1118	1.92E-07	3.37E-07	1.97149
ADCY2	0.97793	3.25056	40.2076	2.28E-10	4.56E-10	1.96964

METABRIC

Genes	logFC	AveExpr	t	P.Value	adj.P.Val	FC
PSAT1	2.36673	6.47794	37.0123	2.87E-226	3.50E-222	5.15772
SLC7A5	2.2732	8.33628	28.8425	4.97E-152	1.51E-149	4.83395
PHGDH	2.12743	9.78879	26.1991	2.02E-129	3.24E-127	4.36937
PFKP	1.66436	8.31101	26.7279	7.23E-134	1.27E-131	3.16973
LDHB	1.55519	8.74187	21.7318	1.05E-93	6.31E-92	2.93872
ASS1	1.4906	8.30031	17.1724	1.48E-61	3.49E-60	2.81006
TYMS	1.36781	8.01311	25.2348	2.03E-121	2.54E-119	2.58079
IDO1	1.30365	6.3119	20.7838	1.29E-86	6.43E-85	2.46852
GPT2	1.17795	8.10229	19.2574	1.24E-75	4.49E-74	2.26254
ODC1	1.108	8.25929	20.9818	4.40E-88	2.27E-86	2.15546
APOBEC	1.10698	6.09075	23.8991	1.38E-110	1.36E-108	2.15394
CTPS	1.06909	7.39343	29.5862	1.51E-158	5.40E-156	2.09811
MTHFD1	1.03141	7.28237	27.5638	5.61E-141	1.16E-138	2.04402
TK1	0.99694	8.22729	17.6801	7.03E-65	1.85E-63	1.99577
WARS	0.99003	9.67475	20.2684	7.53E-83	3.32E-81	1.98623
PTPLA	0.97639	6.63946	17.828	7.34E-66	1.98E-64	1.96754
UPP1	0.9668	8.02227	23.4713	3.48E-107	3.22E-105	1.95451
UCK2	0.96438	8.29617	25.3663	1.68E-122	2.18E-120	1.95122
ASNS	0.95374	8.55755	19.4047	1.14E-76	4.27E-75	1.93688
ENO1	0.95342	12.4276	26.6151	6.47E-133	1.11E-130	1.93646
PNP	0.9396	8.09176	22.3879	1.02E-98	6.87E-97	1.918
PSME4	0.91768	9.3228	23.0196	1.26E-103	9.93E-102	1.88908
GMPS	0.90128	8.5863	25.6625	5.98E-125	8.72E-123	1.86772
SLC2A6	0.8965	6.67094	20.9566	6.77E-88	3.49E-86	1.86154
PDE9A	0.89376	6.62044	18.4439	5.23E-70	1.63E-68	1.85802
GAPDH	0.88824	10.4	20.4234	5.62E-84	2.55E-82	1.85092
NUP93	0.88276	8.15098	28.658	2.01E-150	5.63E-148	1.8439
TDO2	0.84423	6.33957	14.9326	9.26E-48	1.42E-46	1.7953
PSMB9	0.82345	7.91306	12.5026	1.59E-34	1.61E-33	1.76964
PLOD2	0.81382	9.51252	12.4125	4.50E-34	4.49E-33	1.75786
DDIT4	0.79363	9.47288	12.0341	3.38E-32	3.15E-31	1.73343
SHMT2	0.78612	9.93409	23.3997	1.28E-106	1.17E-104	1.72443
SLC16A3	0.78603	9.04087	12.9835	5.41E-37	5.95E-36	1.72432
NUP205	0.77921	8.95581	25.3089	4.98E-122	6.39E-120	1.71619
ALDOC	0.77894	7.86984	11.3172	8.80E-29	7.21E-28	1.71587
PRDX4	0.77092	10.7553	19.5149	1.90E-77	7.30E-76	1.70636
NUP62	0.7652	6.07157	18.9925	8.75E-74	3.04E-72	1.6996
ADA	0.76074	7.5621	19.4504	5.41E-77	2.06E-75	1.69436
AMD1	0.75509	7.05056	14.8762	1.97E-47	2.99E-46	1.68773
ACOT9	0.75496	7.06567	19.4321	7.29E-77	2.75E-75	1.68759
KYNU	0.75368	6.48434	11.7463	8.34E-31	7.37E-30	1.68609
SLC7A1	0.7483	8.42772	17.6328	1.44E-64	3.75E-63	1.67981
PSMB2	0.73404	9.72258	22.8301	3.80E-102	2.88E-100	1.66329
NUDT1	0.71927	8.66795	20.4864	1.95E-84	8.97E-83	1.64635
AARS	0.71593	10.7	20.994	3.57E-88	1.86E-86	1.64255
MCCC1	0.71321	8.43178	20.0082	5.71E-81	2.39E-79	1.63945
GARS	0.69289	10.0689	22.2185	2.05E-97	1.34E-95	1.61651
AK4	0.66787	8.17881	8.73719	5.14E-18	2.74E-17	1.58873
KARS	0.66513	7.97438	23.86	2.83E-110	2.79E-108	1.58571
TPI1	0.65924	10.8589	12.1581	8.30E-33	7.90E-32	1.57925
ACOT7	0.65454	8.98722	13.8999	6.64E-42	8.53E-41	1.57412
SLC25A1	0.6538	6.9752	17.2598	4.05E-62	9.71E-61	1.57331
ADORA2	0.64741	5.91148	19.9289	2.12E-80	8.76E-79	1.56635
PLOD1	0.64096	10.1345	16.1579	3.97E-55	7.62E-54	1.55937
NME1	0.63913	11.3134	13.4003	3.42E-39	4.05E-38	1.55739
NUP155	0.62035	7.72232	20.3136	3.54E-83	1.57E-81	1.53724
HK3	0.61915	6.02012	17.1021	4.22E-61	9.79E-60	1.53597
PDHA1	0.61629	8.30758	21.2384	5.41E-90	2.99E-88	1.53293
SLC16A1	0.61573	6.41345	17.149	2.10E-61	4.92E-60	1.53233
SLC25A1	0.61286	7.28501	16.5049	2.71E-57	5.53E-56	1.52928
GPI	0.61055	9.69884	15.6107	8.79E-52	1.54E-50	1.52684
PGK1	0.60872	9.2842	9.06893	2.92E-19	1.64E-18	1.52491
ME1	0.60546	6.35483	12.2668	2.40E-33	2.34E-32	1.52146
RGS2	0.59994	8.53422	8.31349	1.75E-16	8.65E-16	1.51565
ENTPD5	-0.5853	6.34062	-12.881	1.84E-36	1.98E-35	-1.5004
HAGH	-0.5908	9.50696	-16.149	4.49E-55	8.58E-54	-1.5061
GMPR2	-0.6075	9.15047	-17.202	9.49E-62	2.25E-60	-1.5237
ME3	-0.6104	7.01338	-11.656	2.26E-30	1.96E-29	-1.5267
HNMT	-0.6147	8.14641	-11.992	5.45E-32	5.05E-31	-1.5312
BCAT2	-0.6259	8.52453	-15.589	1.19E-51	2.08E-50	-1.5432
AK5	-0.6321	5.98778	-12.051	2.80E-32	2.62E-31	-1.5498
ELOVL2	-0.6619	6.00893	-12.597	5.25E-35	5.42E-34	-1.5822
GLUD1	-0.6825	9.9747	-20.05	2.85E-81	1.20E-79	-1.6049
IVD	-0.6854	7.33473	-18.368	1.71E-69	5.27E-68	-1.6082
FAH	-0.6907	7.9355	-13.371	4.92E-39	5.81E-38	-1.6141
JMJD8	-0.6948	11.3027	-20.823	6.62E-87	3.32E-85	-1.6187
DALRD3	-0.7015	7.37509	-18.752	4.04E-72	1.32E-70	-1.6262
NUDT18	-0.7019	7.43632	-18.97	1.26E-73	4.37E-72	-1.6267
ACOT1	-0.7076	7.32643	-14.747	1.10E-46	1.63E-45	-1.6331
SLC1A1	-0.7119	6.10967	-11.162	4.58E-28	3.66E-27	-1.638
SLC1A4	-0.7127	7.1097	-14.524	2.12E-45	3.00E-44	-1.6389
HSD17B8	-0.7353	7.50578	-18.581	6.05E-71	1.94E-69	-1.6647
ACADSB	-0.7364	6.20229	-21.74	9.14E-94	5.51E-92	-1.666
ACACB	-0.7594	7.81118	-12.194	5.52E-33	5.29E-32	-1.6927
THNSL2	-0.7632	8.85899	-11.189	3.42E-28	2.75E-27	-1.6973
ADCY9	-0.8492	7.07083	-21.606	9.33E-93	5.45E-91	-1.8015
HDC	-0.8505	6.62045	-10.127	1.64E-23	1.11E-22	-1.8031
HSD17B4	-0.8517	9.59389	-21.234	5.82E-90	3.19E-88	-1.8046
GAD1	-0.8796	5.82512	-11.995	5.23E-32	4.85E-31	-1.8398
ALDH6A1	-0.9387	8.41383	-22.569	4.09E-100	2.90E-98	-1.9168
TAT	-0.9762	6.2687	-8.3493	1.31E-16	6.50E-16	-1.9673
ABAT	-1.0286	6.23404	-25.951	2.38E-127	3.61E-125	-2.04
CSAD	-1.0443	8.63219	-19.253	1.32E-75	4.78E-74	-2.0624
NME3	-1.1649	10.4218	-27.974	1.67E-144	3.77E-142	-2.2422
ELOVL5	-1.2654	8.95081	-18.353	2.15E-69	6.59E-68	-2.404
ACOT4	-1.3865	8.23508	-26.061	2.86E-128	4.53E-126	-2.6145
QDPR	-1.4206	9.51639	-19.687	1.13E-78	4.47E-77	-2.6769
ADCY1	-1.4208	6.90688	-15.714	2.08E-52	3.73E-51	-2.6773
SLC7A8	-1.575	8.10241	-27.972	1.75E-144	3.91E-142	-2.9793
HMGCS2	-1.7502	7.49665	-11.574	5.48E-30	4.71E-29	-3.364
FBP1	-1.7831	9.6654	-28.599	6.54E-150	1.77E-147	-3.4417
SLC7A2	-2.4761	8.67038	-19.527	1.55E-77	5.98E-76	-5.5641

CCLE

Genes	logFC	logCPM	LR	PValue	FDR	FC
SLC1A3	5.57603	4.21658	25.3275	4.84E-07	0.00014	47.7038
BCAT1	5.18777	4.15162	16.0928	6.03E-05	0.00429	36.448
NT5E	4.66406	5.6869	17.5793	2.76E-05	0.00253	25.3526
GFPT2	4.39324	4.14745	18.4532	1.74E-05	0.00186	21.0134
ACSL5	4.19003	4.00124	16.1315	5.91E-05	0.00422	18.2525
PSAT1	3.904	7.27045	27.9157	1.27E-07	4.82E-05	14.97
ACSL4	3.5787	4.85934	19.7188	8.97E-06	0.00118	11.948
DPYD	3.54905	4.04445	18.9821	1.32E-05	0.00154	11.705
UPP1	3.11646	4.73674	21.164	4.22E-06	0.00072	8.67259
ADORA2	3.01814	4.94643	22.2787	2.36E-06	0.00046	8.10122
RGS2	2.94272	3.75141	14.2778	0.00016	0.00865	7.68858
EPHA2	2.68943	6.17115	21.265	4.00E-06	0.00069	6.4506
AGPAT4	2.60184	2.92499	15.6992	7.43E-05	0.00504	6.07059
PGM1	2.28499	5.57024	25.2777	4.96E-07	0.00015	4.8736
GLS	2.16066	6.19418	21.2527	4.03E-06	0.00069	4.4712
ADCY7	2.12744	3.66953	15.1384	9.99E-05	0.00623	4.36943
ADA	2.05068	3.98685	14.7055	0.00013	0.00734	4.143
ODC1	1.68412	6.96696	14.7923	0.00012	0.00709	3.21345
SEH1L	1.50125	4.88944	28.3497	1.01E-07	4.11E-05	2.83088
TYMS	1.40021	8.30327	24.989	5.77E-07	0.00016	2.6394
PSPH	1.37936	5.2755	14.1784	0.00017	0.00896	2.60153
AGPAT5	1.31648	4.85942	16.2644	5.51E-05	0.00406	2.49057
MCCC2	-1.1285	6.30315	17.8436	2.40E-05	0.0023	-2.1863
FAH	-1.2382	4.7051	16.7265	4.32E-05	0.00341	-2.359
PER2	-1.2856	3.8183	18.3287	1.86E-05	0.00193	-2.4378
PSMB3	-1.3878	9.1531	14.9551	0.00011	0.00664	-2.6167
PPT1	-1.391	8.19117	19.8483	8.38E-06	0.00112	-2.6226
ACOT2	-1.4405	5.33998	28.0042	1.21E-07	4.74E-05	-2.7141
NUDT4	-1.4564	6.17323	20.8737	4.91E-06	0.0008	-2.7443
ADCY6	-1.515	5.95516	32.8119	1.02E-08	6.73E-06	-2.8579
PPM1K	-1.5874	3.37479	17.3523	3.11E-05	0.00273	-3.005
SLC26A1	-1.6507	3.09168	17.779	2.48E-05	0.00237	-3.1399
ACOT1	-1.691	4.91408	22.5303	2.07E-06	0.00042	-3.2288
CSAD	-1.8023	4.7822	32.1271	1.44E-08	8.72E-06	-3.4878
ACADSB	-1.8077	4.57888	18.6801	1.55E-05	0.00173	-3.5009
ACACB	-2.1114	3.46557	18.0241	2.18E-05	0.00213	-4.3211
ACOT4	-2.133	4.00915	21.562	3.43E-06	0.00063	-4.3863
DDC	-2.8731	2.92388	15.5043	8.23E-05	0.00541	-7.3265
HGD	-2.9437	3.90952	19.1203	1.23E-05	0.00147	-7.6939
ABAT	-3.0245	3.72979	45.4618	1.56E-11	4.72E-08	-8.137
ACOT6	-3.0412	2.75562	21.2613	4.01E-06	0.00069	-8.2316
IYD	-3.1231	2.61028	18.1684	2.02E-05	0.00202	-8.7124
SLC7A8	-3.5866	4.61101	31.9049	1.62E-08	9.46E-06	-12.014
AICDA	-3.9504	2.56637	17.3755	3.07E-05	0.00271	-15.459
HMGCS2	-7.7785	4.91165	69.3656	8.18E-17	4.45E-12	-219.56

SLC7A1	0.97408	6.15807	243.673	6.22E-55	4.56E-54	1.96439
LRRC8C	0.96602	4.61202	231.902	2.29E-52	1.61E-51	1.95344
HPD	0.95379	0.57604	18.2878	1.90E-05	3.00E-05	1.93695
NUP205	0.94856	6.50538	417.143	1.02E-92	1.60E-91	1.92995
ATP5CL1	0.94421	7.57925	515.262	4.54E-114	1.03E-112	1.92413
PLOD2	0.94147	6.46637	122.934	1.44E-28	5.41E-28	1.92049
LDHC	0.9413	-0.1414	24.9324	5.94E-07	1.02E-06	1.92025
DFNB89	0.93665	7.15043	581.206	2.05E-128	5.95E-127	1.91407
TBPL1	0.93466	3.61943	503.737	1.46E-111	3.17E-110	1.91144
LOC115890	0.9327	8.1334	382.75	3.13E-85	4.28E-84	1.90885
GMPR	0.93134	3.59148	85.4272	2.40E-20	6.95E-20	1.90704
CMTDIC	0.93003	6.73474	539.162	2.87E-119	7.14E-118	1.90532
SLC25A13	0.92385	4.95649	423.859	3.53E-94	5.71E-93	1.89717
ADCY7	0.92262	5.05765	201.375	1.05E-45	6.25E-45	1.89556
LOC100507	0.92113	1.21437	127.811	1.23E-29	4.77E-29	1.8936
SEH1L	0.91979	5.67419	475.412	2.13E-105	4.16E-104	1.89184
MRX63	0.91361	5.35249	182.926	1.11E-41	6.01E-41	1.88376
GARS	0.91043	7.11217	435.897	8.46E-97	1.42E-95	1.8796
GUCY2F	0.91029	-3.2113	32.1088	1.46E-08	2.70E-08	1.87942
CAD	0.90984	6.28791	469.932	3.32E-104	6.33E-103	1.87884
ABCD1	0.90812	4.79964	186.004	2.37E-42	1.30E-41	1.8766
GPI	0.90214	8.48345	308.44	4.78E-69	4.75E-68	1.86883
NT5C1B	0.89723	-2.1864	66.8641	2.91E-16	7.28E-16	1.86248
LOC115206	0.89419	3.91907	171.732	3.10E-39	1.57E-38	1.85856
GART	0.8913	6.43516	529.82	3.09E-117	7.40E-116	1.85484
SHMT2	0.88537	6.61465	355.229	3.08E-79	3.70E-78	1.84724
SLC7A11	0.88186	3.51752	52.9436	3.43E-13	7.68E-13	1.84275
DNPH1	0.87275	4.68441	116.212	4.27E-27	1.53E-26	1.83116
SLC16A3	0.8705	5.61457	88.0523	6.37E-21	1.88E-20	1.8283
ATP5J2	0.86284	6.5758	224.794	8.14E-51	5.52E-50	1.81862
FLJ22583	0.84048	6.09258	339.512	8.15E-76	9.22E-75	1.79064
PanK	0.83806	2.93087	87.7883	7.28E-21	2.14E-20	1.78765
APP	0.83663	10.2728	221.637	3.97E-50	2.65E-49	1.78588
DIPP	0.81908	5.12054	382.624	3.34E-85	4.56E-84	1.76429
AK2	0.80996	7.03309	463.965	6.59E-103	1.24E-101	1.75316
PSMD7	0.80448	6.45239	360.222	2.52E-80	3.10E-79	1.74652
HPRT1	0.80183	5.43564	265.106	1.32E-59	1.08E-58	1.74331
AARS	0.7965	7.43666	246.07	1.87E-55	1.39E-54	1.73688
NDC1	0.79027	4.7022	165.587	6.81E-38	3.32E-37	1.7294
GAPDHS	0.78957	-2.3897	54.0945	1.91E-13	4.31E-13	1.72856
AC3	0.78881	5.69459	225.24	6.51E-51	4.43E-50	1.72765
NUP155	0.78873	5.39927	181.516	2.26E-41	1.21E-40	1.72756
SLC6A9	0.78862	4.93131	67.6641	1.94E-16	4.87E-16	1.72743
ACOT7	0.7719	5.09846	136.847	1.30E-31	5.36E-31	1.70752
ELOVL7	0.77107	3.55719	61.0146	5.67E-15	1.35E-14	1.70654
LDHAL6B	0.76809	-2.6143	55.1164	1.14E-13	2.59E-13	1.70302
RAB23	0.76587	4.65969	173.497	1.27E-39	6.52E-39	1.7004
STOML2	0.75958	6.52878	308.81	3.97E-69	3.96E-68	1.69299
LOC130203	0.75936	2.40904	30.9123	2.70E-08	4.93E-08	1.69274
ADSL	0.75731	5.50504	412.831	8.87E-92	1.36E-90	1.69033
SLC1A3	0.74477	4.16808	83.8303	5.39E-20	1.54E-19	1.6757
PTS	0.74122	3.978	203.217	4.15E-46	2.50E-45	1.67159
VARS	0.73733	6.71394	269.156	1.73E-60	1.45E-59	1.66709
CYC1	0.73705	7.09837	144.871	2.29E-33	9.91E-33	1.66676
ELOVL1	0.73371	6.67859	352.195	1.41E-78	1.67E-77	1.66291
ERO1L	0.73151	5.57584	112.131	3.35E-26	1.17E-25	1.66037
PRPS2	0.73095	6.00255	181.412	2.38E-41	1.27E-40	1.65973
RAN	0.72885	7.85363	347.873	1.23E-77	1.43E-76	1.65732
MGC9344	0.72859	6.88771	269.285	1.62E-60	1.36E-59	1.65701
LOC115049	0.72613	7.11607	91.5609	1.08E-21	3.27E-21	1.65419
SLC17A3	0.72495	-2.6294	8.02495	0.0046137	0.0062825	1.65284
SLC2A9	0.72413	1.83562	116.935	2.97E-27	1.07E-26	1.65191
DKFZp434D	0.72003	6.60852	249.83	2.83E-56	2.14E-55	1.64721
UNG	0.71974	5.99968	248.25	6.25E-56	4.71E-55	1.64688
PSMA7	0.71903	7.43922	186.978	1.45E-42	8.04E-42	1.64607
PID1	0.71825	2.47404	56.2743	6.30E-14	1.45E-13	1.64518
KDM1A	0.71426	6.76941	402.493	1.58E-89	2.32E-88	1.64064
ARG2	0.70965	3.30619	148.838	3.11E-34	1.38E-33	1.6354
MPST	0.70622	5.27493	102.042	5.44E-24	1.78E-23	1.63152
NPPC	0.70271	-2.1417	10.7663	0.0010336	0.001472	1.62756
NUP153	0.69436	6.43481	176.743	2.49E-40	1.30E-39	1.61816
PGK1	0.69322	8.94122	184.238	5.76E-42	3.12E-41	1.61689
SCD5	0.69035	4.31122	40.9421	1.57E-10	3.15E-10	1.61367
LOC124499	0.68958	4.17237	92.8362	5.68E-22	1.73E-21	1.61281
PPAT	0.68943	4.80017	163.33	2.12E-37	1.02E-36	1.61265
LOC116122	0.67907	-2.2515	22.5078	2.09E-06	3.49E-06	1.60111
CDA	0.67632	1.10513	56.6151	5.30E-14	1.22E-13	1.59806
PSMD2	0.67471	8.06165	301.706	1.40E-67	1.35E-66	1.59627
NT5E	0.67164	4.40073	64.8787	7.97E-16	1.96E-15	1.59289
FLJ14977	0.67061	5.72499	151.12	9.87E-35	4.42E-34	1.59174
ATP5G3	0.66935	7.29641	269.87	1.21E-60	1.02E-59	1.59036
PRPSAP1	0.66911	5.67106	231.901	2.29E-52	1.61E-51	1.59009
EPRS	0.66403	8.1151	190.952	1.97E-43	1.11E-42	1.5845
ADI1	0.6583	6.23362	142.704	6.82E-33	2.91E-32	1.57822
LOC132947	0.63595	7.6157	169.53	9.37E-39	4.68E-38	1.55396
DBI	0.6349	7.54142	88.6751	4.65E-21	1.38E-20	1.55283
SLC38A5	0.63172	3.2247	32.2839	1.33E-08	2.47E-08	1.54941
AMPD2	0.6289	5.31066	226.348	3.73E-51	2.55E-50	1.54639
LOC654081	0.62838	3.75439	40.0658	2.46E-10	4.90E-10	1.54583
NUP85	0.62391	5.51503	194.367	3.54E-44	2.04E-43	1.54105
CARS2	0.62336	5.38314	135.746	2.27E-31	9.25E-31	1.54046
GUCY1A3	0.62206	5.98173	47.7589	4.82E-12	1.03E-11	1.53907
MNGIE	0.62183	6.75946	48.4178	3.44E-12	7.38E-12	1.53883
CMPK1	0.61199	7.39811	263.848	2.49E-59	2.02E-58	1.52836
NUDT15	0.60704	4.52816	160.575	8.47E-37	4.02E-36	1.52313
LOC137671	0.60681	4.48296	88.3245	5.56E-21	1.64E-20	1.52289
FLJ20839	0.60677	-0.1033	10.7437	0.0010463	0.0014892	1.52285
QRSL1	0.6041	4.89343	100.327	1.29E-23	4.16E-23	1.52003
PSMD14	0.597	6.27767	263.241	3.37E-59	2.73E-58	1.51256
FARSB	0.59527	5.95371	258.837	3.08E-58	2.44E-57	1.51075
SEC13	0.5916	6.99853	202.961	4.72E-46	2.84E-45	1.50691
LOC129820	0.59046	5.46653	179.207	7.22E-41	3.81E-40	1.50573
GCLM	0.59	4.35517	98.1816	3.82E-23	1.21E-22	1.50525
SLC6A12	0.58719	1.1923	21.9206	2.84E-06	4.71E-06	1.50232
LOC221413	0.58575	6.38841	163.952	1.55E-37	7.49E-37	1.50082
COASY	-0.5868	6.48816	118.402	1.42E-27	5.16E-27	-1.5019
ABHD14B	-0.5873	6.3458	103.14	3.12E-24	1.03E-23	-1.5025
SEPHS2	-0.5874	7.41171	97.711	4.84E-23	1.53E-22	-1.5026
TK2	-0.5966	5.29376	209.492	1.77E-47	1.10E-46	-1.5122
HMGCLL1	-0.6036	1.05463	10.5345	0.0011717	0.0016633	-1.5195
SCDOS	-0.6048	9.70618	26.5308	2.59E-07	4.53E-07	-1.5207
CLN3	-0.6224	6.63268	109.332	1.37E-25	4.70E-25	-1.5395
ADSSL1	-0.6261	2.85458	45.1681	1.81E-11	3.77E-11	-1.5434
NOX4	-0.6394	3.42271	46.9108	7.43E-12	1.57E-11	-1.5577
SLC25A2	-0.6488	-2.7667	21.9896	2.74E-06	4.54E-06	-1.5679
SUCLG2	-0.6491	6.18895	177.037	2.15E-40	1.12E-39	-1.5682
GGT5	-0.659	5.45401	63.7558	1.41E-15	3.44E-15	-1.579
CDS1	-0.6591	5.97288	132.917	9.43E-31	3.76E-30	-1.5791
AOX1	-0.664	3.60099	20.1518	7.15E-06	1.16E-05	-1.5845
ADAL	-0.6647	3.20288	153.801	2.56E-35	1.17E-34	-1.5852
THNSL2	-0.6688	5.52482	56.6727	5.15E-14	1.18E-13	-1.5897
ACSBG2	-0.673	-2.4152	33.282	7.97E-09	1.49E-08	-1.5943
AASS	-0.6751	3.45663	73.3865	1.07E-17	2.80E-17	-1.5967
LOC139770	-0.683	1.38578	22.7018	1.89E-06	3.16E-06	-1.6055
PKLR	-0.6837	-2.3222	11.0314	0.0008958	0.0012814	-1.6063
RIMKLA	-0.6838	2.85453	27.0338	2.00E-07	3.51E-07	-1.6064
GPAM	-0.7022	4.88127	72.1586	1.99E-17	5.17E-17	-1.6269
ATP7A	-0.7082	4.97961	124.883	5.40E-29	2.05E-28	-1.6337
ENPP4	-0.7094	5.08041	59.4855	1.23E-14	2.91E-14	-1.6351
HAGH	-0.7207	5.36883	115.205	7.10E-27	2.54E-26	-1.648
SCZD9	-0.7271	3.81094	38.5417	5.36E-10	1.05E-09	-1.6553
SERINC5	-0.7284	3.4152	45.1473	1.83E-11	3.80E-11	-1.6568
GMPR2	-0.7317	6.10677	345.414	4.23E-77	4.87E-76	-1.6605
DKFZp547O	-0.7317	5.61638	407.199	1.49E-90	2.24E-89	-1.6606
PGAM2	-0.7357	1.32565	33.4729	7.23E-09	1.36E-08	-1.6652
GLUD2	-0.7423	4.60337	130.795	2.74E-30	1.08E-29	-1.6728
HMGCL	-0.7488	5.50881	215.178	1.02E-48	6.57E-48	-1.6804
HAL	-0.7494	-0.9164	15.3988	8.70E-05	0.0001326	-1.6811
NUDT16	-0.7565	5.1359	313.654	3.49E-70	3.56E-69	-1.6894
JMJD8	-0.761	6.66224	192.421	9.42E-44	5.36E-43	-1.6947
BCAT2	-0.7695	5.50996	114.386	1.07E-26	3.81E-26	-1.7047
GLUD1	-0.7767	7.7825	254.273	3.04E-57	2.35E-56	-1.7132
TREM2	-0.7874	4.367	89.6732	2.81E-21	8.38E-21	-1.726

GSTZ1	-0.8077	4.72813	114.525	1.00E-26	3.56E-26	-1.7504
THTPA	-0.8206	4.33832	225.411	5.97E-51	4.06E-50	-1.7661
SLC25A21	-0.8214	-0.1992	38.9803	4.28E-10	8.45E-10	-1.7671
PANK3	-0.8316	5.76731	134.482	4.29E-31	1.73E-30	-1.7797
PADI4	-0.8386	-2.1485	14.1572	0.0001682	0.0002524	-1.7883
PER2	-0.8409	5.31629	222.651	2.39E-50	1.60E-49	-1.7912
FLJ45829	-0.8429	3.75011	51.4728	7.26E-13	1.60E-12	-1.7937
C14ORF159	-0.8453	5.14445	186.051	2.31E-42	1.27E-41	-1.7967
ME3	-0.8581	3.52745	82.4266	1.10E-19	3.09E-19	-1.8126
SDSL	-0.8659	4.1627	124.286	7.29E-29	2.76E-28	-1.8225
LOC1005065	-0.8666	5.53046	165.067	8.84E-38	4.30E-37	-1.8233
NQO1	-0.8821	7.60348	72.5271	1.65E-17	4.30E-17	-1.8431
CBR4	-0.8979	4.93384	246.651	1.39E-55	1.04E-54	-1.8633
AS1	-0.8982	0.2303	43.1342	5.11E-11	1.04E-10	-1.8637
ADSD	-0.9032	2.68611	33.2125	8.26E-09	1.55E-08	-1.8702
OTC	-0.911	-3.1002	18.3722	1.82E-05	2.88E-05	-1.8804
ALDH4A1	-0.9149	5.49258	130.605	3.02E-30	1.19E-29	-1.8854
TYR	-0.9225	-2.7203	11.3635	0.000749	0.0010773	-1.8954
MTHFR	-0.9241	5.25865	210.43	1.11E-47	6.93E-47	-1.8975
SLC43A1	-0.9276	4.03352	87.4238	8.76E-21	2.57E-20	-1.9021
PDZD3	-0.937	-2.7687	40.7142	1.76E-10	3.53E-10	-1.9146
ADCY4	-0.9377	3.09638	126.894	1.96E-29	7.53E-29	-1.9155
ADCY6	-0.9534	6.5961	283.374	1.38E-63	1.22E-62	-1.9364
G6PC	-0.9787	-3.0947	12.4761	0.0004122	0.0006037	-1.9706
SEPSECS	-0.9917	4.14206	380.171	1.14E-84	1.54E-83	-1.9886
ACOT2	-1.0095	4.35377	280.387	6.18E-63	5.42E-62	-2.0132
SLC38A1	-1.0194	8.76372	133.184	8.24E-31	3.29E-30	-2.0271
MAGI3	-1.0311	5.80608	162.65	2.98E-37	1.43E-36	-2.0436
MLXIPL	-1.0315	1.48362	41.5934	1.12E-10	2.27E-10	-2.0442
NUDT4	-1.0326	5.7884	313.203	4.38E-70	4.45E-69	-2.0457
PDE4A	-1.0396	4.83877	125.521	3.91E-29	1.49E-28	-2.0557
HSD17B4	-1.0409	7.06174	370.338	1.58E-82	2.02E-81	-2.0575
LOC138369	-1.0413	-0.996	26.5941	2.51E-07	4.39E-07	-2.0581
HNMT	-1.0605	5.49658	334.866	8.38E-75	9.33E-74	-2.0856
PTHLH	-1.0801	4.21448	31.9541	1.58E-08	2.91E-08	-2.1141
NUDT18	-1.0954	3.37968	182.78	1.20E-41	6.46E-41	-2.1368
SLC1A4	-1.1041	6.96925	188.68	6.17E-43	3.45E-42	-2.1497
GUCY1A2	-1.1269	1.50516	72.7733	1.45E-17	3.81E-17	-2.184
LOC145480	-1.1316	5.04267	255.015	2.09E-57	1.63E-56	-2.191
MACROD2	-1.164	2.72004	78.0309	1.01E-18	2.77E-18	-2.2407
IGF1	-1.175	4.61396	86.3274	1.52E-20	4.44E-20	-2.258
PDE5A	-1.2048	4.97663	90.9102	1.50E-21	4.53E-21	-2.3051
DLX6	-1.2056	5.71832	86.8405	1.18E-20	3.44E-20	-2.3063
CRYM	-1.2059	1.93715	25.7338	3.92E-07	6.79E-07	-2.3069
NEIL1	-1.2068	2.85614	161.14	6.38E-37	3.03E-36	-2.3083
APOBEC2	-1.2115	-1.9984	52.1324	5.19E-13	1.15E-12	-2.3158
ACSF2	-1.2192	5.67924	165.77	6.21E-38	3.03E-37	-2.3281
LOC119865	-1.2286	3.09206	169.087	1.17E-38	5.83E-38	-2.3434
PDE1A	-1.2313	1.72174	177.361	1.83E-40	9.53E-40	-2.3477
PRKAA2	-1.2345	4.26153	70.2425	5.24E-17	1.34E-16	-2.3529
HSD17B8	-1.2378	3.82655	223.556	1.52E-50	1.02E-49	-2.3584
NME3	-1.2552	5.88865	174.79	6.65E-40	3.43E-39	-2.3869
FAH	-1.2652	5.98004	275.282	8.01E-62	6.87E-61	-2.4036
SLC38A4	-1.2655	0.22334	67.5619	2.04E-16	5.13E-16	-2.4041
DALRD3	-1.2713	5.58612	524.144	5.31E-116	1.24E-114	-2.4137
NAGS	-1.2875	2.23035	126.976	1.88E-29	7.23E-29	-2.4411
CORD5	-1.3105	-1.0889	77.0403	1.68E-18	4.53E-18	-2.4803
LOC1005065	-1.3136	4.51805	295.173	3.71E-66	3.47E-65	-2.4856
NOXRED1	-1.315	-0.9009	263.998	2.31E-59	1.88E-58	-2.488
SLC7A3	-1.3207	-0.7209	37.5929	8.72E-10	1.70E-09	-2.4979
ERO1LB	-1.3649	4.41019	172.046	2.64E-39	1.34E-38	-2.5757
SULT1A1	-1.3804	4.84373	205.006	1.69E-46	1.03E-45	-2.6033
SLC1A7	-1.3808	-0.1545	139.257	3.87E-32	1.61E-31	-2.6041
SCZD4	-1.4006	4.34934	53.4289	2.68E-13	6.02E-13	-2.6401
ABCG2	-1.4086	2.15828	156.224	7.57E-36	3.50E-35	-2.6548
ACSM5	-1.4181	0.44579	141.86	1.04E-32	4.43E-32	-2.6723
ADCY10	-1.4257	-1.1824	68.1986	1.48E-16	3.73E-16	-2.6864
ASPA	-1.4259	-0.758	125.014	5.05E-29	1.92E-28	-2.6868
SLC19A2	-1.4262	6.09747	292.593	1.35E-65	1.25E-64	-2.6873
GLUL	-1.4335	10.4117	391.595	3.72E-87	5.24E-86	-2.701
ACACB	-1.4347	4.88172	185.319	3.34E-42	1.83E-41	-2.7033
PGK2	-1.4363	-2.7844	33.6642	6.55E-09	1.23E-08	-2.7063
SARDH	-1.4379	2.85944	132.178	1.37E-30	5.43E-30	-2.7093
PTH	-1.4569	-3.1498	11.9252	0.0005538	0.000804	-2.7451
ADCY9	-1.4856	5.79827	464.115	6.12E-103	1.15E-101	-2.8004
PCK1	-1.4941	1.30512	38.9113	4.44E-10	8.74E-10	-2.8169
SULT2B1	-1.4951	3.92543	119.18	9.56E-28	3.51E-27	-2.8188
IVD	-1.5217	6.65602	630.664	3.58E-139	1.26E-137	-2.8712
RCVRN	-1.5373	-2.2356	140.116	2.51E-32	1.05E-31	-2.9024
MYOG	-1.5394	-2.1808	30.6139	3.15E-08	5.73E-08	-2.9067
FASN	-1.5757	10.1289	190.39	2.61E-43	1.47E-42	-2.9809
SLC26A1	-1.5869	1.59697	309.806	2.41E-69	2.41E-68	-3.0041
DDO	-1.6167	1.68001	233.988	8.05E-53	5.68E-52	-3.0667
SLC3A1	-1.6332	0.12714	68.2338	1.45E-16	3.67E-16	-3.1021
CBFA2T3	-1.6362	3.89288	164.723	1.05E-37	5.10E-37	-3.1084
CSAD	-1.6415	4.80551	301.567	1.50E-67	1.44E-66	-3.1199
SLC22A12	-1.6434	-3.0263	42.7524	6.21E-11	1.27E-10	-3.1239
GPD1	-1.6454	4.33183	64.4625	9.84E-16	2.42E-15	-3.1283
SLC22A11	-1.7049	-2.1312	110.62	7.17E-26	2.47E-25	-3.2602
QDPR	-1.7179	6.39712	265.549	1.06E-59	8.68E-59	-3.2895
ACOT6	-1.7473	-1.5055	129.727	4.70E-30	1.84E-29	-3.3572
GADL1	-1.7651	-2.446	25.6876	4.01E-07	6.95E-07	-3.3989
GA	-1.7784	2.14098	247.41	9.53E-56	7.14E-55	-3.4305
SLC25A18	-1.8057	0.86542	137.613	8.86E-32	3.66E-31	-3.4959
C1orf36	-1.8583	-2.1959	113.146	2.00E-26	7.04E-26	-3.6257
AK5	-1.8597	3.70799	115.63	5.73E-27	2.05E-26	-3.6292
SULT1A2	-1.8665	1.12629	196.233	1.39E-44	8.05E-44	-3.6465
ENTPD5	-1.9032	4.34943	274.641	1.11E-61	9.47E-61	-3.7404
PFKFB1	-1.9163	0.0437	147.746	5.39E-34	2.37E-33	-3.7747
PAH	-1.9298	1.14842	56.6692	5.16E-14	1.19E-13	-3.8101
COT	-1.9484	5.13168	460.95	2.99E-102	5.55E-101	-3.8594
GLYAT	-2.004	-0.9963	66.4768	3.54E-16	8.83E-16	-4.011
MCCC2	-2.0474	7.94756	650.76	1.53E-143	5.69E-142	-4.1337
ACOT4	-2.0542	3.91292	591.813	1.01E-130	3.07E-129	-4.1532
ENPP1	-2.1033	6.71638	433.33	3.06E-96	5.09E-95	-4.2969
ELOVL5	-2.1071	8.69543	527.845	8.31E-117	1.97E-115	-4.3084
SLC1A2	-2.1524	4.51923	169.231	1.09E-38	5.43E-38	-4.4458
GLYATL1	-2.2277	3.04519	126.072	2.97E-29	1.13E-28	-4.684
C6orf71	-2.26	1.86623	89.6602	2.83E-21	8.43E-21	-4.79
HOGA1	-2.3053	-0.059	191.969	1.18E-43	6.70E-43	-4.9425
AGXT2	-2.398	-2.8398	92.4672	6.84E-22	2.08E-21	-5.2706
NME5	-2.4447	2.384	275.899	5.88E-62	5.06E-61	-5.4442
ACSM6	-2.4944	-2.5644	55.8005	8.02E-14	1.84E-13	-5.6348
SCP	-2.6347	1.51754	99.2224	2.26E-23	7.21E-23	-6.2103
LOC654185	-2.6734	6.95553	664.784	1.36E-146	5.51E-145	-6.3793
HGD	-2.6792	3.38374	155.959	8.64E-36	3.99E-35	-6.4049
FBP1	-2.7106	6.55085	727.171	3.69E-160	1.92E-158	-6.5458
SLC7A4	-2.7624	1.96698	114.351	1.09E-26	3.88E-26	-6.7851
ADCY5	-2.9184	3.41461	209.803	1.52E-47	9.46E-47	-7.5602
SLC38A11	-3.1095	-0.7547	242.009	1.43E-54	1.04E-53	-8.6309
SLC7A8	-3.1407	7.33188	1012.12	4.16E-222	6.09E-220	-8.8192
SLC1A1	-3.1762	5.76895	235.961	2.99E-53	2.14E-52	-9.039
LOC116200	-3.1831	2.36505	211.752	5.70E-48	3.60E-47	-9.0824
TPH2	-3.1977	-2.6322	74.8605	5.05E-18	1.34E-17	-9.1752
ELOVL2	-3.2689	5.59838	257.451	6.17E-58	4.85E-57	-9.639
GNMT	-3.5319	1.71391	336.57	3.56E-75	3.99E-74	-11.567
DFNB44	-3.5628	5.97908	383.077	2.66E-85	3.65E-84	-11.817
ACSM1	-3.664	3.41555	274.076	1.47E-61	1.25E-60	-12.675
NPD009	-3.7953	6.241	903.036	2.15E-198	2.14E-196	-13.883
TPH1	-4.0905	0.88605	170.759	5.05E-39	2.54E-38	-17.036
SLC7A2	-4.4951	8.14784	482.238	6.96E-107	1.40E-105	-22.55
SLC7A13	-4.6287	-1.1929	541.766	7.78E-120	1.97E-118	-24.739
TTR	-4.9016	-1.1832	120.111	5.98E-28	2.21E-27	-29.89
AGXT	-5.0061	-0.6385	111.034	5.82E-26	2.01E-25	-32.137
HDC	-5.7207	3.73194	361.817	1.13E-80	1.40E-79	-52.734
TAT	-5.9385	6.31524	210.405	1.12E-47	7.02E-47	-61.329
ACMSD	-5.954	0.02362	245.778	2.16E-55	1.60E-54	-61.993
INS	-6.4412	-1.329	56.4445	5.78E-14	1.33E-13	-86.898
HMGCS2	-6.7599	6.08411	327.256	3.81E-73	4.12E-72	-108.37
