# Supplementary material for: Glutamine addiction promotes glucose oxidation in triple-negative breast cancer
Source: Oncogene. 2022 Jul 18;41(34):4066–78. doi: 10.1038/s41388-022-02408-5 (PMC9391225; doi:10.1038/s41388-022-02408-5)
Supplement: Supplementary file 6 — Table S5 [file 41388_2022_2408_MOESM6_ESM.pdf]

Table S5

TCGA DATASET

GENE NAME	TNBC_Average_Rank	TNBC_Percentile	LumA_Average_Rank
ALDOA	20406.98925	0.995414333	20450.96631
ALDOC	12459.12366	0.607732484	10319.2766
BPGM	13779.72581	0.672148959	13277.50177
DLAT	16291.89247	0.794687697	15053.82447
DLD	18127.38172	0.88421939	17600.15603
ENO1	20450.59677	0.997541426	20316.08688
ENO2	13522.51613	0.659602757	14552.39184
ENO3	7623.795699	0.371874333	7731.498227
GAPDH	20491.58065	0.999540542	20472.04255
GPI	20134.67742	0.982131477	19828.34929
HK1	19310.54839	0.941932022	19714.39716
HK2	17767.67204	0.866673433	17933.65603
HK3	9343.83871	0.455774777	7252.484043
HK4	4319.822581	0.210712774	4627.361702
LDHA	20334.95161	0.991900474	20281.2695
LDHB	20035.99462	0.977317917	17529.87766
LDHC	5914.155914	0.288481338	4883.757092
PDHA1	18224.0914	0.888936705	16400.41135
PDK1	14862.18817	0.724949425	11349.39716
PFKFB1	4637.741935	0.226220279	5840.297872
PFKFB2	14310.25269	0.698027057	13129.55674
PFKFB3	17332.34946	0.845439221	18080.79433
PFKFB4	10803.19892	0.526959608	10668.58865
PFKL	19415.6129	0.947056871	19106.30142
PFKM	14657.9086	0.714985054	15165.59574
PFKP	18086.43011	0.882221848	14032.44681
PGAM1	19372.95161	0.944975934	19108.10993
PGK1	20229.27957	0.986745991	20070.79255
PKM	20426.96774	0.996388846	20407.84574
SLC16A1	17190.57527	0.838523744	12926.73759
SLC16A3	15459.7043	0.754095132	13563.68085
SLC16A7	6639.080645	0.323841795	6367.026596
SLC2A1	18810.51613	0.917541395	17626.17021
SLC2A13	9663.188172	0.47135204	10209.06206
SLC2A3	14445.13441	0.704606332	13570.52128
SLC2A4	6236.526882	0.304205984	6538.303191
SLC2A5	9629.510753	0.469709319	7694.212766
SLC2A6	10536.91935	0.513970994	8279.193262
SLC2A8	10945.71505	0.533911275	12050.90248
TIGAR	12165.54839	0.593412438	11174.88652
TPI1	20249.45699	0.987730208	19965.38652

METABRIC DATASET

Hugo_Symbc	TNBC_Average_Rank	TNBC_Percentile	LumA_Average_Rank
ALDOA	23708.91367	0.972952793	23862.88978
ALDOC	20669.89568	0.848239317	19005.82565
BPGM	16880.6259	0.692737438	16641.95792
DLAT	18406.91367	0.75537236	17892.58317
DLD	19526.97482	0.801336787	19231.78958
ENO1	24317.05036	0.997909158	24242.83367
ENO2	18647.78777	0.765257213	19711.82565
ENO3	17109.3777	0.702124823	17834.17234
GAPDH	23897.0036	0.98067152	23453.49299
GPI	23421.18705	0.961145234	22947.41884
HK1	24058.90647	0.987315597	24139.92385
HK2	17891.79856	0.734233362	17788.0501
HK3	15438.53957	0.633557927	11540.51703
HK4	5385.985612	0.221026987	5591.242485
LDHA	24270.42806	0.995995899	24248.39078
LDHB	22777.83094	0.934743554	20582.51703
LDHC	12291.28417	0.504402666	11124.86573
PDHA1	21791.91007	0.8942839	20724.96794
PDK1	13506.34173	0.554265501	13145.62926
PFKFB1	7945.719424	0.326071874	9654.991984
PFKFB2	13083.63309	0.536918627	11934.1523
PFKFB3	22450.2518	0.92130055	22816.50701
PFKFB4	20062.72302	0.823322514	20203.02605
PFKL	21238.6223	0.871578394	20544.18236
PFKM	17542.09353	0.719882367	17715.69339
PFKP	22605.93525	0.927689398	19906.15631
PGAM1	19466.98921	0.798875132	18779.14629
PGK1	22725.36691	0.932590566	22091.16232
PKM	23236.64388	0.953572057	23072.11022
SLC16A1	4914.611511	0.201683007	4663.358717
SLC16A3	22646.5	0.929354071	21630.72946
SLC16A7	6635.111511	0.272287899	6210.971944
SLC2A1	22344.83094	0.916974349	21244.81964
SLC2A13	6511.503597	0.267215348	6559.721443
SLC2A3	22602.92446	0.927565843	21725.45892
SLC2A4	6582.834532	0.270142586	6694.873747
SLC2A5	18164.66547	0.745431117	15547.73347
SLC2A6	18399.44964	0.755066055	15693.30461
SLC2A8	20739.00719	0.851075476	21435.58317
TIGAR	16896.66187	0.693395513	16085.56513
TPI1	23949.69065	0.982833661	23755.53707

CCLE DATASET

Hugo_Symbc	TNBC_Average_Rank	TNBC_Percentile	LumA_Average_Rank

ALDOA	56080.96154	0.995791071	56142.8
ALDOC	46440.92308	0.824619537	47769.5
BPGM	50963.57692	0.904925191	50193.1
DLAT	53004.38462	0.94116241	51670
DLD	54233.53846	0.96298765	54020.1
ENO1	56252.57692	0.998838327	56163.3
ENO2	50619.26923	0.898811556	49211
ENO3	45002.84615	0.799084594	44181.3
GAPDH	56296.11538	0.99961141	56297
GPI	55841.30769	0.991535702	55693.3
HK1	53685.88462	0.953263337	55176.2
HK2	50407	0.895042438	51458.2
HK3	28908.61538	0.513310405	29715.9
HK4	29566	0.524983132	29217.5
LDHA	56107.03846	0.996254101	55909.2
LDHB	53529.76923	0.950491304	49444.4
LDHC	34208.23077	0.607412031	34615.8
PDHA1	54268.96154	0.963616633	54499.2
PDK1	43950.26923	0.780394709	42672.7
PFKFB1	36716.11538	0.651942814	38222.2
PFKFB2	46889.61538	0.832586658	47606.8
PFKFB3	51438.5	0.913358074	52977.5
PFKFB4	44837.80769	0.796154119	46008.2
PFKL	55047.84615	0.977446752	55450.1
PFKM	50941.57692	0.904534552	52704.2
PFKP	54062.5	0.959950637	51270.7
PGAM1	55501.46154	0.985501288	55515.8
PGK1	55696.34615	0.98896172	55721.6
PKM	56199.11538	0.997889048	56177.3
SLC16A1	51933.42308	0.922146083	45132.1
SLC16A3	47976.88462	0.85189255	46680.6
SLC16A7	37760.34615	0.670484501	34624.3
SLC2A1	54073.61538	0.960148006	54800.8
SLC2A13	40559.5	0.720187152	42730.9
SLC2A3	41705.73077	0.740539983	37013.3
SLC2A4	37527.42308	0.666348647	36936.5
SLC2A5	33130.73077	0.588279605	30898.6
SLC2A6	48055.5	0.853288469	46979.8
SLC2A8	46058.73077	0.817833211	48543.5
TIGAR	49185.57692	0.873354468	49258.7
TPI1	56036.26923	0.9949975	55854.1

LumA_Percentile	PValue	FDR	FC
0.997559451	3.35E-11	6.90E-11	-1.366975
0.503354792	4.93E-18	1.31E-17	2.01703213
0.647651421	2.27E-11	4.71E-11	1.22532489
0.734297082	2.33E-37	1.12E-36	1.49066604
0.858502318	1.92E-21	5.75E-21	1.3061018
0.990980288	8.08E-187	6.42E-185	2.75734872
0.709838147	0.00597605	0.00805729	-1.2288005
0.377127858	0.00034629	0.00050959	-1.3005962
0.998587511	1.02E-117	2.46E-116	2.47326311
0.967189371	4.78E-69	4.75E-68	1.86883416
0.961631002	7.57E-13	1.67E-12	-1.2467926
0.874769817	0.25350716	0.29065626	-1.0633068
0.353762453	2.73E-42	1.50E-41	2.93151721
0.225713951	0.81124064	0.84362936	-1.0225512
0.989281962	4.49E-20	1.28E-19	1.41826022
0.855074272	1.26E-229	2.08E-227	6.04655802
0.238220433	5.94E-07	1.02E-06	1.9202528
0.799981042	1.79E-154	8.20E-153	2.00777393
0.553602125	6.20E-140	2.21E-138	2.81856229
0.284878683	5.39E-34	2.37E-33	-3.774652
0.640434942	2.34E-05	3.68E-05	1.27822985
0.881946945	3.25E-09	6.18E-09	-1.435843
0.520393574	0.09857731	0.11913313	1.07338788
0.931969241	2.94E-20	8.48E-20	1.39351279
0.739749073	0.24519645	0.28171068	-1.0536126
0.684476211	1.68E-133	5.40E-132	4.15285386
0.932057457	1.60E-25	5.47E-25	1.31850957
0.979015295	5.76E-42	3.12E-41	1.61688723
0.995456112	3.12E-21	9.27E-21	1.3748972
0.630541807	3.84E-97	6.47E-96	3.53168399
0.661610695	6.37E-21	1.88E-20	1.82829634
0.310571513	0.02548683	0.0326534	1.18927077
0.859771241	3.14E-73	3.41E-72	2.54333896
0.497978736	1.16E-06	1.97E-06	-1.3368773
0.661944358	1.53E-06	2.57E-06	1.3103857
0.318926062	0.20208506	0.23478472	-1.120073
0.375309144	1.65E-39	8.42E-39	2.56802615
0.403843386	1.13E-79	1.37E-78	3.09267476
0.587820227	1.19E-20	3.49E-20	-1.4536512
0.545089826	9.02E-26	3.11E-25	1.4387293
0.973873788	1.66E-120	4.17E-119	2.17013

LumA_Percentile	P.Value	adj.P.Val	FC
0.979271577	0.03196957	0.06056398	-1.1249325
0.779950166	8.80E-29	7.21E-28	1.71587259
0.682943119	6.06E-05	0.00015642	1.07725134
0.73426556	1.15E-14	5.24E-14	1.15446884
0.789223144	0.00096516	0.00225494	1.10082886
0.994863496	6.47E-133	1.11E-130	1.93646108
0.808922589	7.56E-09	2.52E-08	-1.3145612
0.73186853	2.10E-08	6.83E-08	-1.1801613
0.962470986	5.62E-84	2.55E-82	1.85092195
0.941703005	8.79E-52	1.54E-50	1.52684309
0.990640342	8.81E-14	3.83E-13	-1.1711801
0.729975792	0.12502833	0.20265334	1.05349385
0.473593115	4.22E-61	9.79E-60	1.53596518
0.2294502	0.37357482	0.49557794	-1.0079129
0.995091546	2.27E-14	1.02E-13	1.23709652
0.844653522	1.05E-93	6.31E-92	2.93872139
0.456535856	0.02506998	0.04846916	1.05708711
0.850499341	5.41E-90	2.99E-88	1.53293333
0.53946279	0.0001804	0.0004482	1.03818115
0.39621602	9.79E-05	0.00024841	-1.0767894
0.489746894	2.54E-05	6.74E-05	1.06757039
0.936330721	1.08E-05	2.93E-05	-1.2491165
0.829080189	0.8598117	0.90825459	-1.0050507
0.843080366	3.77E-32	3.51E-31	1.29023163
0.727006459	0.65266347	0.75065387	-1.0117093
0.816897419	7.23E-134	1.27E-131	3.16972996
0.770647829	4.92E-08	1.56E-07	1.23686412
0.906564442	2.92E-19	1.64E-18	1.5249073
0.946820019	6.58E-08	2.08E-07	1.16990445
0.191372239	0.33544129	0.45588041	1.00716242
0.887669462	5.41E-37	5.95E-36	1.72431823
0.254882302	0.04345418	0.08006135	1.01365527
0.871832717	8.54E-43	1.13E-41	1.75882353
0.269194084	0.87483474	0.91860098	1.00121639
0.891556916	1.17E-26	8.86E-26	1.53398184
0.274740387	0.52710913	0.64203365	-1.0048897
0.638038964	6.04E-56	1.19E-54	1.77647783
0.644012829	6.77E-88	3.49E-86	1.86154494
0.879661161	5.31E-13	2.22E-12	-1.2337788
0.660110191	3.10E-16	1.52E-15	1.20523342
0.974866098	8.30E-33	7.90E-32	1.57925431

LumA_Percentile	PValue	FDR	FC

0.996889094	0.20199855	1	-1.2306538
0.848210164	0.84765859	1	1.08651819
0.891244362	0.06232569	0.51406425	1.63192104
0.91746866	0.03167693	0.33424325	1.49988637
0.95919777	0.3967243	1	1.14639972
0.997253098	0.00054376	0.0210218	2.27123142
0.873805888	0.141293	0.8369396	1.82786819
0.784496964	0.07203648	0.56057482	1.88887925
0.999627117	0.17054793	0.93009964	-1.2788684
0.988907632	0.51227962	1	1.12162318
0.979725843	0.02343897	0.27634248	-1.5736197
0.913707873	0.53540458	1	-1.1897508
0.527644803	0.78158325	1	-1.0961477
0.518795057	0.94817901	1	-1.0263835
0.99274122	0.00063108	0.02328802	2.15792168
0.877950211	0.02377657	0.27865446	3.71682061
0.614648958	0.75156443	1	1.1730453
0.967704819	0.9043198	1	-1.0218792
0.757709791	0.06846418	0.54422917	1.7602024
0.678685323	0.63794187	1	-1.2647322
0.845321212	0.79880824	1	-1.0584677
0.940685039	0.01310793	0.18879035	-1.8998011
0.816935971	0.82097353	1	-1.0689642
0.984589296	0.23244039	1	-1.2194424
0.935832238	0.18653603	0.97691036	-1.3342002
0.910378565	0.01644326	0.21858391	2.07980305
0.985755886	0.6625153	1	1.07939424
0.989410135	0.32188032	1	1.1912348
0.997501687	0.23646624	1	1.30959761
0.801379665	0.00404739	0.08524239	3.49814163
0.828875315	0.05399095	0.46993311	2.64815099
0.614799886	0.0227958	0.27143232	3.31910927
0.973060123	0.35607923	1	-1.2793925
0.758743208	0.4942673	1	-1.2610549
0.657219717	0.0004503	0.01845908	12.8677875
0.655856032	0.89773119	1	1.06550486
0.548645193	0.36897033	1	1.57726341
0.834188004	0.57648449	1	1.1844918
0.861953549	0.00661814	0.12023253	-1.718736
0.874652864	0.34108453	1	1.2568351
0.991762847	0.00554791	0.10624061	1.69121164
