# Supplementary material for: Glutamine addiction promotes glucose oxidation in triple-negative breast cancer
Source: Oncogene. 2022 Jul 18;41(34):4066–78. doi: 10.1038/s41388-022-02408-5 (PMC9391225; doi:10.1038/s41388-022-02408-5)
Supplement: Supplementary file 7 — Supplementary Methods [file 41388_2022_2408_MOESM7_ESM.docx]

Supplementary Materials & Methods

Inhibitors

Inhibitors were resuspended in H_2_O and diluted in media to final concentrations as follows: 2-amino-2-norbornanecarboxylic acid (BCH, Sigma-Aldrich), 10 mM; benzylserine (Bachem Swiss), 10 mM; sulfasalazine (Sigma-Aldrich), 0.5 mM; L-γ-glutamyl-p-nitroanilide (GPNA, Sigma-Aldrich), 1 mM; 1 mM DL-cycloserine (CS, Sapphire Bioscience); and 1 mM aminooxyacetic acid hemihydrochloride (AOA, Merck). Erastin (Merck) was resuspended in DMSO and diluted to a final concentration of 10 μM Erastin in 0.1% DMSO.

Seahorse Fuel Flex analysis

Oxygen consumption rate (as a proxy measure of mitochondrial respiration; OCR) and extracellular acidification rate (as an estimation of lactate production; ECAR) were measured in live cells using the Seahorse Mito Fuel Flex kit with a Seahorse XFp Analyser. Measurements were performed according to manufacturers’ instructions, with measurements taken when the metabolism of glutamine is inhibited with 3 μM BPTES (dependency), or when glutamine metabolism is permitted while glucose and fatty acid metabolism pathways are inhibited with 4 μM UK5099 and 2 μM etomoxir (capacity). Similar measurements were made by switching the order of inhibitors to determine glucose or fatty acid dependency and capacity. Briefly, cells were seeded into a Seahorse XFp Miniplate (2 × 10^4^ cells/well) in full growth medium and allowed to adhere overnight. Growth media was then removed and replaced with pre-warmed running medium (consisting of non-buffered DMEM (Sigma-Aldrich, MO) supplemented with 1 mM sodium pyruvate and 10 mM glucose, pH 7.4. The Miniplate was then incubated for 60 min in a non-CO_2_ incubator at 37°C before OCR and ECAR measurements were taken at 6.5 min intervals for 100 min, with the first inhibitor/s injected at 26 min, and then the remaining inhibitor/s injected at 65 min. Data analysis was performed according to manufacturers’ instructions, where capacity is greater than dependency, the difference between these measurements determines the flexibility in fuel usage.

^14^C_5_-glutamine tracer experiments

Cells were plated in triplicate at a density of 7 × 10^5^ cells/well in 6-well plates in normal growth media and allowed to adhere at 37°C for 12-18 h. Growth media was removed, the cell monolayer was washed once with PBS and media was then replaced with glutamine-free RPMI 1640 (LifeTech) containing 2% (w/v) bovine serum albumin (BSA; Amresco), and 2 mM L-glutamine (Gibco). *In vitro* labelling proceeded in one of three ways: (a) a 15 min labelling experiment, (b) a 4 h labelling experiment, or (c) a 15 min labelled pulse with 4 h unlabelled chase. In all experiments, 0.5 μCi L-[^14^C_5_ (U)]-Glutamine (PerkinElmer) was included in the labelled media. Where inhibitors were used, they were diluted 1:10 in the media described above, and sterile endotoxin-free tissue culture grade water (Sigma) was used as a vehicle control.

At the end of each experiment, the carbon pool was resolved into six fractions using a TRIzol® extraction protocol modified from Hosios *et al* (2016)^1^. These fractions were: extracellular dissolved CO_2_, intracellular RNA, DNA and protein, and the intracellular polar and organic pools (Fig. S1a).

After removal of the extracellular medium, the cell monolayer was washed once with PBS and then homogenised in 1 mL TRIzol®. A guanidium thiocyanate-phenol-chloroform extraction was then performed according to manufacturers’ instructions. After centrifugation, RNA was precipitated from the top aqueous phase by addition of isopropanol (Sigma) and DNA was precipitated from the interphase/lower organic phase material by addition of 100% ethanol (Sigma), with glycogen included as a carrier to improve yield (Astral Scientific). After overnight precipitation, RNA pellets were briefly washed once with 70% ethanol, air dried and then resuspended in sterile nuclease-free water, while DNA pellets were washed twice with 70% ethanol for 10 min at room temperature with gentle inversion, air dried and then resuspended in 8 mM sodium hydroxide solution. The aqueous supernatant from the RNA extraction tubes was removed and transferred to new tubes as the “polar” fraction. Protein was then precipitated from the DNA supernatant by the addition of 3 volumes of acetone, followed by 20 min incubation at room temperature and then centrifugation to sediment protein and insoluble material. The supernatant was removed and transferred to new tubes as the “organic” intracellular fraction. Precipitated protein pellets were washed three times in 95% ethanol/2.5% glycerol/0.3 M guanidine hydrochloride wash buffer, followed by a fourth and final wash using wash buffer without guanidine hydrochloride (97.5% ethanol/2.5% glycerol). The protein pellets were air dried briefly and then solubilised in cell lysis buffer to give the “protein” fraction.

For pulse-chase experiments, an additional seventh fraction was also collected which measured extracellular effluxed carbon (Fig. S1a). Extracellular media was split into two aliquots, one transferred undiluted to a scintillation vial as the “media” fraction, while the other was used as a measure of complete oxidation by the addition of an equal volume of 1 M perchloric acid to the extracellular media to liberate ^14^CO_2_ in gaseous form, which was then captured in 1 N sodium hydroxide.

For all phases, ^14^C activity was measured by liquid scintillation counting in a Tri-Carb® Liquid Scintillation Analyser (PerkinElmer). The average counts per minute (CPM) from each tube was used to calculate the ^14^C activity of each sample in equivalent pmol of ^14^C-glutamine. These calculations were based on empirically determined specific activity (decays per minute per picomole; dpm/pmol) of a ^14^C-glutamine standard, which was measured for each experiment to ensure consistency and accuracy of counting.

Explant ^13^C-glutamine tracer experiments

Balb/c female nude mice (Animal Resource Centre, Perth, Australia) 8-12 weeks of age were housed in a specific pathogen-free facility at Lowy Cancer Centre in accordance with the University of New South Wales animal ethics committee guidelines (Approval No. 18/61A). On day 0, mice were anaesthetised via 2% isoflurane inhalation, 2 × 10^6^ HCC1806 cells or 4 × 10^6^ MCF-7 cells suspended in a solution of 50% Matrigel in PBS (100 µL) were then implanted orthotopically into the left and right abdominal mammary glands of mice. To facilitate tumour growth, 1 µM 17-β-estradiol was added to the drinking water of mice implanted with MCF-7 cells. Tumour tissues were collected within 5 weeks post-injection. The tumours were dissected into 1 mm^3^ pieces and four pieces placed on pre-soaked gelatin sponges (Spongostan, Henry Schein) in wells of a 24-well plate with 0.5 mL media. Sponges were pre-soaked in HPLM, Human Plasma-like media (HPLM[^35^](#_ENREF_35)) containing 0.5 mM L-[^13^C_5_(U)]-glutamine, 10% dialysed FBS (Life Technologies) and 100 units/mL penicillin-streptomycin. The explants were cultured for 16 hours in a 37 °C incubator with 5% CO_2_. After 16 hours tumour explants were collected and snap-frozen in CK28R Precellys tubes with 2.8 mm ceramic beads. Extracellular media were collected as well and set aside for extraction and LC-MS analysis. Tumour explants were then homogenised in 250 µl of extraction solvent (40% Methanol: 40% Acetonitrile: 20% MilliQ water containing internal standard) at 0°C using the Precellys®24 homogeniser along with the Cryolys. The tubes were centrifuged at 16000 g for 15 minutes at 4°C, after which the supernatants were collected and dried in preparation for subsequent LC-MS analysis.

**Cell lysates extraction for GC-MS and LC-MS**

Metabolites were extracted using water, methanol and chloroform. Extracellular media were collected as well and set aside for extraction and LC-MS analysis as detailed above. The cell monolayer was washed once with ice-cold 0.9% (w/v) NaCl solution and then rapidly lysed in 50% (v/v) methanol:water mixture that had been prechilled to -30°C. Internal standards chlorophenylalanine and norvaline (Sigma; 1 or 2 nmoles/well) were added to each well. Cells were scraped into this mixture and then the entire volume was transferred to prechilled Falcon tubes and kept on ice. Each well was then rinsed once with equal volume ice-cold UltraPure^TM^ water (ThermoFisher) and this was combined with the first extract. One volume of prechilled chloroform was then added to each tube. The extraction mixes were vortexed vigorously for 10 sec and centrifuged at 3200 g for 5 min. The aqueous phase of each sample was then transferred to a prechilled glass tube, gradually cooled to -30°C and then evaporated to dryness without heat using a SpeedVac. For GC-MS, dried samples were derivatised using tBDMS silylation and then promptly analysed as described previously^2^. For LC-MS, dried samples were resuspended in LC-MS grade H_2_O before being injected.

**Measuring stable-isotope labelled metabolites by LC-MS**

An aliquot (10 μl) of extracellular media was set aside and all remaining media removed. The aliquot was mixed with 40 μL of extraction buffer containing 1:1 v/v acetonitrile and methanol at -30°C. The slurry was centrifuged at 12000 g for 5 min at 4°C to pellet precipitates, and supernatants were transferred into HPLC vials for LC-MS analysis. Naïve media was extracted in the same manner to quantify the amounts consumed or produced. Cell monolayers were immediately washed once with ice-cold 0.9% NaCl, and rapidly lysed with 600 μL ice-cold extraction buffer (40% Methanol: 40% Acetonitrile: 20% MilliQ water containing internal standard). Cells were scraped into this mixture and then transferred to prechilled tubes. All wells were then each washed with an additional 600 μL of extraction buffer, and this was then combined with the first extract. These mixtures were centrifuged at 12000 g for 5 min at 4°C to pellet cellular debris and then supernatants were transferred to new tubes and dried in preparation for LC-MS analysis.

Extracellular media and cell lysates were quantified by different platforms of LC-MS/MS depending on analytes being assayed. Amino acids and nucleotides were quantified using a Vanquish-TSQ Altis (ThermoFisher) triple-quadrupole mass spectrometer with LC separation achieved on XBridge Amide LC column (3.5 µm, 4.6 mm × 100 mm, Waters). The LC buffers and gradient used are based on a protocol described previously^3^. Glycolytic and TCA cycle metabolites were quantified using 1260 Infinity LC System (Agilent) coupled to QTRAP5500 (ABSciex) mass spectrometer. LC separation was achieved on a Synergi Hydro-RP LC column (2.5 µm, 2.0 mm × 100 mm, Phenomenax). The LC buffers and gradient used are based on a protocol described previously^4^. For both methods, dried samples were resuspended in LC-MS grade H_2_O before being injected. MRMs were configured depending on the labelled substrates applied.

*In silico* gene expression analysis

Gene expression data from The Cancer Genome Atlas (TCGA; Firehose Legacy dataset, data accessed June 2020), Molecular Taxonomy of Breast Cancer International Consortium (METABRIC^5, 6^, data accessed June 2020)^5, 6^, as well as all breast cancer cell lines included in The Cancer Cell Line Encyclopedia (CCLE^7^) were retrieved using cBioPortal (<http://www.cbioportal.org/>^8, 9^). Molecular subtyping of data from TCGA samples utilised PAM50 molecular subtype data (accessed via cBioPortal), while METABRIC and CCLE samples were classified as previously published^10, 11^. Differential expression analysis was conducted using the R bioconductor packages edgeR or limma, to identify genes that were biologically and statistically significant (FC>=1.5 and FDR<0.01, respectively) between TNBC and Luminal A breast cancer subtypes. LogFC ranking metric was used for performing a Gene Set Enrichment Analysis (GSEA) on MSigDB (v7.1) Hallmark, C2 (Curated) and C5 (Gene Ontology) datasets^12, 13^. Relevant metabolic gene sets that were significantly enriched were extracted and those with FWER<0.001 or FDR<0.001 were examined further. Enrichment traces from GSEA were plotted using RToolbox and gene expression data of genes involved in amino acid metabolic pathways were plotted using ComplexHeatmap package with rows ordered by decreasing fold-change, and columns ordered by unsupervised hierarchal clustering.

Modelling of ^13^C and ^15^N metabolite data and relative EAA consumption

Steady-state ^13^C flux analysis was performed on *in vitro* ^13^C-glutamine and ^13^C-glucose LC-MS data^14^. The enrichment fractions of αKG, malate, citrate, aspartate, glutamate and glutamine were used to calculate fluxes for a 25-reaction TCA cycle model by least-square optimisation (Table S2). Briefly, by adjusting flux parameters in the model, the optimisation exercise aims to simulate metabolite enrichments such that they are consistent with experimental data. The objective function was weighed using measurement errors. Enrichment fractions were corrected for natural enrichments of non-backbone carbon prior to optimisation. Labelled glutamine and unlabelled pyruvate were the main input substrates; unlabelled glutamine, glutamate, aspartate and succinate were included *ad hoc* to account for small influx of endogenous substrates. We assumed the purity of ^13^C label is 99%, and natural ^13^C enrichment is 1.07%. Residual errors were close to the acceptable cut-off for goodness-of-fit (χ^2^_49,0.05_ < 66): 75 for HCC1806 and 147 for MCF-7. We then generated flux distributions (i.e., sensitivity analysis) using a Monte-Carlo resampling technique. Briefly, the same optimisation exercise was performed on experimental data that has been randomly corrupted with 1 standard error gaussian noise. This was repeated 500 times for each condition, and compiled flux distribution were displayed as box plots. Optimisations were performed in MATLAB® R2018a. Code can be made available upon request.

We used an empirical kinetic model to summarise ^15^N enrichment data generated from our ^15^N-amide-glutamine labelling experiment. Model fitting in MATLAB was performed for asparagine, glutamine and nucleotides without corrections for natural enrichments (Fig. S7a,b). Nucleotides were grouped into the four nucleobases (A, G, U and C). The model is based on Michaelis-Menten equation to capture an asymptotic approach towards label saturation, to generate kinetic parameters representing maximum enrichment (analogous to V_max_) and time at half-maximum (analogous to K_m_) (Fig. S7c). Sensitivity analysis was performed by resampling 80% of the datapoints without replacement.

Relative EAA consumption (Fig. S3a) was calculated as a ratio of CORE uptake rate and amino acid composition of an average human cell was taken from Recon 3D^15, 16^. Histidine and cysteine rates were unavailable from CORE. Using doubling times from CORE and average mammalian cell dry weight of 360 pg/cell, cell-specific rates in fmol/cell/hr from CORE were converted to mmol/gDW to enable comparison against Recon 3D’s compositions.

**Supplementary References**

1 Hosios AM, Hecht VC, Danai LV, Johnson MO, Rathmell JC, Steinhauser ML *et al*. Amino Acids Rather than Glucose Account for the Majority of Cell Mass in Proliferating Mammalian Cells. Dev Cell 2016; 36: 540-549.

2 Quek LE, Liu M, Joshi S, Turner N. Fast exchange fluxes around the pyruvate node: a leaky cell model to explain the gain and loss of unlabelled and labelled metabolites in a tracer experiment. Cancer Metab 2016; 4: 13.

3 Yuan M, Breitkopf SB, Yang X, Asara JM. A positive/negative ion-switching, targeted mass spectrometry-based metabolomics platform for bodily fluids, cells, and fresh and fixed tissue. Nat Protoc 2012; 7: 872-881.

4 Lu W, Clasquin MF, Melamud E, Amador-Noguez D, Caudy AA, Rabinowitz JD. Metabolomic analysis via reversed-phase ion-pairing liquid chromatography coupled to a stand alone orbitrap mass spectrometer. Anal Chem 2010; 82: 3212-3221.

5 Curtis C, Shah SP, Chin SF, Turashvili G, Rueda OM, Dunning MJ *et al*. The genomic and transcriptomic architecture of 2,000 breast tumours reveals novel subgroups. Nature 2012; 486: 346-352.

6 Pereira B, Chin SF, Rueda OM, Vollan HK, Provenzano E, Bardwell HA *et al*. The somatic mutation profiles of 2,433 breast cancers refines their genomic and transcriptomic landscapes. Nat Commun 2016; 7: 11479.

7 Barretina J, Caponigro G, Stransky N, Venkatesan K, Margolin AA, Kim S *et al*. The Cancer Cell Line Encyclopedia enables predictive modelling of anticancer drug sensitivity. Nature 2012; 483: 603-607.

8 Cerami E, Gao J, Dogrusoz U, Gross BE, Sumer SO, Aksoy BA *et al*. The cBio cancer genomics portal: an open platform for exploring multidimensional cancer genomics data. Cancer Discov 2012; 2: 401-404.

9 Gao J, Aksoy BA, Dogrusoz U, Dresdner G, Gross B, Sumer SO *et al*. Integrative analysis of complex cancer genomics and clinical profiles using the cBioPortal. Sci Signal 2013; 6: pl1.

10 Prat A, Carey LA, Adamo B, Vidal M, Tabernero J, Cortes J *et al*. Molecular features and survival outcomes of the intrinsic subtypes within HER2-positive breast cancer. J Natl Cancer Inst 2014; 106.

11 Jiang G, Zhang S, Yazdanparast A, Li M, Pawar AV, Liu Y *et al*. Comprehensive comparison of molecular portraits between cell lines and tumors in breast cancer. BMC Genomics 2016; 17 Suppl 7: 525.

12 Subramanian A, Tamayo P, Mootha VK, Mukherjee S, Ebert BL, Gillette MA *et al*. Gene set enrichment analysis: a knowledge-based approach for interpreting genome-wide expression profiles. Proceedings of the National Academy of Sciences of the United States of America 2005; 102: 15545-15550.

13 Mootha VK, Lindgren CM, Eriksson KF, Subramanian A, Sihag S, Lehar J *et al*. PGC-1alpha-responsive genes involved in oxidative phosphorylation are coordinately downregulated in human diabetes. Nat Genet 2003; 34: 267-273.

14 Quek LE, Nielsen LK. Steady-state (1)(3)C fluxomics using OpenFLUX. Methods Mol Biol 2014; 1191: 209-224.

15 Jain M, Nilsson R, Sharma S, Madhusudhan N, Kitami T, Souza AL *et al*. Metabolite profiling identifies a key role for glycine in rapid cancer cell proliferation. Science 2012; 336: 1040-1044.

16 Brunk E, Sahoo S, Zielinski DC, Altunkaya A, Drager A, Mih N *et al*. Recon3D enables a three-dimensional view of gene variation in human metabolism. Nat Biotechnol 2018; 36: 272-281.
